# SMG7 and eIF4A constitute a homeostatic module controlling P-body condensation and function of meiotic bodies

**DOI:** 10.1038/s41467-026-72218-w

**Published:** 2026-04-21

**Authors:** Albert Cairo, Neha Shukla, Sofia Kanavorova, Jan Skalak, Pavlina Mikulkova, Anna Vargova, David Potesil, Zbynek Zdrahal, Jan Hejatko, Karel Riha

**Affiliations:** Central European Institute of Technology, Masaryk University, Brno, Czech Republic

**Keywords:** Plant cell biology, Plant reproduction, RNA metabolism

## Abstract

Processing bodies (P-bodies) are ribonucleoprotein condensates that regulate RNA processing and storage. Although constitutively present in most cells, their size and composition change dynamically in response to developmental and environmental cues. However, mechanisms governing P-body assembly and remodeling remain poorly understood. Here we show that in *Arabidopsis*, SMG7 interacts with the eIF4A helicases and recruits them to P-bodies. eIF4As limit P-body condensation and also restrict stress granule (SG) formation under heat stress. We further identify meiotic bodies (M-bodies) as composite RNP granules with a P-body core surrounded by a SG-like shell. The SMG7-eIF4A module regulates the recruitment of the meiosis-specific protein TDM1 into M-bodies, thereby influencing meiotic exit and plant reproduction. Our findings suggest that SMG7 functions as an adaptor protein that recruits client proteins into P-bodies and, together with eIF4A, forms a regulatory module that controls P-body composition and maintains their size homeostasis.

## Abstract (web summary)

eIF4A is actively recruited to P-bodies through the action of SMG7 where it restricts their growth under both normal and stress conditions, thereby ensuring proper cellular function and successful reproduction.

## Introduction

Ribonucleoprotein (RNP) granules, such as stress granules (SGs), P-bodies, or Cajal bodies, are assemblies of proteins and RNAs that are either stably present in cells or form in response to stress or developmental stimuli. These membrane-less organelles serve as dynamic hubs coordinating various steps of RNA biogenesis and processing, including transcription, splicing, storage, or translational repression, ensuring tight control over gene expression. RNP granules are held together by multivalent low-affinity interactions, allowing constant exchange of proteins and RNAs with their surroundings, which contributes to their dynamic nature^1,2^.

RNAs are not merely passive subjects of RNP granule regulation, but act as an important structural scaffold for their nucleation and assembly. As long flexible polymers, RNAs provide numerous sites for intermolecular interactions with either other RNAs or RNA-binding proteins, and facilitate the formation of higher order assemblies^3^. Notably, RNA exhibits an intrinsic propensity for self-assembly at relatively low concentrations in vitro, a characteristic that has been implicated in the nucleation of SGs. SGs are RNP granules that form when translationally repressed mRNAs are released from ribosomes during cellular stress^4–7^. Because the intracellular concentration of RNA surpasses the in vitro threshold for self-assembly, exposed RNAs can engage in promiscuous interactions that drive the spontaneous formation of SGs and other RNP condensates.

Uncontrolled RNA self-assembly can disrupt cellular homeostasis by sequestering essential RNA-binding proteins, interfering with RNA processing, or promoting the formation of aberrant condensates. Recent studies in yeast and human cells have suggested the existence of an RNA chaperone network that counteracts the formation of these assemblies, facilitates RNP remodeling, and regulates RNP granule homeostasis^6,8–11^. These processes can control the RNA flux between condensates and the surrounding cytoplasm, thereby influencing mRNA availability and translatability^11,12^. One example of an RNA chaperone is eIF4A, which is primarily known for its role in translation initiation. eIF4A is the archetypal member of the DEAD-box helicase family and, together with the scaffold protein eIF4G and the mRNA 5’ cap-binding protein eIF4E, forms the eukaryotic translation initiation complex eIF4F^13–15^. eIF4A functions by unwinding structures within the 5’-UTR of mRNAs, thereby facilitating the scanning for start codons by the 43S preinitiation ribosome complex^16^. Separate from its function in translation, eIF4A has also been shown to limit RNA condensation and SG formation in human cells^6^.

P-bodies are another type of RNP condensate related to SGs that accumulate translationally repressed mRNAs. However, unlike SGs, P-bodies are typically constitutively present in cells and are enriched in proteins involved in mRNA degradation, such as nonsense-mediated RNA decay (NMD) factors, decapping enzymes, exonucleases, and various RNA-binding proteins, which collectively regulate mRNA turnover and storage^17,18^. In plants, P-bodies have been implicated in a number of biological processes, including pathogen defense, photomorphogenesis, actin remodeling, heat stress adaptation, and meiotic progression^19–23^. Although present in most plant cells, P-bodies are highly dynamic structures, and their size and composition change in response to environmental and developmental stimuli^19,23–26^. This suggests the existence of active remodeling mechanisms that regulate P-body assembly, disassembly, and compositional plasticity, allowing cells to fine-tune mRNA metabolism according to changing physiological demands.

SMG7 is an evolutionarily conserved NMD factor and a core component of P-bodies in both mammals and plants^21,27^. In NMD, SMG7 binds to the phosphorylated UPF1 helicase through its 14-3-3-like domain and recruits it to P-bodies^28–30^. In *Arabidopsis*, SMG7 also fulfills an NMD-independent function in meiotic exit^31,32^. At the end of meiosis, SMG7 binds and recruits the meiosis-specific protein TDM1 into P-bodies. TDM1 interacts with the translation initiation factor eIFiso4G2 and sequesters it in P-bodies, thereby inhibiting translation and facilitating meiotic exit^23^. Thus, during meiosis, SMG7 changes the composition of P-bodies and alters their function to temporarily inhibit translation and allow meiotic exit.

14-3-3 proteins are known to interact with a broad spectrum of substrates^33^, raising the possibility that SMG7, which contains an N-terminal 14-3-3-like domain, may associate with additional proteins beyond TDM1 and UPF1. In this study, we identify the *Arabidopsis* DEAD-box RNA helicases eIF4A1 and eIF4A2 as SMG7 interactors and uncover a previously unrecognized role for SMG7 in P-body remodeling. Similar to its interactions with UPF1 and TDM1, SMG7 binds to eIF4A1/2 and recruits them to meiotic P-bodies. We further show that meiotic cells contain specialized cytoplasmic RNP condensates, M-bodies, consisting of a P-body core surrounded by a shell enriched with SG components. Consistent with their RNA chaperone activity, eIF4A1/2 limit the size of both SGs and P-bodies in somatic cells, as well as the size of M-bodies, thereby influencing their capacity to recruit TDM1 and to promote meiotic exit. We propose that SMG7-eIF4A constitutes an autoregulatory module that controls P-body remodeling and homeostasis.

## Results

### SMG7 interacts with the RNA helicase eIF4A in P-bodies

**Fig. 1:**
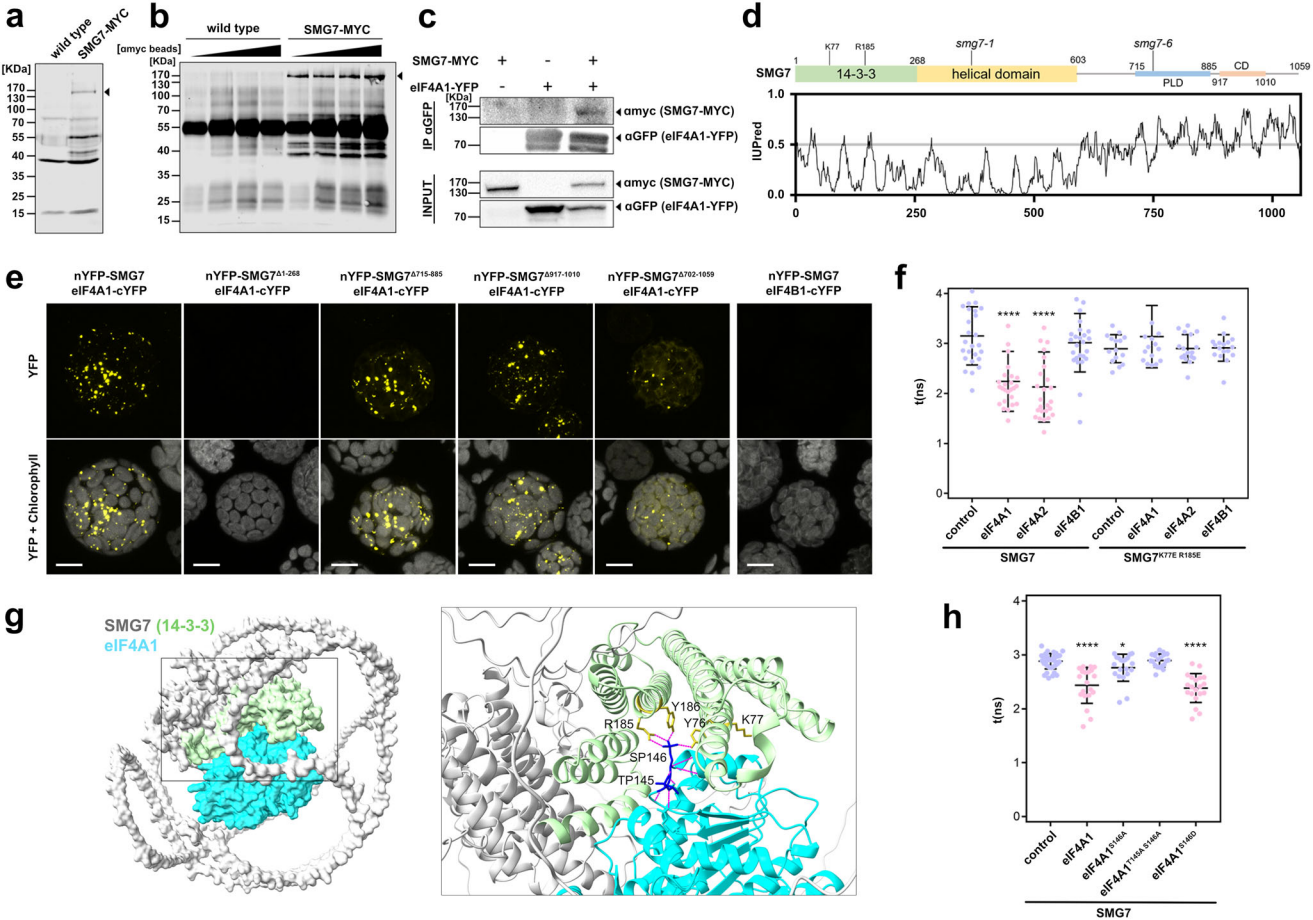
SMG7 interacts with the RNA helicase eIF4A in P-bodies. **a** Western blot analysis of SMG7-MYC expression in *SMG7:MYC* plants. **b** Western blot analysis of immunoprecipitates of protein extracts of wild-type and SMG7:MYC plants using increasing amounts of anti-MYC antibody. **c** Co-immunoprecipitation of SMG7-MYC and eIF4A-YFP from plants expressing either SMG7-MYC, eIF4A1-YFP, or both constructs using anti-GFP antibody. SMG7-MYC was detected with anti-MYC antibody, and eIF4A1 with anti-GFP antibody. The images of (**a**–**c**) are representative of three different experiments. **d** SMG7 domain structure indicating the T-DNA insertion site of the mutants *smg7-1* and *smg7-6*. Prediction of disordered regions using IUPred^86^ is depicted below. **e** BiFC assay in *Arabidopsis* mesophyll protoplasts of eIF4A1 interaction with the mutated versions of SMG7. The micrographs are representative of multiple observations in more than three different experiments. Scale bar, 10 μm. **f** Fluorescence lifetime measured in a FLIM-FRET interaction assay using the indicated protein combinations transiently expressed in *N. tabacum* leaves (mean, SD, *n* = 15–26. ****P < 0.0001, two-tailed unpaired Student’s *t* test). **g** AF model of the SMG7-eIF4A1 interaction interface prediction. SMG7 is represented in gray with the 14-3-3 domain highlighted in green. eIF4A1 is represented in cyan. The right panel shows a close-up view of the interaction. The magenta line represents predicted hydrogen bonds. **h** Fluorescence lifetime measured in a FLIM-FRET interaction assay using the indicated protein combinations transiently expressed in *N. tabacum* leaves (mean, SD, *n* = 20–38 FRET events. *P < 0.05, ****P < 0.0001, two-tailed unpaired Student’s *t* test). Source data are provided as a Source Data file.

To identify SMG7 interactors, we used the *Arabidopsis* line expressing SMG7-MYC (Fig. 1a), which complements the *smg7-1* null mutation^32,34^, for immunoprecipitation (Fig. 1b) followed by mass spectrometry. Among the most enriched proteins were the two isoforms of eIF4A, eIF4A1 and eIF4A2 (Supplementary Table 1). The *Arabidopsis* genome encodes three eIF4A paralogues. The cytoplasmic eIF4A1 and eIF4A2 act in translation and share 97% sequence similarity and similar expression patterns^35^, whereas eIF4A3 is part of the exon-junction complex and shuttles between the nucleus and cytoplasm^36^. While the *eif4a1* and *eif4a2* single mutants, which carry T-DNA insertions in the respective genes, are viable and show no phenotype, aside from slightly impaired growth observed in *eif4a1*, the simultaneous loss of both genes is lethal, indicating a substantial functional redundancy^35^. To validate the interaction, we generated the *eIF4A1:YFP* line containing the genic region of *eIF4A1* fused to YFP that complements the *eif4a1* phenotype, and crossed it with the *SMG7:MYC* line. Co-immunoprecipitation of SMG7-MYC with the GFP nanobeads from plants expressing both constructs indicates that SMG7 and eIF4A1 indeed interact (Fig. 1c).

Next, we used bimolecular fluorescence complementation (BiFC) in mesophyll protoplasts to dissect the interaction of SMG7 with eIF4A1 and eIF4A2 (hereafter referred to as eIF4A1/2). Co-transfection of nYFP-SMG7 with eIF4A1/2-cYFP produced a strong YFP signal, whereas no signal was detected in the control, where nYFP-SMG7 was co-transfected with another subunit of the eIF4F complex, eIF4B1-cYFP (Fig. 1e and Supplementary Fig. 1a). SMG7 consists of an evolutionarily conserved N-terminal 14-3-3-like domain and a central helical domain^30^, while the C-terminal half is highly diverged and intrinsically disordered (Fig. 1d)^23^. This region includes a prion-like domain and several motifs at the very C-terminus (CD), which are well conserved among plant SMG7 proteins^23,37^. To map the SMG7 region required for the interaction with eIF4A1/2, we generated a series of deletion constructs. While deletions spanning the prion-like domain, the CD, or the entire C-terminus produced a BiFC signal, deletion of the 14-3-3 domain entirely abolished the signal (Fig. 1e and Supplementary Fig. 1a).

eIF4A1/2 is uniformly distributed throughout the cytoplasm of mesophyll protoplasts (Supplementary Fig. 1b), whereas the eIF4A1/2-SMG7 BiFC signal appears in distinct cytoplasmic speckles. We have previously shown that SMG7 localizes to P-bodies, and that the deletion of its C-terminal intrinsically disordered region (IDR) (SMG7^Δ702-1059^) reduces its partitioning into P-bodies (Supplementary Fig. 1c)^23^. This suggests that the majority of SMG7-eIF4A1/2 interaction occurs in P-bodies and reflects the subcellular localization of SMG7. This conclusion is further supported by the observation that the interaction with truncated SMG7^Δ702-1059^ is detectable in speckles as well as in cytoplasm (Fig. 1e). We also noticed that the deletion of the N-terminal domain abolished the P-body localization of SMG7 (Supplementary Fig. 1c), indicating that its partitioning into P-bodies is primarily mediated by interactions involving the α-helical 14-3-3 and reinforced by the C-terminal IDRs.

The 14-3-3 domain forms a binding pocket that recognizes phosphoserine residues of phosphorylated UPF1, with the interaction coordinated by evolutionarily conserved lysine and arginine residues^29,30^. To determine whether eIF4A1/2 binding to SMG7 occurs through this pocket, we employed Förster resonance energy transfer by fluorescence lifetime microscopy (FLIM-FRET), with SMG7 mutant in the conserved Lys^77^ and Arg^185^ residues (SMG7^K77E R185E^) known to abolish the phosphoserine binding^29,30^. We observed a shortening of the YFP fluorescence lifetime of SMG7-YFP during co-expression with eIF4A1/2-TagRFP constructs, further confirming their interaction (Fig. 1f). In contrast, the fluorescence lifetime remained unchanged when YFP-SMG7^K77E R185E^ was co-expressed with eIF4A1/2-TagRFP, indicating that these residues are essential for the interaction.

These data indicate that phosphorylation of eIF4A1/2 may facilitate the interaction with SMG7. A search in the PhosPhat 4.0 database^38^ for previously identified phosphosites in eIF4A1 and eIF4A2 revealed consistent phosphorylation at Ser^4^ in the N-terminus, and at Thr^145^ and Ser^146^ in the N-helicase domain, across several independent data sets. To further explore the interaction interface, we used AlphaFold 3 (AF)^39^ to model the SMG7–eIF4A1 complex, incorporating these three phosphorylation sites. The resulting AF model revealed an interaction interface between phosphorylated eIF4A1 and the 14-3-3 domain of SMG7, in which phosphorylated Ser^146^ forms a hydrogen bond with Arg^185^ of SMG7 (Fig. 1g), supporting our FLIM-FRET data (Fig. 1f). Although the Lys^77^ of SMG7 was not predicted to be directly involved in the interaction, its neighboring residue Tyr^76^, together with the spatially proximal Tyr^186^ form hydrogen bonds with phospho-Ser^146^ of eIF4A1. Phosphorylated Thr^145^ of eIF4A1 is not directly involved in the interaction interphase in the model, but it may contribute to the stabilization of the helicase loop of eIF4A1, which is engaged in the 14-3-3 binding pocket of SMG7 (Fig. 1g).

We experimentally validated the AF model using FLIM-FRET and substitutions of Thr^145^ and Ser^146^ in eIF4A1. The interaction with SMG7 was significantly reduced in the non-phosphorylatable eIF4A1^S146A^ mutant and completely abolished when both Thr^145^ and Ser^146^ were mutated (eIF4A1^T145A S146A^; Fig. 1h). In contrast, the phospho-mimetic substitution eIF4A1^S146D^ displayed an interaction strength comparable to that of the wild-type eIF4A1. In conclusion, these results demonstrate that SMG7 interacts with eIF4A1/2, and that this interaction is mediated by the 14-3-3 binding pocket of SMG7 and the Thr^145^-Ser^146^-containing interface within the N-helical domain of eIF4A. The FLIM-FRET data further suggest that this interaction is facilitated by the phosphorylation at Ser^146^.

### eIF4A1/2 localize to P-bodies and SGs

**Fig. 2:**
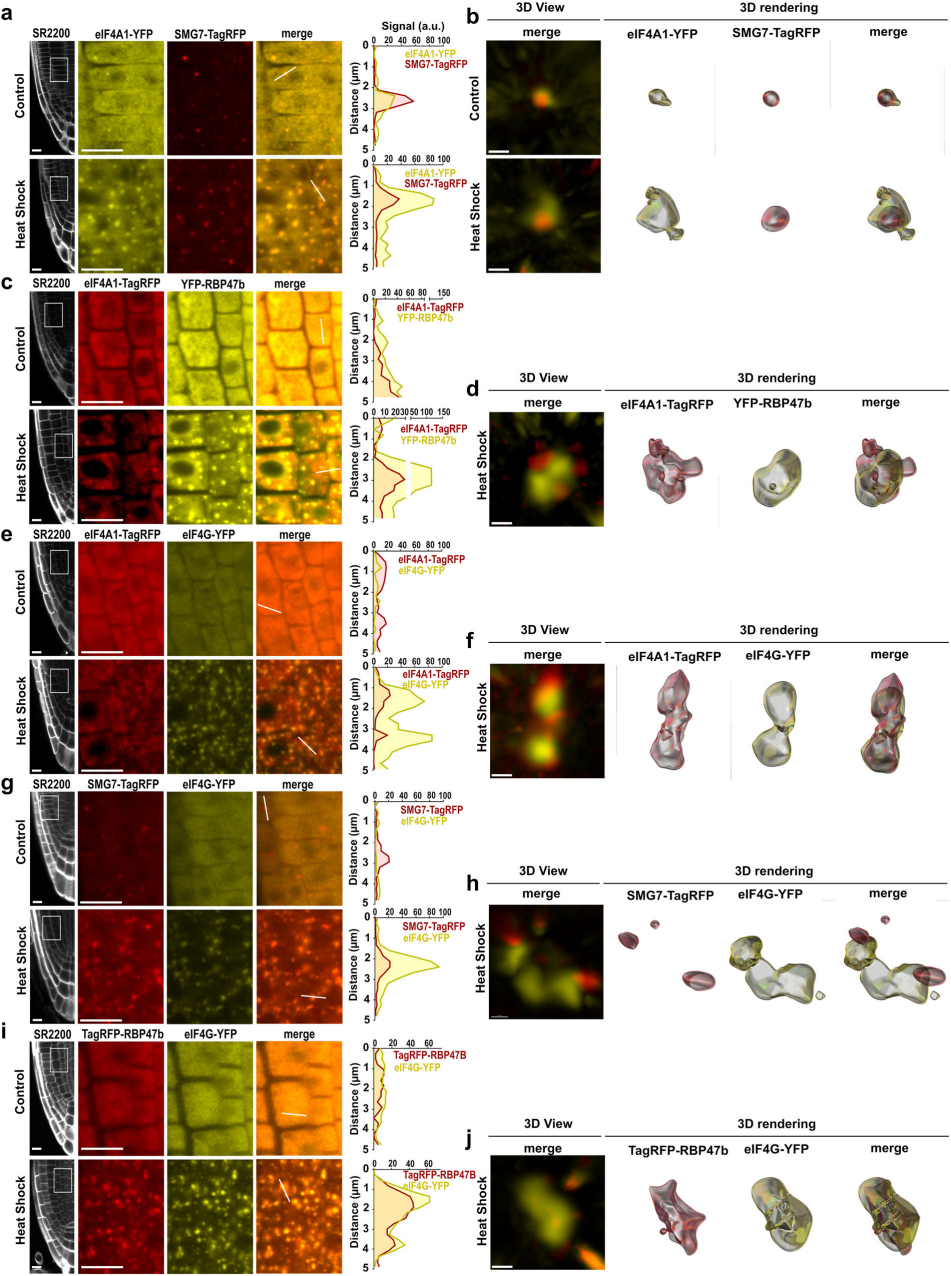
eIF4A1 colocalizes to P-bodies and SGs. **a**, **c**, **e**, **g**, **i** Confocal micrographs of root cells co-expressing indicated combinations of reporter proteins. Counterstaining with SR2200 dye was used to visualize cell walls. A minimum of three root tips were subjected to analysis, with each micrograph serving as a representative sample. Diagrams on the right show superimposed intensity profiles of YFP and TagRFP signals measured along the lines indicated in the corresponding micrographs. Each diagram serves as a representative of multiple observations. Scale bar =  10 μm. **b**, **d**, **f**, **h**, **j** Super-resolution micrographs of indicated protein condensates visualized by 3D view and 3D rendering using Imaris software. Scale bar =  0.5 μm. Source data are provided as a Source Data file.

We next performed colocalization studies in the roots of *Arabidopsis* lines expressing SMG7-TagRFP^23^ along with either eIF4A1-YFP or eIF4A2-YFP. Confocal microscopy showed prominent localization of SMG7-TagRFP to P-bodies (Fig. 2a). While the majority of the eIF4A1/2-YFP signal was diffusely distributed throughout the cytoplasm, we also observed distinct foci that colocalized with the larger SMG7-TagRFP-marked P-bodies (Fig. 2a and Supplementary Figs. 2a and 3a). P-bodies are known to increase in size under certain stress conditions, such as heat-shock^24,40^. Indeed, exposure of roots to 39 °C for 30 min led to a significant increase in both the size and abundance of SMG7-TagRFP labeled P-bodies (Fig. 2a, g and Supplementary Fig. 2a, g). Heat shock also significantly enhanced the partitioning of eIF4A1/2-YFP into cytoplasmic speckles, which colocalized with SMG7-TagRFP (Fig. 2a and Supplementary Figs. 2a and 3a). These changes were reversible, as P-bodies returned to their pre-stress state within 3 h after the plants were shifted back to normal temperature (Supplementary Fig. 3b, c).

The subunits of the eIF4F typically associate with SGs, and *Arabidopsis* eIF4A2 localizes to SGs upon heat shock and salt stress^41^. Since SGs frequently associate with P-bodies^8,42–44^, we employed structured illumination super-resolution microscopy to distinguish the localization of eIF4A1/2 within these subcellular compartments. Under normal temperature conditions, eIF4A1/2–YFP foci partially overlapped with the SMG7–TagRFP signal, confirming the localization of eIF4A1/2 to P-bodies. However, the eIF4A1/2–YFP signal extended beyond the SMG7–TagRFP region, and this expansion became even more apparent following heat shock treatment (Fig. 2b and Supplementary Figs. 2b and 3d). These observations suggest that, in addition to localization to P-bodies, heat shock promotes the expansion of eIF4A1/2 condensates beyond P-bodies, likely into associated SGs.

To test this possibility, we generated double reporter lines expressing YFP-RBP47b, an *Arabidopsis* SG marker^24,40,45^, driven by the ribosomal pRPS5A promoter, together with eIF4A1-TagRFP or eIF4A2-TagRFP expressed from their native promoters. Under normal conditions, YFP-RBP47b exhibits diffused cytoplasmic localization, but upon heat shock it partitions into prominent SGs (Fig. 2c). Super-resolution microscopy revealed that the heat-induced YFP-RBP47b signal partially overlaps with the eIF4A1/2-TagRFP (Fig. 2d and Supplementary Figs. 2d and 3e). Importantly, colocalization analysis of SGs and P-bodies in heat-stressed plants expressing YFP-RBP47b and SMG7-TagRFP showed that although these two proteins form closely associated granules, their signals do not overlap and occupy distinct territories (Supplementary Figs. 2e, f and 3f). These data indicate that under heat shock conditions, eIF4A1/2 localize to both SGs and P-bodies.

We next sought to determine whether localization to P-bodies is specific to eIF4A1/2, or whether it represents a general feature of the eIF4F complex in plants. Similar to YFP-RBP47b, eIF4G-YFP^23^ displayed diffused cytoplasmic localization in root cells under normal conditions, but relocalized to cytoplasmic speckles following heat-shock treatment (Fig. 2e, g, i). Interestingly, despite being part of the same complex, eIF4A1/2 speckles only partially overlapped with those of eIF4G (Fig. 2f and Supplementary Figs. 2h and 3e). While heat-induced eIF4G speckles associated with SMG7-marked P-bodies, they formed distinct subdomains with a little overlap (Fig. 2g, h and Supplementary Fig. 3f), suggesting that eIF4G partitions into SGs. This was confirmed in the double reporter line expressing TagRFP-RBP47b and eIF4G-YFP, where both markers colocalized in the same heat-induced granules (Fig. 2i, j and Supplementary Fig. 3f).

In conclusion, these colocalization experiments show that eIF4A1/2 have the capacity to partition into both P-bodies and SGs, a feature that distinguishes them from their interaction partner eIF4G, which localizes exclusively to SGs under stress conditions.

### eIF4A1/2 limit the condensation of SGs and P-bodies in *Arabidopsis* root cells

**Fig. 3:**
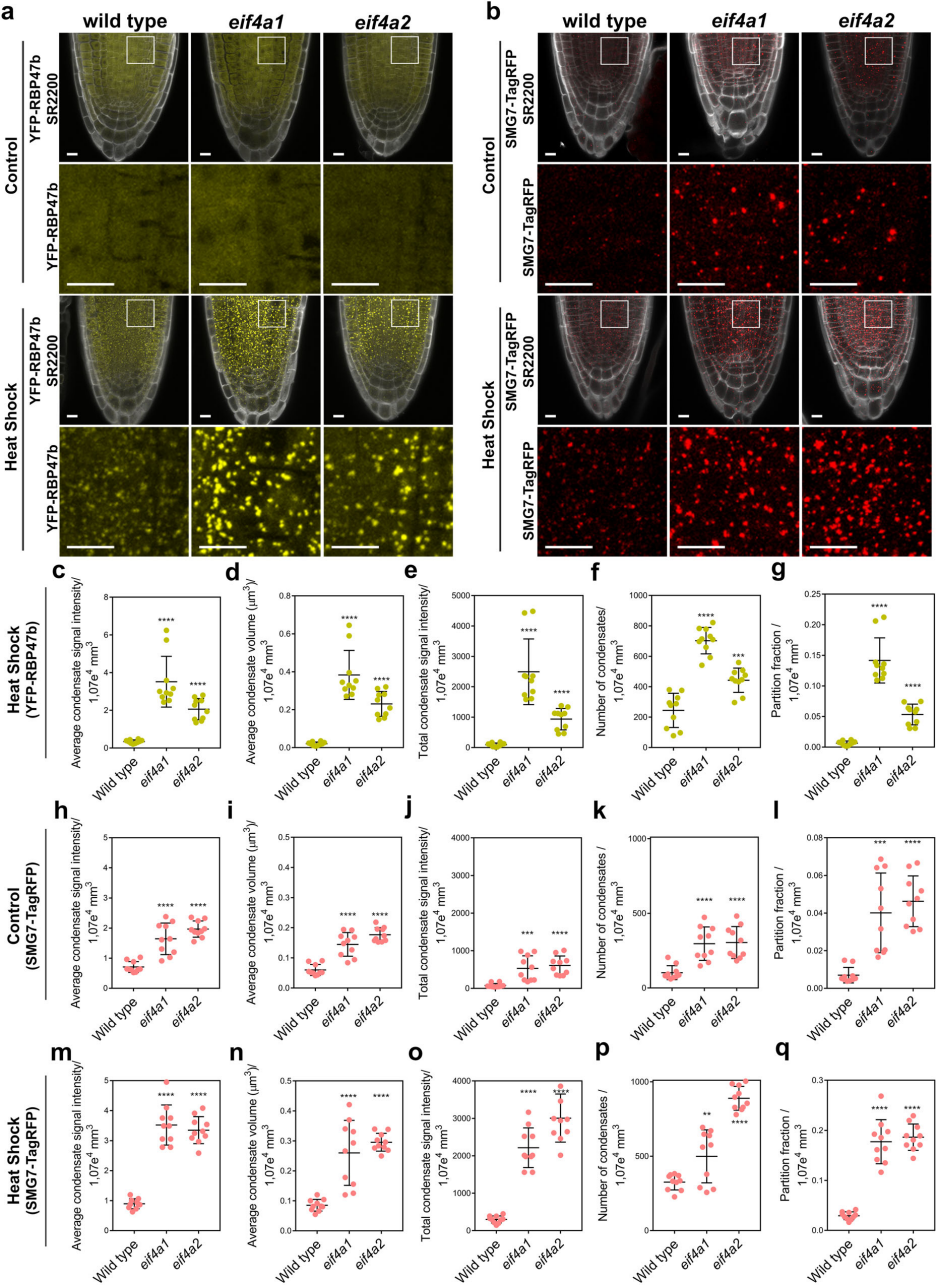
eIF4A1/2 limit the condensation of SGs and P-bodies in *Arabidopsis* root cells. **a**, **b** Confocal micrographs of *Arabidopsis* roots from wild-type and *eif4a1* and *eif4a2* mutants expressing YFP-RBP47b (**a**) or SMG7-TagRFP (**b**) under heat shock and control conditions. Cell walls were visualized using SR2200 counterstaining. Rectangles indicate the cell areas used for quantitative analyses of condensates. Scale bars =  10 μm. **c**–**q** Dot plots showing average signal intensity per condensate (**c**, **h**, **m**), average condensate volume (**d**, **i**, **n**), total condensate signal (**e**, **j**, **o**), and number of condensates (**f**, **k**, **p**) detected with in a volume of 1.07e^4^ μm^3^ encompassing endodermic and cortical cells**. g**, **l**, **q** Charts showing the fraction of a signal partitioning into condensates relative to the total signal in the defined root volume. (mean ± SD, *n* = 10 root tip volumes; **P < 0.01, ***P < 0.001, ****P < 0.0001, two-tailed unpaired Student’s *t* test). Source data are provided as a Source Data file.

In addition to its role in translation, eIF4A functions as an RNA chaperone, reducing SG condensation in human cells by limiting RNA–RNA interactions^6^. To determine whether eIF4A1/2 influences SG dynamics in plants, we analyzed the condensation of RBP47b in *eif4a1* and *eif4a2* mutants following heat shock treatment. In both mutants, the fraction of RBP47b signal in SGs increased, accompanied by a greater number and larger size of RBP47b-labeled condensates, as well as a higher total condensate signal per unit of volume (Fig. 3a, c–g). These results indicate that eIF4A1/2 limit SG condensation during heat stress in *Arabidopsis*.

We next investigated whether eIF4A1/2 also modulate P-body condensation by examining the SMG7-TagRFP signal in the *eif4a1* and *eif4a2* mutants (Fig. 3b, h–q). Under standard conditions, the average size of P-bodies, and their number and total signal intensity per cell volume were elevated in the mutants (Fig. 3h–l). This effect was even more pronounced following heat shock, where deficiency of either eIF4A isoform led to a marked increase in P-body signal compared to wild-type (Fig. 3m–q). Despite the pronounced effect on P-body size, the mobility of SMG7, assessed by fluorescence recovery after photobleaching (FRAP), was not substantially affected in *eif4a1* root tips (Supplementary Fig. 4). This finding is consistent with the idea that eIF4A primarily modulates RNA condensation^6^, whereas SMG7 mobility within P-bodies may be largely determined by protein–protein interactions. Together, these findings suggest that the RNA helicases eIF4A1 and eIF4A2 act to restrain the condensation of both P-bodies and SGs in *Arabidopsis*, supporting a conserved RNA chaperone function across eukaryotes.

### eIF4A1/2 localize to meiotic P-bodies and modulate their size

**Fig. 4:**
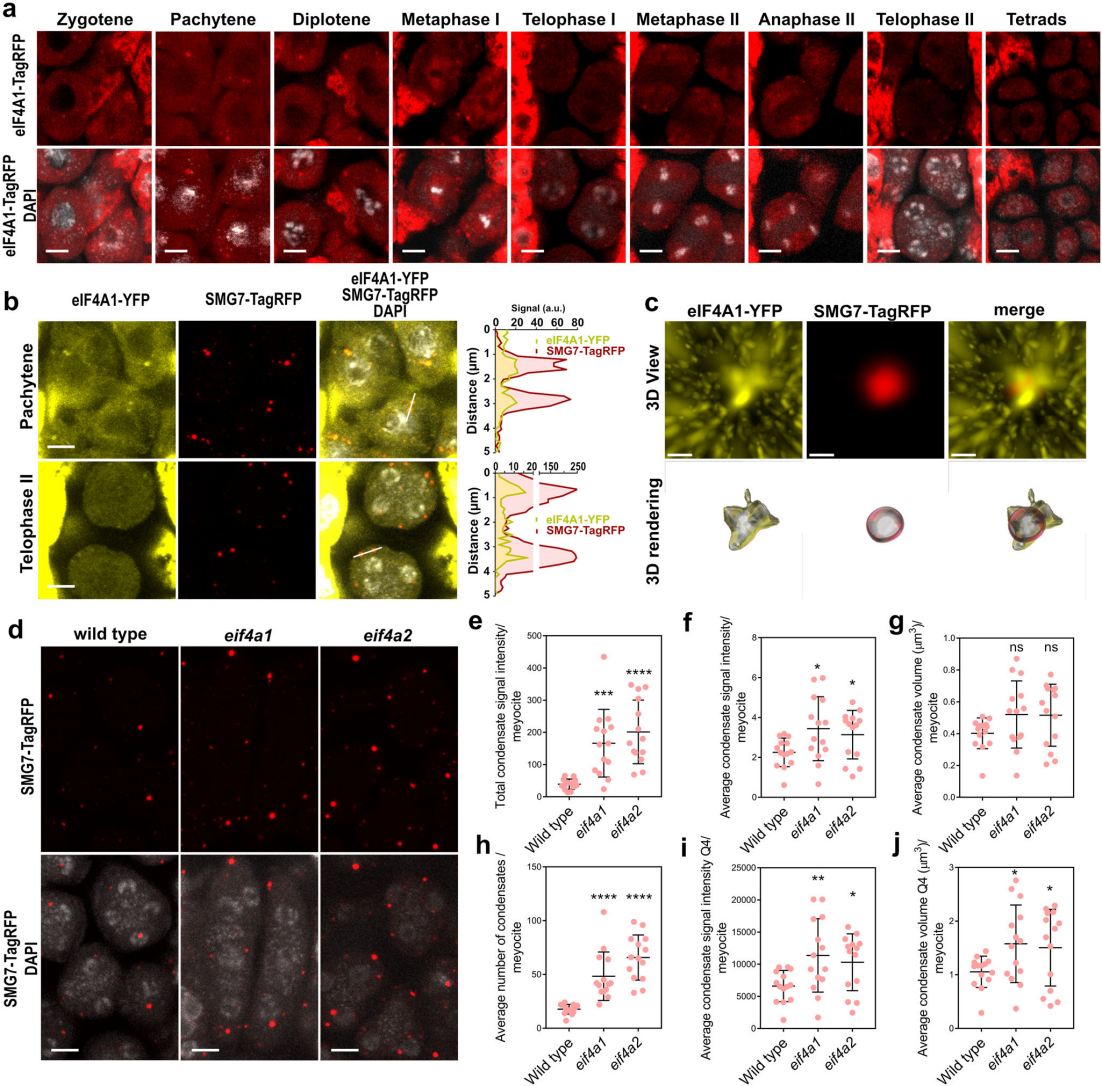
eIF4A1/2 localize to meiotic P-bodies and modulate their condensation. **a** Confocal micrographs of *Arabidopsis* anthers showing the localization of eIF4A1-TagRFP during meiosis. DNA was counterstained with DAPI. Each micrograph serves as a representative image of multiple observations (>20). Scale bars = 5 μm. **b** Confocal micrographs of *Arabidopsis* meiocytes co-expressing eIF4A1-TagRFP and SMG7-TagRFP. Micrographs depicting pachytene and telophase II meiocytes are shown and are representative of multiple observations (> 20). Diagrams on the right show superimposed intensity profiles of YFP and TagRFP signals measured along the lines indicated in the corresponding micrographs. Each diagram serves as a representative of multiple observations. Scale bar = 10 μm. **c** Super-resolution micrographs of indicated protein condensates corresponding to pachytene meiocytes visualized in 3D view and 3D rendering using Imaris software. Scale bar = 0.5 μm. **d** Confocal micrographs of *Arabidopsis* telophase II meiocytes of wild-type, *eif4a1*, and *eif4a2* plants expressing SMG7-TagRFP. Scale bars, 5 μm. **e**–**j** Dot plots showing total SMG7 condensate signal intensity in a meiocyte (**e**), average condensate intensity (**f**), condensate volume (**g**) and number of condensates per meiocyte, as well as average signal intensity (**i**) and volume (**j**) for the largest 25% of condensates in each meiocyte (mean SD, *n* = 14 telophase II meiocytes; ns ≥ 0.05,*P < 0.05, **P < 0.01 ***P < 0.001, ****P < 0.0001, two-tailed unpaired Student’s *t* test). Source data are provided as a Source Data file.

We previously demonstrated that efficient partitioning of SMG7 into P-bodies is essential for termination of meiosis and the transition to post-meiotic development of *Arabidopsis* microspores^23^. Given the influence of eIF4A1/2 on P-body size and their physical interaction with SMG7, we investigated their function during male meiosis. Confocal microscopy of anthers from *eIF4A1:TagRFP* and *eIF4A2:TagRFP* plants revealed a strong signal in the tapetum, the innermost cell layer of the anther locule, likely reflecting a high translation rate in these metabolically highly active cells (Fig. 4a and Supplementary Fig. 5a).

In meiocytes, eIF4A1/2 formed distinct foci that were apparent from zygotene (Fig. 4a and Supplementary Figs. 5a and 6a) and colocalized with SMG7 (Fig. 4b, c and Supplementary Figs. 5b, c and 6d). The SMG7-marked P-bodies in meiocytes were significantly larger than those observed in root and tapetal cells (Supplementary Fig. 5d–f). Throughout meiosis, eIF4A1 foci were less abundant than SMG7 foci (Supplementary Fig. 6a, b), and while all eIF4A1-positive sites overlapped with SMG7, only ~75% of SMG7 foci colocalized with eIF4A1 (Supplementary Fig. 6d). eIF4A1 signal preferentially associated with the larger SMG7 foci and was not detectable at the smaller foci (Supplementary Fig. 6e, f).

The strong partitioning of eIF4A1/2 to meiotic P-bodies suggests a regulatory role in maintaining their size homeostasis. To test this, we analyzed the condensation of SMG7-marked P-bodies in meiocytes of wild-type, *eif4a1*, and *eif4a2* mutants. Similar to our observations in root cells, P-bodies in meiocytes lacking either helicase were significantly more abundant and larger than those in the wild-type (Fig. 4d–j).

### Composite RNP granules consisting of a P-body core and a SG shell form during meiosis

**Fig. 5:**
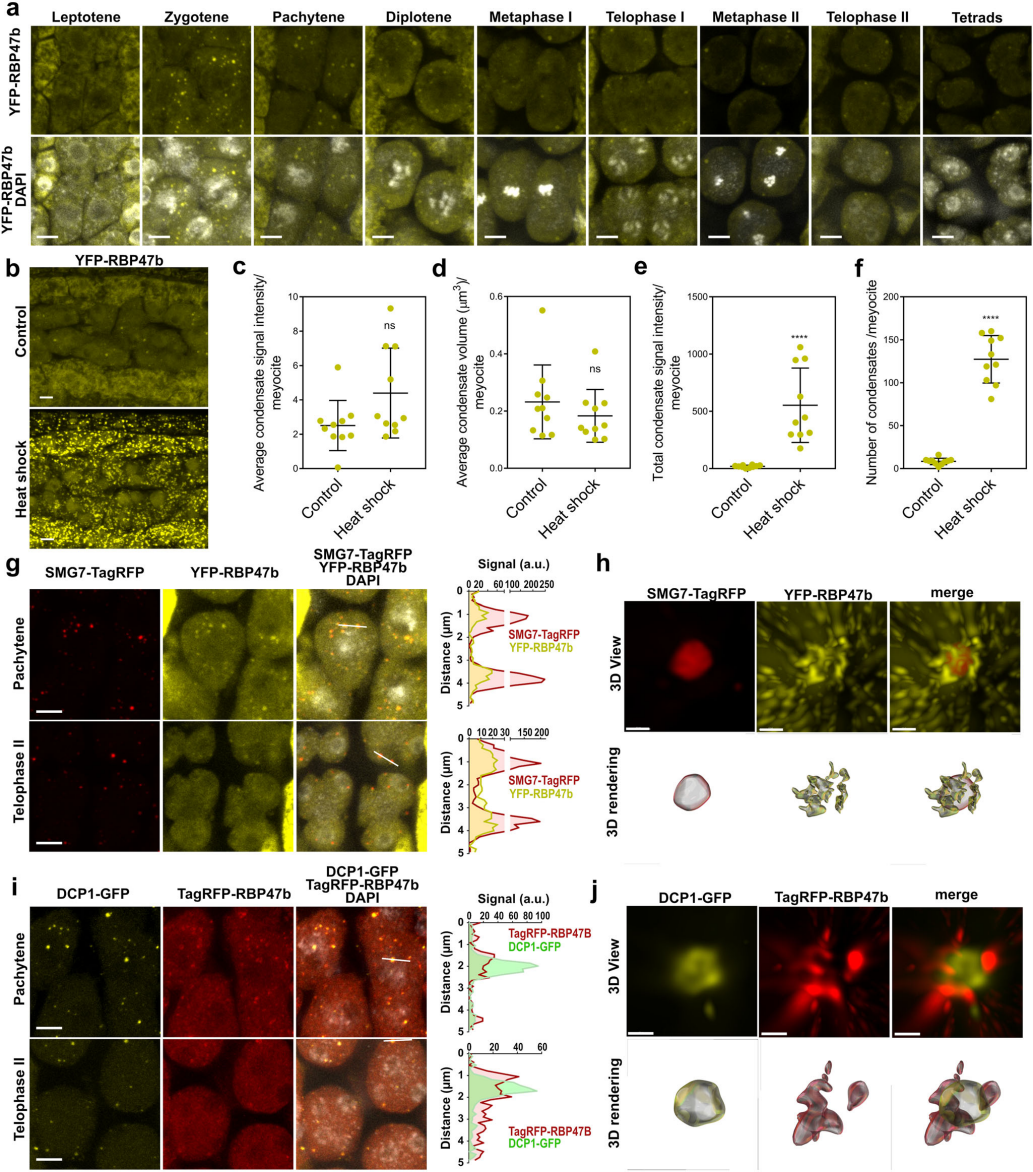
Composite RNP granules consisting of a P-body core and a SG shell form during meiosis. **a** Confocal micrographs of *Arabidopsis* pollen mother cells depicting the expression and localization of YFP-RBP47b in the course of meiosis. DNA is counterstained with DAPI. Each micrograph serves as a representative image of multiple observations (> 20). Scale bars = 5 μm. **b** Confocal micrographs of anther lobes, showing expression and localization of YFP-RBP47b in pachytene meiocytes and the surrounding tapetum upon heat shock or under control conditions without heat shock application. Scale bar = 5 μm. **c**–**f** Dot plots showing the average signal intensity (**c**) and average volume (**d**) of condensates from meiotic cells, total signal intensity in all condensates per meiotic cell (**e**), and the average number of condensates per meiocyte (**f**) (mean, SD, *n* = 10 zygotene meiocytes; ns ≥ 0.05, ****P < 0.0001, two-tailed unpaired Student’s *t* test). **g** Confocal micrographs of *Arabidopsis* meiocytes co-expressing SMG7-TagRFP and YFP-RBP47b. Micrographs depicting pachytene and telophase II meiocytes are shown, and are representative of multiple observations (>20). Diagrams on the right show superimposed intensity profiles of YFP and TagRFP signals measured along the lines indicated in the corresponding micrographs. Each diagram serves as a representative of multiple observations. Scale bar  = 5 μm. **h** Super-resolution micrographs of SMG7-TagRFP/YFP-RBP47b condensates corresponding to pachytene meiocytes visualized by 3D view and 3D rendering using Imaris software. Scale bar = 0.5 μm. **i** Confocal micrographs of *Arabidopsis* meiocytes co-expressing DCP1-GFP and YFP-RBP47b. Micrographs depicting pachytene and telophase II meiocytes are shown, and are representative of multiple observations (> 20). Diagrams on the right show superimposed intensity profiles of GFP and TagRFP signals measured along the lines indicated in the corresponding micrographs. Each diagram serves as a representative of multiple observations. Scale bar = 5 μm. **j** Super-resolution micrographs of DCP1-GFP/YFP-RBP47b condensates corresponding to pachytene meiocytes visualized by 3D view and 3D rendering using Imaris software. Scale bar = 0.5  μm. Source data are provided as a Source Data file.

Meiotic P-bodies exhibit distinct characteristics compared to those observed in somatic cells. Their larger size (Supplementary Fig. 5d–f) and enhanced recruitment of eIF4A1/2 (Fig. 4b, c and Supplementary Figs. 5b,c and 6d) resemble the P-bodies formed in response to heat shock in root cells. This prompted us to investigate whether these meiotic condensates also associate with SG markers. Confocal microscopy of anthers from the *YFP:RBP47b* line revealed cytoplasmic speckles in meiocytes at normal temperature, whereas the YFP-RBP47b signal appeared more diffuse in the surrounding tapetal cells (Fig. 5a). Nucleation of YFP-RBP47b speckles was observed throughout all meiotic stages, beginning in late leptotene, becoming particularly prominent from zygotene, and remaining visible through the tetrads stage (Fig. 5a and Supplementary Fig. 6c). Although heat shock increased the number of RBP47b speckles in meiocytes, their size and signal intensity remained comparable to those observed at control temperature (Fig. 5b–f).

**Fig. 6:**
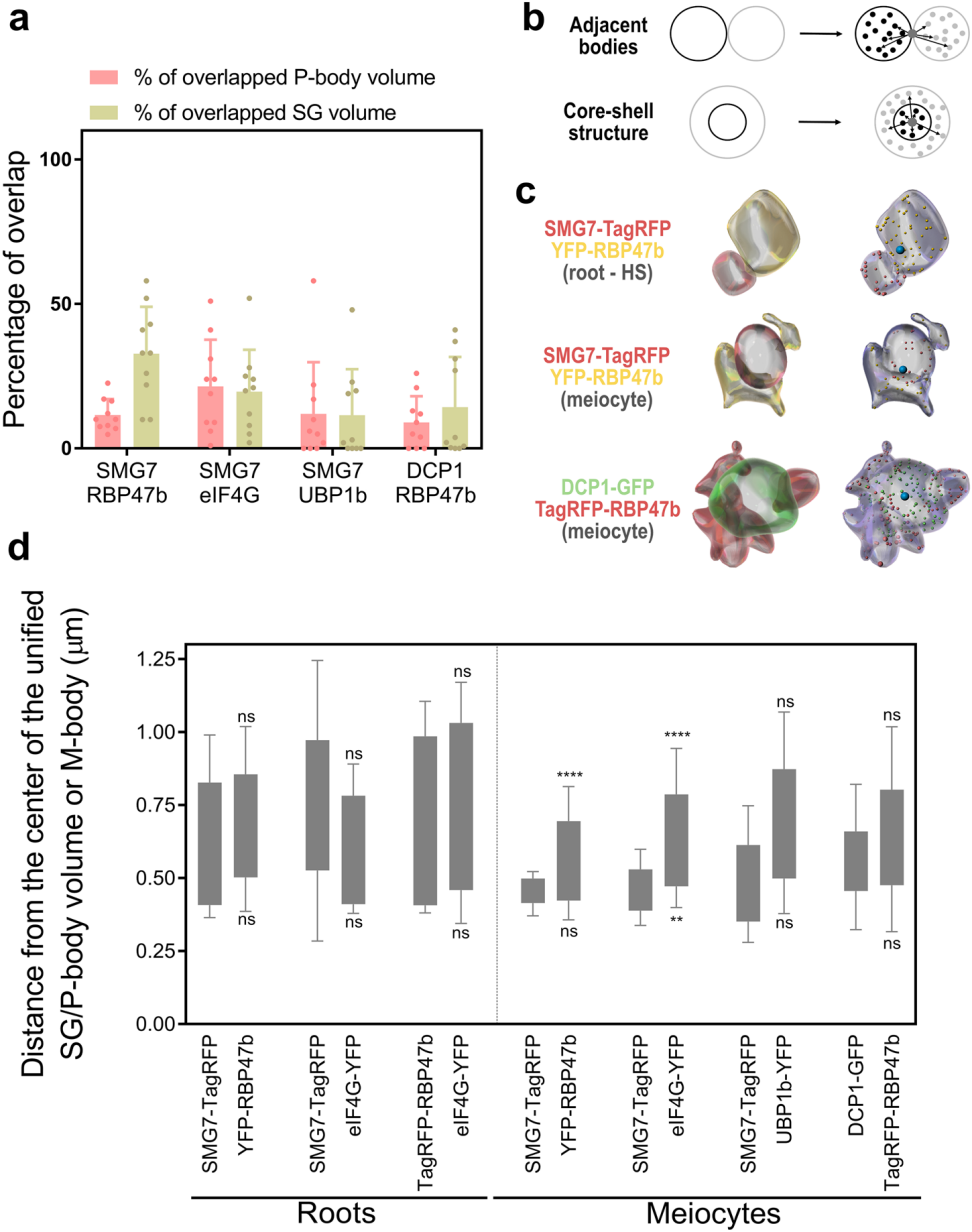
Structure of M-bodies. **a** Percentages of volume overlaps between P-body (SMG7, DCP1) and SG (RBP47b, eIF4G, UBP1b) markers in meiocytes visualized by super‑resolution microscopy (mean, SD, *n* = 10 associated condensates). **b**–**d** Analysis of the geometry of associated condensates. **b** Schematic representation of the workflow used to distinguish adjacent condensates from core–shell organization, based on the distances of individual condensates from the geometric center of their merged volumes. **c** 3D-segmented condensates from super-resolution micrographs (left) were decomposed based on signal density to generate spot maps representing condensate volume and intensity (right). Geometrical centers of the merged volumes are indicated by blue spheres. **d** Average interquartile ranges (IQRs) of distances from spots marking P-body/SG-volumes to their merged geometrical centers. The Q1 and Q3 average values from 10 analyzed condensate assemblies are represented as the lower side and the upper side of the boxes, respectively. The SDs of the Q1 average values are represented as lower bars, and the SDs of the Q3 average values are represented as upper bars (Q1 + Q3 means, SD, *n* = 10 condensate assemblies; ns ≥0.05, **P < 0.01, ****P < 0.0001, two-tailed unpaired Student’s *t* test). Source data are provided as a Source Data file.

Confocal microscopy showed that virtually all RBP47b foci colocalized with SMG7 speckles, whereas no RBP47b signal was detected at a subset of small SMG7 foci (Fig. 5g and Supplementary Fig. 6g–i). However, super-resolution imaging revealed a more nuanced organization: RBP47b did not substantially overlap with SMG7, but instead formed condensates surrounding a more compact SMG7 core (Figs. 5h and 6a). A similar spatial arrangement was also observed between RBP47b and another P-body marker, DCP1 (Fig. 5i, j).

In these lines, RBP47b is expressed in meiotic cells under the control of the strong constitutive *RPS5a* promoter. To rule out the possibility that RBP47b condensation in meiocytes is an artifact of overexpression, we validated this observation using two additional SG marker lines, *eIF4G:YFP* and *UBP1b:YFP*, each expressed from their native promoters (Supplementary Fig. 7). UBP1b, together with RBP47b, is the closest homolog of the mammalian canonical SG protein TIA-1^46^, and localizes to SGs in *Arabidopsis*^24,47^. Both eIF4G-YFP and UBP1b-YFP formed distinct cytoplasmic foci in meiocytes under standard growth conditions, and their abundance markedly increased upon heat shock (Supplementary Fig. 7a, b). These foci, also visible during live imaging of anthers (Supplementary Movie 1), colocalized with SMG7 bodies, but as observed for RBP47b, surrounded the SMG7 core without signal overlap (Supplementary Fig. 7c–f and Fig. 6a).

P-bodies and SGs often associate upon stress induction in somatic cells; however, their arrangement in meiocytes appeared distinct, resembling a core–shell structure. To examine this in more detail, we compared the geometry of heat-induced P-body and SG assemblies in root cells with those observed in meiocytes. We considered two scenarios: SGs and P-bodies either being adjacent to each other or forming a core–shell configuration (Fig. 6b). In the former scenario, spots occupying the volume of individual condensates should, on average, have a similar distance from the geometrical center of the merged condensate volume. In the latter case, spots representing the core should, on average, be closer to the geometrical center than spots representing more distant shell condensates (Fig. 6b). Analysis of 3D-segmented condensates from root cells showed that the volumes of SGs and associated P-bodes are in an approximately similar distance from their merged geometrical center, consistent with an adjacent arrangement (Fig. 6c, d). In contrast, in meocytes, the P-body volume was closer to the geometrical center of the merged condensates than the SG volume, consistent with a core–shell organization (Fig. 6c, d).

In conclusion, these localization experiments demonstrate that *Arabidopsis* meiocytes contain prominent cytoplasmic speckles composed of a P-body core surrounded by a shell of SG components. This spatial organization, together with the observation that SG foci consistently co-occur with P-bodies, further suggests that the SG-shell is nucleated at the surface of P-bodies. We propose the term meiotic bodies (M-bodies) to describe this specialized class of RNP granules.

### SMG7 recruits eIF4A1/2 to the core of M-bodies

**Fig. 7:**
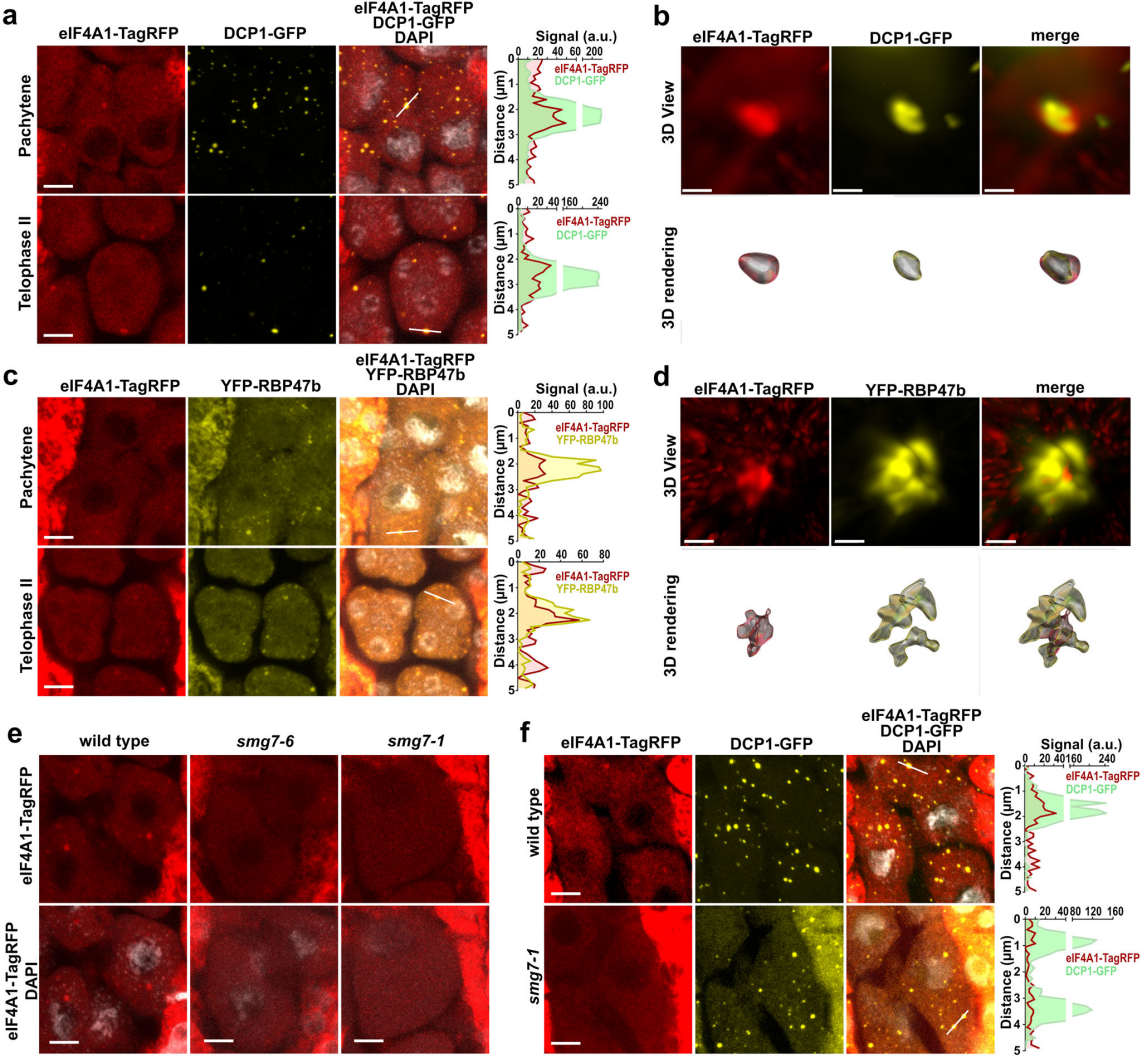
SMG7 recruits eIF4A1 to the core of M-bodies. **a**, **c** Confocal micrographs of *Arabidopsis* meiocytes co-expressing eIF4A1-TagRFP and either DCP1-GFP (**a**) or YFP-RBP47a (**c**). Micrographs depicting pachytene and telophase II meiocytes are shown and are representative of multiple observations (> 20). Diagrams on the right show superimposed intensity profiles of TagRFP and GFP (**a**) or YFP (**b**) measured along the lines indicated in the corresponding micrographs. Each diagram serves as a representative of multiple observations. **b**, **d** Super-resolution micrographs of eIF4A1-TagRFP/DCP1-GFP condensates corresponding to pachytene meiocytes (**b**) and eIF4A1-TagRFP/YFP-RBP47b condensates corresponding to telophase II meiocytes (**d**), visualized by 3D view and 3D rendering using Imaris software. Scale bar = 0.5 μm. **e** Confocal micrographs of *Arabidopsis* pachytene meiocytes of wild-type, *smg7-6*, and *smg7-1* plants expressing eIF4A1-TagRFP, representative of multiple observations (> 20). **f** Confocal micrographs of *Arabidopsis* zygotene meiocytes of wild-type and *smg7-1* mutants co-expressing eIF4A1-TagRFP and DCP1-GFP, representative of multiple observations (> 20). Diagrams on the right show superimposed intensity profiles of TagRFP and GFP signals measured along the lines indicated in the corresponding micrographs. Each diagram serves as a representative of multiple observations. Scale bars, 5 μm. Source data are provided as a Source Data file.

The eIF4A1/2 helicases localize to both P-bodies and SGs in somatic cells under heat shock (Figs. 2a–f and Supplementary Fig. 2a–d, g, h and 3a, d, e). To further refine their location within M-bodies, we performed colocalization analysis of eIF4A1/2 with DCP1 and the SG markers RBP47b, eIF4G, and UBP1b. Similar to SMG7, the eIF4A1/2-TagRFP signal overlapped with DCP1 (Fig. 7a, b and Supplementary Figs. 8e and 9a, b). In contrast, super-resolution microscopy revealed that eIF4A1/2 occupied adjacent, only slightly overlapping territories relative to RBP47b, eIF4G and UBP1, which define the M-body shell (Figs. 7c, d and Supplementary Figs. 8a–e and 9c–h). This finding was unexpected, given that eIF4A1/2 localize to SGs during heat shock and physically interact with eIF4G as part of the eIF4F complex. These data indicate that in meiotic cells, eIF41/2 helicases, along with other P-body components, are restricted to the core of M-bodies.

Several members of the DEAD-box helicase family associate with RNP granules and contain IDRs that facilitate their phase separation into condensates^12,48,49^. However, eIF4A1/2 lack IDRs, and their yeast orthologue has been shown to fail to form phase-separated droplets in vitro^12^, raising the question of how they are recruited into M-bodies. SMG7 has previously been reported to mediate the translocation of UPF1 and TDM1 into P-bodies^23,28^. Therefore, we next investigated whether SMG7 is required for eIF4A1/2 M-body localization. To this end, we crossed *eIF4A1-TagRFP* and *eIF4A2-TagRFP* reporters to the *smg7-1* null mutant as well as to the *smg7-6* hypomorphic allele, which encodes a truncated SMG7 protein lacking the C-terminal IDRs (Fig. 1d) and exhibits reduced partitioning into P-bodies (Supplementary Fig. 1c)^23^. The loading of eIF4A1/2 in cytoplasmic speckles was significantly reduced in *smg7-6* and completely abolished in *smg7-1* mutants (Fig. 7e and Supplementary Fig. 9i).

To exclude the possibility that M-bodies fail to nucleate in the absence of SMG7, we analyzed the colocalization of eIF4A1 with DCP1 and UBP1b in *smg7-1* mutants. This analysis confirmed nucleation of both P-body as well as SG signals in the absence of SMG7 (Fig. 7f and Supplementary Fig. 9j), indicating that M-bodies are formed, but they do not incorporate eIF4A1. Together with our interaction data (Fig. 1), these findings indicate that SMG7, via its 14-3-3 domain, binds eIF4A1/2 and is responsible for recruiting them to the core of M-bodies.

### Downregulation of eIF4A1 enhances TDM1 localization to M-bodies and facilitates meiotic exit

**Fig. 8:**
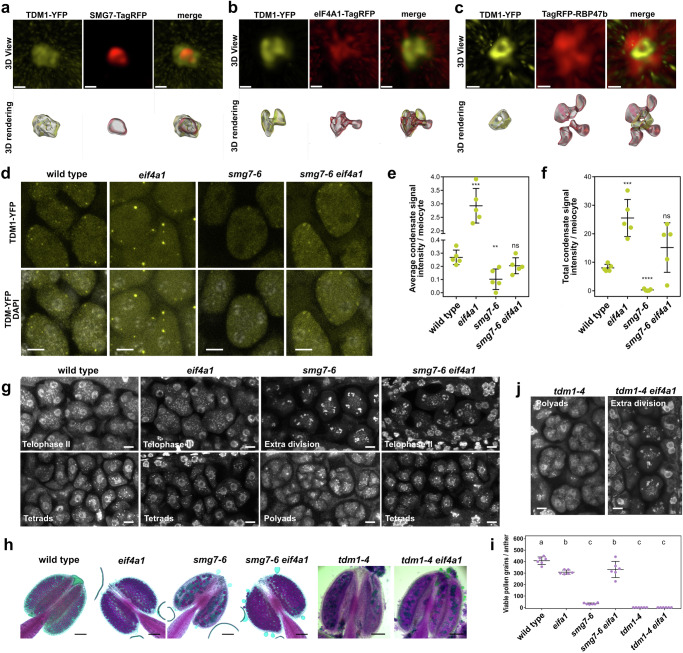
Downregulation of eIF4A1 enhances TDM1 localization to M-bodies and facilitates meiotic exit. **a**–**c** Super-resolution micrographs of indicated protein condensates corresponding to telophase II meiocytes visualized by 3D view and 3D rendering using Imaris software. Scale bar = 0.5 μm. **d** Confocal micrographs of pollen mother cells in telophase II of wild-type, *eif4a1*, *smg7-6*, and *smg7-6 eif4a1* plants expressing TDM1-YFP. Scale bars = 5 μm. **e**, **f** Dot plots indicating average of the signal intensity of condensates (**e**) and total signal intensity (**f**) of all condensates in meiotic cells from **d**; (mean, SD, *n* = 5 telophase II meiocytes; ns ≥ 0.05, **P < 0.01, ***P < 0.001, ****P < 0.0001 two-tailed unpaired Student’s *t* test). **g** Confocal micrographs of meiocytes of wild-type, *eif4a1*, *smg7-6*, and *smg7-6 eif4a1* plants, stained with DAPI, representative of multiple observations (>20). Scale bars = 5 μm. **h** Alexander staining of anthers for pollen viability of wild-type and indicated mutants. Scale bars = 100 μm. **i** Dot plots showing quantification of the viable pollen grains wild-type and indicated mutants (mean, SD, *n* = 6 anthers. One-way ANOVA followed by Tukey’s post hoc test *P* < 0.05). **j** Confocal micrographs of meiocytes of *tdm1-4* and *tdm1-4 eif4a1* plants stained with DAPI, representative of multiple observations (>20). Scale bars = 5 μm. Source data are provided as a Source Data file.

We previously showed that M-bodies play a crucial role in the termination of meiosis and the cell fate transition to post-meiotic microspore development. A key step in this transition is the SMG7-mediated recruitment of TDM1 into P-bodies during meiosis II^23^. In telophase II, TDM1 co-localizes with SMG7, RBP47b and eIF4A1 as observed by confocal microscopy, confirming its presence in M-bodies (Supplementary Fig. 10a–c). To determine the precise localization of TDM1 within the M-body, we performed super-resolution microscopy. This analysis revealed that TDM1-YFP overlaps extensively with SMG7 and eIF4A1/2 in the M-body core, and its overlap with TagRFP-RBP47b, which marks the M-body shell, is substantially smaller (Fig. 8a–c and Supplementary Fig. 10d).

Our data indicate that eIF4A1/2 influences the size of M-bodies, as assessed by SMG7 signal intensity (Fig. 4). Since SMG7 recruits TDM1 into M-bodies, we next asked whether eIF4A1/2 also affects the partitioning of TDM1 into these structures. To test this, we introduced the TDM1-YFP marker into the *eif4a1* mutant background and analyzed TDM1 localization in M-bodies. The absence of eIF4A1 led to a substantial increase in TDM1 signal within M-bodies (Fig. 8d–f), an effect that appears even more pronounced than the SMG7 signal increase in the same mutant background (Fig. 4d–j). The recruitment of TDM1 to M-bodies is severely impaired in *smg7-6* mutants (Fig. 8d–f) due to inefficient M-body partitioning of the C-terminally truncated SMG7 protein (Supplementary Fig. 1c)^23^. Importantly, TDM1 localization to M-bodies is restored in *smg7-6 eif4a1* double mutants (Fig. 8d–f), suggesting that eIF4A1 antagonizes the recruitment or retention of TDM1 in M-bodies.

*Arabidopsis smg7-6* mutants fail to terminate meiosis due to inefficient recruitment of TDM1 into M-bodies^23^. After the formation of haploid nuclei in telophase II, *smg7-6* meiocytes attempt additional rounds of chromosome segregation, leading to unequal chromosome distribution, the formation of polyads instead of tetrads, and a reduction in viable microspores and pollen^23,50,51^. Since eIF4A1 deficiency restores TDM1 recruitment to M-bodies in *smg7-6* mutants (Fig. 8d–f), we examined whether it also restores normal meiotic progression and pollen formation. The *eif4a1* and *eif4a2* mutations alone have no pronounced effect on fertility, and meiosis appears largely normal, except for an increased incidence of chromosome bridges during anaphase I (Supplementary Fig. 11). We also observed a reduced number of meiocytes in *eif4a1* anthers, which correlates with a slightly reduced pollen count (Fig. 8i and Supplementary Fig. 11c). In contrast, *smg7-6* mutants display extra meiotic divisions, polyad formation, and reduced pollen production, but these defects are rescued in *smg7-6 eif4a1* double mutants, which form regular tetrads with four haploid nuclei and display restored pollen counts (Fig. 8g–i).

TDM1 localized to M-bodies promotes meiotic exit by sequestering eIFiso4G2 and inhibiting translation^23^. Since eIF4A1 is a translation initiation factor, it is possible that the *eif4a1* mutation rescues the *smg7-6* phenotype directly by reducing translation, independently of TDM1 localization. To test this, we analyzed meiosis and fertility in *eif4a1 tdm1* double mutants. Like *smg7-6*, *tdm1* mutants exhibit extra meiotic divisions and form polyads, leading to pollen abortion^23,52^. However, this phenotype was not rescued in *eif4a1 tdm1* double mutants, which remained infertile (Fig. 8h, i). These results indicate that eIF4A1 deficiency restores fertility in *smg7-6* mutants by enhancing TDM1 retention in M-bodies, rather than through a general impairment of translation. In conclusion, the findings of this study demonstrate that the eIF4A-mediated remodeling of M-bodies impacts meiotic progression and plant reproduction, thereby underscoring the physiological significance of the degree of condensation of RNP granules.

## Discussion

SMG7 is a prominent component of P-bodies, but the functional significance of this localization remains unclear. SMG7 is primarily known for its role in NMD where it promotes RNA degradation following target recognition in both animals and plants^53–55^. It binds to phosphorylated UPF1 through its 14-3-3 domain, but the molecular events that occur downstream of this interaction are less well understood. The ability of SMG7 to translocate UPF1 into P-bodies initially led to the hypothesis that P-bodies serve as sites for NMD-targeted RNA degradation^28,29,56^. However, subsequent studies demonstrating that NMD can occur independently of P-bodies have challenged this model^57,58^.

In our previous work, we described an NMD-independent function of SMG7 in meiotic exit. This mechanism relies on the interaction between SMG7 and TDM1, which similar to UPF1 occurs through the 14-3-3 phosphoserine-binding pocket, and the ability of SMG7 to translocate TDM1 into the P-body core of M-bodies (Fig. 8)^23^. Importantly, *smg7-6* mutants, in which SMG7 retains the ability to bind TDM1 but has reduced capacity to localize into P-bodies, exhibit a phenotype similar to *tdm1* null mutants. These findings indicate that the key function of SMG7 in meiotic exit is to transport or sequester TDM1 into M-bodies.

Here we identified eIF4A1/2 as additional SMG7 interactors that bind via its 14-3-3 phosphoserine-binding pocket (Fig. 1). Our data further show that SMG7 is necessary for the localization of eIF4A1/2 to the P-body core of M-bodies (Fig. 7e, f and Supplementary Fig. 9i). As with TDM1, C-terminal truncation of SMG7 does not disrupt its interaction with eIF4A1/2, but substantially reduces their recruitment to M-bodies, highlighting the importance of the disordered C-terminal region for P-body localization. This dual domain topology of SMG7, combining a structured 14-3-3-like binding domain and a flexible IDR, provides SMG7 with the capacity to selectively recruit client proteins to modulate the composition and dynamics of P-bodies. In addition to IDRs, the partitioning of SMG7 into P-bodies also requires its structured 14-3-3 domain (Supplementary Fig. 1), although it is independent of the phosphoserine-binding pocket^23^. This observation suggests that the 14-3-3 domain contributes to both substrate binding and P-body association through distinct interactions. Taken together, these observations define a novel role for SMG7 as an adapter or scaffold protein that facilitates the recruitment or retention of specific client proteins in P-bodies.

The eIF4A1/2 helicases are core factors of the translation initiation complex eIF4F. Recently, eIF4A has been proposed to act as an RNA chaperone that limits condensation of SGs in human cells, independently of its function in translation initiation^6^. We found that inactivation of either of the *Arabidopsis* eIF4A isoforms leads to increased size of SGs in root cells (Fig. 3), suggesting a conserved role of eIF4A in SG homeostasis. In contrast to human cells, where eIF4A is present in excess over the other eIF4F subunits^6^, quantitative proteomics across different *Arabidopsis* tissues shows that eIF4A1/2 are roughly equimolar to eIF4G and its isoforms eIFiso4G1/2^59^. Thus, despite genetic redundancy^35^, loss of a single eIF4A ortholog can cause insufficiency and a profound effect on SG formation.

We also observed that eIF4A1/2 are constitutively present in P-bodies, a finding not previously reported. Although the cap-binding protein eIF4E associates with both SGs and P-bodies, other eIF4F components are typically restricted to SGs^40,43,60^. Several lines of evidence suggest that eIF4A1/2 regulate P-body dynamics as RNA chaperones. First, eIF4A1/2 localization does not coincide with that of eIF4G (Fig. 2 and Supplementary Figs. 2 and 3). While both colocalize in SGs during heat stress, eIF4G remains confined to SGs, whereas eIF4A1/2 are also present in adjacent P-bodies. This distinct compartmentalization is even more pronounced in M-bodies, where eIF4G localizes to the shell, while eIF4A1/2 are restricted to the P-body-like core (Supplementary Figs. 7, 8, and 9), indicating an eIF4F-independent function of eIF4A1/2.

Second, *eif4a* mutants show enlarged P-bodies in roots and increased size of the P-body core in M-bodies, consistent with a role in counteracting the RNP condensation. As reported in human cells^6^, eIF4A may limit RNP condensation by restricting intermolecular RNA–RNA interactions. However, we cannot exclude alternative mechanisms, such as decreasing binding multivalency by preventing the association of other RNA-binding proteins. Finally, *eif4a1* mutants exhibit enhanced partitioning of TDM1 into M-bodies, which can rescue meiotic exit in the hypomorphic *smg7-6* background (Fig. 8). This could reflect stronger binding of TDM1 to a P-body constituent such as SMG7, or increased retention of RNPs subassemblies within P-bodies. The presence of larger M-bodies in *eif4a* mutants (Fig. 4) supports the latter, although decreased competition with eIF4A1/2 for SMG7 binding may also contribute to increased TDM1 recruitment.

**Fig. 9:**
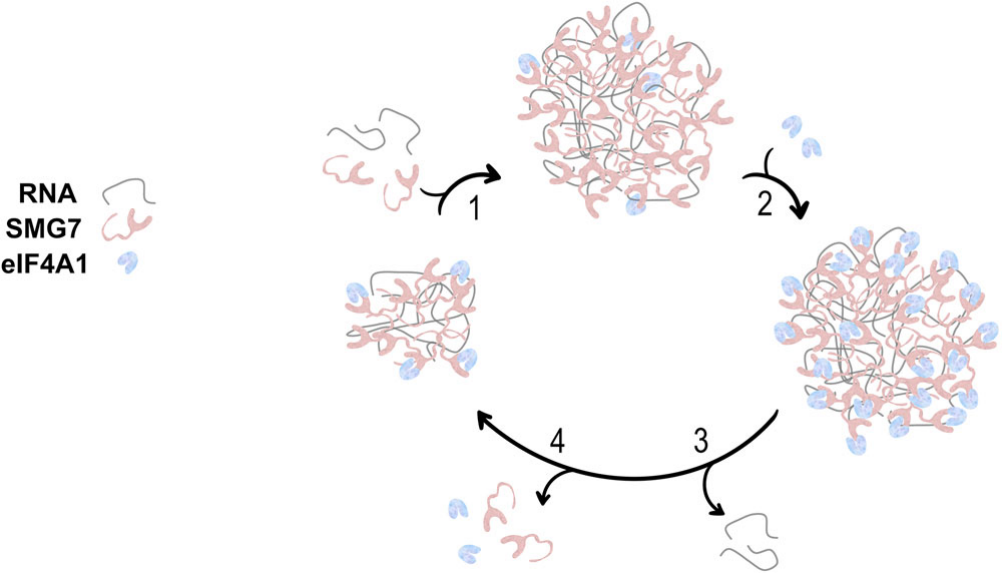
Model of the homeostatic system constituted by SMG7 and eIF4A to control P-bodies condensation. The interplay between SMG7 and eIF4A1/2 may constitute a negative feedback-loop mechanism controlling the degree of P-body condensation. An increased size of P-bodies attracts more SMG7 (1), which in turn recruits more eIF4A1/2 via direct interaction (2). eIF4A1/2 can interfere with the interactions between RNA molecules (3), reducing the degree of condensation and affecting the partitioning of P-body components, including SMG7 and eIF4A (4). Arrow by James Smith from Noun Project (CC BY 3.0).

Given that SMG7 recruits eIF4A1/2 to the P-body core of M-bodies, and that eIF4A1/2, in turn, limit P-body condensation and thereby restrict further partitioning of SMG7, the SMG7-eIF4A interaction module may constitute a negative feedback loop that maintains P-body size homeostasis. In this model (Fig. 9), an increase in P-body size would lead to greater SMG7 accumulation, which would recruit more eIF4A. The resulting increase in eIF4A activity would counteract further condensation, thereby limiting P-body growth. Our data indicate that the SMG7-eIF4A interaction is influenced by eIF4A phosphorylation (Fig. 1), and plant eIF4As have been reported to be phosphorylated in response to hypoxia and heat stress^61,62^. Thus, the SMG7-eIF4A module may dynamically adjust the extent of P-body condensation in response to cellular conditions, thereby affecting RNA processing and gene expression under varying physiological stimuli. Other P-body-associated DEAD-box helicases of the DDX family are likely to contribute to this process, as they have been implicated in P-body assembly and RNA turnover^8,12,48^.

While P-bodies are generally a constitutive feature of plant cells, they are dynamic structures that vary in size and composition depending on the cellular and environmental context^24,63,64^. Here, we show that pollen mother cells contain a distinct type of RNP granules, termed M-bodies, consisting of a P-body core, which is larger than P-bodies in somatic cells, and a shell enriched in SG-specific proteins (Supplementary Fig. 10e). The partitioning of eIF4A1/2 into M-bodies is also more pronounced than their localization to P-bodies in root cells. This may be partly due to the regulation of eIF4A by cyclin-dependent kinase 1 (CDKA;1), which phosphorylates eIF4A1 and renders it translationally inactive^65^. Since CDKA;1 is the key driver of meiotic progression in *Arabidopsis*^66,67^, its activity may increase the pool of translationally inactive eIF4A1 available for SMG7 interaction and recruitment to M-bodies.

In our previous work, we showed that M-bodies play an important role in meiotic exit. They change their composition by recruiting TDM1 during meiosis II, which interacts with and temporarily sequesters eIFisoG2. This is thought to halt translation and promote meiotic exit^23^. Here, we show that the eIF4A1/2 contributes to this M-body remodeling by regulating TDM1 recruitment (Fig. 8). Cytoplasmic condensates, which are presumably M-bodies, have recently also been implicated in ensuring segregation of meiotic chromosomes under heat stress through a mechanism that involves temperature-induced sequestration of meiotic cyclin TAM^68^. Notably, the colocalization of SG and P-body markers has also been observed in rice, suggesting that M-bodies represent a conserved feature of plant meiosis^69^. In rice, these condensates sequester MEL2, an RNA-binding protein required for early meiotic progression, further underscoring the importance of M-bodies in regulating meiotic events across plant species.

M-bodies may also function in the storage and translational repression of mRNAs transcribed early in meiosis, but destined for expression only at later stages or post-meiotically. Similar mechanisms have been described in yeast and human oocytes, where late meiotic and post-meiotic transcripts are sequestered in cytoplasmic RNP condensates nucleated by dedicated RNA-binding proteins^70,71^. Single-cell transcriptomic studies in maize suggest that major transcriptional changes occur during early meiosis, and some of the transcripts persist into post-meiotic pollen development^72,73^, implying a need for their translational repression and storage. The structure of M-bodies is reminiscent of the P-body and SG associations formed during heat shock (Fig. 2), and may similarly reflect a high concentration of translationally repressed mRNA in pollen mother cells. Indeed, we have recently identified an RNA-binding protein that associates with M-bodies and transiently represses the translation of several meiotic transcripts, presumably through their sequestration in M-bodies^74^. In this context, the partitioning of eIF4A into M-bodies could influence the dynamics and translatability of stored mRNAs, similar to the role described for the DDX3X helicase in G3BP1 in vitro condensates^11^.

The discovery of M-bodies as distinct RNP granules with both P-body and SG-like features highlights the complexity of RNA regulation in reproductive cells and suggests that post-transcriptional control via biomolecular condensates plays a central role in plant meiosis. These insights open new avenues for investigating how cells spatially and temporally coordinate gene expression through dynamic condensate architecture.

## Methods

### Plant material and growth conditions

*Arabidopsis thaliana* (ecotype Col-0), mutant lines, and transgenic lines were grown on soil in growth chambers at 21 °C at 50–60% of humidity under 16/8 h light/dark cycles. Roots were analyzed from 4-day-old seedlings grown in agar plates (0.8% w/v, pH 5.8) supplemented with half-strength Murashige and Skoog medium (½ MS). Heat shock was applied to roots by immersing sealed plates in a water bath tempered at 39 °C for 30 min and rapidly processed for fixation. Heat shock was applied to meiotic anthers by immersing the whole inflorescence in liquid ½ MS medium supplemented with 5% sucrose in 1.5-ml tube, and incubation in a heat block at 39 °C for 20 min.

The following *Arabidopsis* mutant lines were used in this study: *smg7-1*^31^ *smg7-6*,^32^, *tdm1-4*^50^, *eif4a1* and *eif4a2*^35^. The mutant line *ubp1b-1* was obtained from NASC (Gabi-Kat GK-262E01-014951) and the primers UBP1b.BamHI.V2.F and UBP1b.6.R (Supplementary Table 2) were used for PCR-genotyping. The following reporter *Arabidopsis* lines were used: *SMG7:MYC*^32,34^, *eIF4G:YFP*^23^, *SMG7:TagRFP*^23^, and *TDM1:YFP*^23^. These reporter lines were generated in this study: *eIF4A1-YFP*, *eIF4A2:YFP*, *eIF4A1:TagRFP*, *eIF4A2:TagRFP*, *YFP:RBP47b*, *TagRFP:RBP47b*, *UBP1b:YFP*, *UBP1b:TagRFP* and *DCP1:G3GFP*.

### Microbial strains and growth conditions

Bacterial cultures in liquid LB media were grown in a constant-temperature incubator with shaking at 200 rpm. Bacterial cultures in solid LB media (1.5% w/v agar plates) were grown in plates in a constant-temperature incubator without shaking.

*Escherichia coli* strain DH5α was cultured in LB medium with appropriate antibiotics at 37 °C.

*Agrobacterium tumefaciens* strain GV3101 was cultivated at 28 °C on LB medium with appropriate antibiotics.

All microbe strains were preserved in glycerol stocks at –80 °C.

### Plasmid construction and generation of transgenic *Arabidopsis* lines

Primer sequences for plasmid construction are listed in Supplementary Table 2. The constructs described in the following section were generated using the Gateway® cloning technology (Invitrogen) and the destination vectors from the pGWB series^75^. The genomic regions containing the putative promoters and ORFs without stop codons of *eIF4A1*, *eIF4A2*, and *DCP1* were amplified with the primers eIF4A1.Prom.F and eif4A1.nostop.r, eif4A2.Promoter.Topo.F and eiF4A2.nostop.r, DCP1_FP and DCP_RP. The fragments were introduced into pENTR™/D-TOPO using blunt-end TOPO® Cloning reactions, and after LR recombination into pGWB640, pGWB659 or pGWB650, the final constructs pGWB640-eIF4A1, pGWB659-eIF4A1, pGWB640-eIF4A2, pGWB659- eIF4A2, and pGWB650-DCP1 were generated to create the lines *eIF4A1:YFP*, *eIF4A2:YFP*, *eIF4A1:TagRFP*, *eIF4A2:TagRFP* and *DCP1:G3GFP*, respectively. These lines were generated by transforming the corresponding mutant lines, or wild-type plants in the case of *DCP1:G3GFP*, and using *Agrobacterium tumefaciens* and the floral dip method.

The genomic region containing the putative promoter and ORF without the stop codon of *UBP1b* was amplified with the primers UBPb1.EcoRI.F.V1 and UBPb1EcoRV.R.V2. The fragment was cloned into the *EcoR*I and *EcoR*V sites of pENTR™11, and LR-recombined into pGWB640 and pGWB659 to obtain the constructs pGWB640-UBP1b and pGWB659-UBP1b, respectively. The constructs pGWB640-UBP1b and pGWB659-UBP1b were used to generate the lines *UBP1b:YFP* and *UBP1b:TagRFP*, respectively, by transforming the mutant line *ubp1b-1* and using *A. tumefaciens* and the floral dip method.

The genomic region containing the ATG and the stop codons of RBP47b was amplified with the primers RBP47b_cacc_topo_F_V2 and RBP47b_stop_R. The fragment was introduced into pENTR™/D-TOPO using blunt-end TOPO® Cloning reaction, obtaining the construct pENTR™/D-TOPO-RBP47b. The CaMV35S promoter of the vectors pGWB642 and pGWB661 was replaced by the pRPS5A promoter, generating the vectors pGWB642(RPS5A) and pGWB661(RPS5A), respectively. pENTR™/D-TOPO-RBP47b was LR-recombined into pGWB642(RPS5A) and pGWB661(RPS5A) to obtain the constructs pGWB642(RPS5A)-RBP47b and pGWB661(RPS5A)-RBP47b, used to generate the lines *YFP:RBP47b* and *TagRFP:RBP47b*, respectively, by transforming the wild-type plants using *A. tumefaciens* and the floral dip method.

For the expression of wild-type and mutated versions of SMG7, eIF4A1, eIF4A1and eIF4B1 in *Arabidopsis* protoplasts and *Nicotiana tabacum* leaves, we utilized the previously described constructs pGWB442-SMG7, pGWB442-SMG7^K77E R185E^, pGWB442-SMG7^Δ715-885^, pGWB442-SMG7^Δ917-1010^, and pGWB442-SMG7^Δ702-105923^. The construct pGWB442-SMG7^Δ1-268^ was generated by amplifying the cDNA of *SMG7* with the primers smg7_del_14-3-3_topoATG and smg7.TOPO.STOP.R. The fragment was introduced into pENTR™/D-TOPO using blunt-end TOPO® Cloning reactions following LR recombination into pGWB442. The cDNAs of *eIF4A1*, *eIF4A2*, and *eIF4B1* were amplified with the following pairs of primers in the respective order: eif4a1.topo.f and eif4a1.nostop.r; EIF4A2.topo.F and eiF4A2.nostop.r; EIF4B1.TOPO.F.real and EIF4B1.NOSTOP.R. The cDNAs were transferred to pENTR™/D-TOPO, and LR recombined into pGWB441, obtaining the constructs pGWB441-eIF4A1, pGWB441-eIF4A2, and pGWB441-eIF4B1. To obtain the constructs, The pENTR/D-TOPO-eIF4A1^S146A^, The pENTR/D-TOPO-eIF4A1^T145A S146A^, and The pENTR/D-TOPO-eIF4A1^S146E^, the pENTR/D-TOPO-eIF4A1 vector was mutagenized with site-directed mutagenesis with the following pairs of primers: eifA1.SDM. S146A.F and eifA1.SDM. S146A.R; eifA1.SDM. T145AS146A.F and eifA1.SDM. T145AS146A.R; eifA1.SDM. S146D.F and eifA1.SDM. S146D.R. Then, LR recombination into pGWB441 was performed to generate the constructs pGWB441-eIF4A1^S146A^, pGWB441-eIF4A1^T145A S146A^, and pGWB441-eIF4A1^S146E^.

The following constructs for the BiFC experiments were previously described^23^: pGW-nY-SMG7, pGW-nY-SMG7^K77E R185E^, pGW-nY-SMG7^Δ715-885^, pGW-nY-SMG7^Δ917-1010^, and pGW-nY-SMG7^Δ702-1059^. We also generated the constructs pGW-nY-SMG7^Δ1-268^, pGW-eIF4A1-cY, pGW-eIF4A2-cY, and pGW-eIF4B1-cY by recombining using LR reaction the entry clones pENTR™/D-TOPO-SMG7 ^Δ1-268^, pENTR™/D-TOPO-eIF4A1, pENTR™/D-TOPO-eIF4A2, and pENTR™/D-TOPO-eIF4B1 with the BiFC vectors pnYGW and pGWcY^75^.

### Protein localization and BiFC assay in protoplasts

*Arabidopsis* mesophyll protoplasts were isolated and transfected as described^76^. Briefly, mesophyll protoplasts were isolated from leaves of 4-week-old *Arabidopsis* grown on soil in a growth chamber at 22 °C under 12/12 h light/dark cycles. In total, 20–30 leaves were cut using a razor blade and digested in 15 ml of digestion solution (1% cellulase Onozuka R10 [Duchefa], macerozyme R10 [Duchefa], 0.4 M mannitol, 20 mM KCl, 20 mM MES pH = 5.7), first for 20 min in a vacuum followed by 3 h in the dark at room temperature. The released protoplasts were filtered with a 70 µm mesh, washed twice with W5 medium (154 mM NaCl, 125 mM CaCl_2_, 5 mM KCl, 2 mM MES pH = 5.7), and stored on ice. For transfection, the protoplasts were resuspended in MMg solution (0.4 mM mannitol, 15 mM MgCl_2_, 4 mM MES pH = 5.7) at 3 × 10^5^ cells/ml. 100 µl of protoplasts were mixed with 15 µg of plasmid and 110 µl of PEG solution (4 g PEG 4000, 2.5 ml mannitol 0.8 M, 1 ml CaCl_2_ 1 M, 3 ml H_2_0) and incubated 10 min in the dark at RT. In total, 440 µl of W5 medium was added to the mixture and, after centrifugation, the protoplasts were resuspended and incubated in W5 medium for 16 h in the dark at room temperature. Transfected protoplasts were imaged using a Zeiss LSM780 confocal microscope.

### FRAP

An LSM780 confocal microscope (Zeiss) was used to bleach the regions of interest. In all, a 661 nm laser at 100% intensity was used to bleach the TagRFP signal of 4-day-old seedlings of the SMG7-TagRFP expressing line. Signal recovery was measured by time-lapse microscopy over the course of 180 s. Image processing and quantification were done in Fiji^77^.

### FLIM-FRET assay

Plasmid vectors were transiently expressed in *N. tabacum* (SR1 Petit Havana) leaf epidermal cells by the infiltration procedures as described^78^. Gene silencing in *Nicotiana tabacum* was suppressed by co-infiltrating the p19 protein from tomato bushy stunt virus cloned into pBIN61^78^. Laser scanning confocal imaging microscope Zeiss LSM 780 AxioObserver equipped with external In Tune laser (488–640 nm, <3 nm width, pulsed at 40 MHz, 1.5 mW) C-Apochromat ×63 water objective, NA 1.2 and, the HPM-100-40 Hybrid Detector from Becker and Hickl GmbH was used for FLIM-FRET data acquisition. FLIM analysis was performed using the Simple-Tau 150 N (Compact TCSPC system based on SPC-150N) with DCC-100 detector controller for photon counting. For excitation of YFP and tagRFP, an InTune laser at 490 nm—efficiently matching YFP’s absorption peak while minimizing off-target excitation and phototoxicity— and a 561 nm DPSS laser were used, respectively^79^. The YFP–tagRFP FRET pair was chosen based on adequate spectral overlap between the YFP emission (~ 527–530 nm) and the tagRFP excitation (~ 555 nm), allowing detection of energy transfer through measurable decreases in YFP fluorescence lifetime (https://www.fpbase.org/spectra/). Although this pair exhibits moderate FRET efficiency, FLIM provides the sensitivity required to detect subtle lifetime shifts indicative of close-range molecular interactions. Zen 2.3 light version from Zeiss was used for processing confocal images. SPCM 64 version 9.8 was used to acquire FLIM data, and SPCImage version 7.3 from Becker and Hickl GmbH for data analysis. For each analysis, fluorescence lifetime was collected in a range between 500 and 5000 ps (A: below 500 ps = background noise, chloroplasts and dirt/dust, B: above 5000 ps = unspecific signal caused by i.e., P-body movement, autofluorescent foci/other cellular compartments) using 2D correlation. A multiexponential decay model was used for fitting. Student’s *t* test was done to evaluate the significant difference in the average lifetime between different groups.

### Cytology

Pollen count and viability were determined by Alexander staining as described^80^, and imaged using the transmitted light microscope Zeiss Axioscope.A1(objective 20×/0.5), with the Axiocam 105 camera and the software Visitrone Visiview. Meiosis was assessed by DAPI staining of PMCs in whole anthers as described^37^, and imaged on a Zeiss LSM780 confocal microscope.

### Root sample preparation for protein localization

Protein localization in *Arabidopsis* roots was performed using roots of 4-day-old seedlings. Seedlings were fixed in 4% formaldehyde in FB (1 mM EDTA, 0.1% Triton X-100 in PBS 1× pH 7). The sample was incubated for 1 h under vacuum, followed by three washes with FB. The sample was stained with 1:1000 dilution of SR2200 in PB for 30 min and washed three times with FB. Finally, the sample was placed on a microscope slide with VECTASHIELD® Antifade Mounting Media for further imaging.

### Anther sample preparation for protein localization

Protein localization in *Arabidopsis* anthers was performed as described^50^. Inflorescences were fixed by vacuum incubation with 4% formaldehyde in FB for 15 min, followed by 45 min of incubation without vacuum. The sample was washed three times with FB before dissecting the meiotic floral buds to isolate meiotic anthers, and then they were incubated for 1 h with 5 µg/ml DAPI in FB. Next, the anthers were washed three times with FB, one time with FB at 60 °C for 10 min, one time with FB on ice, and one time with FB at room temperature. Finally, the anthers were placed in a microscope slide with VECTASHIELD® Antifade Mounting Media to be subsequently imaged.

### Laser scanning confocal imaging

Laser scanning confocal imaging was performed in two systems. The Inverted microscope Zeiss Axio Observer.Z1 with confocal unit LSM 780 was used to image protoplasts, roots (quantification), and anthers. For the protoplasts, we used the objective LCI Plan-Neofluar 63×/1.3 1 mm Korr DIC M27. For the roots and anthers, we used the objective C-Apochromat 63×/1.2 W Korr UV-VIR-IR M27. The Inverted microscope Zeiss Axio Observer.7 with confocal unit LSM 880 was used for the colocalization imaging in roots. In this case, we used the objective Plan-Apochromat 63×/1.4 Oil DIC M27.

### Super-resolution imaging

To obtain a resolution beyond the Abbe diffraction limit of the conventional light microscope, we used the motorized inverted microscope ZEISS Elyra 7 with a lattice illumination pattern for 3D structured illumination (Lattice SIM). This system was used to image RNP granules of roots and meiocytes. We used the objective Plan-Apochromat 63×/1.4 Oil DIC M27 and the 2× PCO edge sCMOS camera, 1280 × 1280, pixel size 6.5 μm × 6.5 μm. For excitation, we used 488 nm and 561 nm lasers. The raw images were acquired using a dimension of 1024 × 1024 pixels (pixel size 63 nm) and 9 phases.

### Live-cell imaging

Live-cell imaging of *Arabidopsis* pollen mother cells was performed using Light sheet microscopy as previously described^81^. Anthers were imaged in Light sheet Z.1 microscope (Zeiss) (Objective W Plant-Apochromat 20×/1.0 DIC) in 10 min time increments.

### Image processing

Images obtained by laser scanning confocal imaging were processed for image presentation with the software ZEN (blue and black edition). The 3D segmentation and the signal quantification of RNP granules from roots and meiocytes were performed with the Imaris 10.2.0 microscopy image analysis software, utilizing the volume rendering. The images obtained with the super-resolution microscope ZEISS Elyra 7 were processed in ZEN Black 3.0 SR (Zeiss), utilizing the 3D SIM2 algorithm, with output sampling = 2 and scaled to raw. The software Imaris 10.2.0 was used for 3D segmentation and volume rendering, which was used to quantify the percentage of overlap between the volumes of different components. The built-in spot detection algorithm in Imaris was used to sub-compartmentalize the segmented volumes in estimated diameters of 0.05 µm, and measure their distance from the centroid of the desired segmented volume. The centroids were obtained after unifying 2 associated volumes, and utilizing the spot detection algorithm based on an estimated diameter of 0.6–1.5 µm.

The live imaging movie obtained by Light sheet microscope was processed using ZEN (Zeiss - Light sheet module).

### Western blot analysis

Protein extracts were prepared by grinding leaves, stems, and inflorescences from 35-day-old *SMG7-MYC* line and wild-type control in liquid nitrogen. Whole protein extract was obtained after 15 min incubation at 4 °C with protein extraction buffer (50 mM Tris-HCl pH 7.5, 150 mM NaCl, 0.1% Nonidet P-40, 10% Glycerol, 1 mM PMSF) supplemented with protease inhibitor cocktail (Roche). After 15 min of incubation at 4 °C, the sample was centrifuged at 30,000× *g*. The protein extract contained in the supernatant fraction was quantified, and 45 µg of protein were loaded onto an SDS-PAGE gel. For Western blotting, SMG7-MYC was detected using anti-MYC polyclonal antibody (ab9106, Abcam).

### Immunoprecipitation and mass spectrometry

Leaves, stems, and inflorescences from 35-day-old *SMG7-MYC* line and a wild-type control were ground in liquid nitrogen and processed using the EZview™ Red Anti-c-Myc Affinity Gel (Merck). Briefly, 2 g of ground tissue was mixed with 5 ml of protein extraction buffer (50 mM Tris-HCl pH 7.5, 150 mM NaCl, 0.1% Nonidet P-40, 10% Glycerol, 1 mM PMSF) supplemented with protease inhibitor cocktail (Roche) and PhosStop phosphatase inhibitor cocktail (Roche). After 15 min of incubation at 4 °C, the sample was centrifuged at 30,000× *g*. In total, 10 mg of protein were incubated with 50 µl of affinity gel for 1 h 30 min with rotation. Finally, the sample was centrifuged for 8200× *g* and washed five times with the extraction buffer.

In-gel digestion: The immunoprecipitated proteins were separated using 1D-SDS-PAGE gel electrophoresis (12% gel, one replicate per gel), and whole gel lanes selected for analysis were excised in the form of bands (30 bands per lane). After destaining and washing procedures, each band was washed by 50% ACN/NaHCO_3_ and pure ACN, and the gel pieces were incubated with 125 ng trypsin (sequencing grade; Promega) in 50 mM NaHCO_3_. The digestion was performed for 2 h at 40 °C on a Thermomixer (750 rpm; Eppendorf). Tryptic peptides were extracted into LC-MS vials by 2.5% formic acid (FA) in 50% ACN with the addition of polyethylene glycol (20,000; final concentration 0.001%) and concentrated in a SpeedVac concentrator (Thermo Fisher Scientific).

LC-MS/MS analyses of individual gel band digests were done Ultimate 3000 RSLCnano system connected to an Orbitrap Elite hybrid spectrometer (Thermo Fisher Scientific). Prior to LC separation, tryptic digests were online concentrated and desalted using a trapping column (100 μm × 30 mm) filled with 3.5-μm X-Bridge BEH 130 C18 sorbent (Waters). After washing of trapping column with 0.1% FA, the peptides were eluted (flow 300 nl/min) from the trapping column onto a Acclaim Pepmap100 C18 column (2 µm particles, 75 μm × 250 mm; Thermo Fisher Scientific) by the following gradient program (mobile phase A: 0.1% FA in water; mobile phase B: 80% ACN containing 0.1% FA): the gradient elution started at 1% of mobile phase B and increased from 1% to 56% during the first 50 min (1% in the 1st, 30% in the 30th and 56% in 50th min), then increased linearly to 80% of mobile phase B in the next 5 min and remained at this state for the next 10 min. Equilibration of the trapping column and the column was done prior to sample injection to sample loop. The analytical column outlet was directly connected to the Nanospray Flex Ion Source (Thermo Fisher Scientific).

MS data were acquired in a data-dependent strategy selecting up to the top ten precursors based on precursor abundance in the survey scan (350–2000 *m/z*). The resolution of the survey scan was 60,000 (at *m/z* 400) with a target value of 1 × 10^6 ^ions, one microscan, and maximum injection time of 200 ms. HCD MS/MS spectra (relative fragmentation energy of 32%, resolution 15,000 at *m/z* 400) were acquired with a target value of 50,000 ions with *m/z* range adjusted according to actual precursor mass and charge. The maximum injection time for MS/MS was 500 ms. Dynamic exclusion was enabled for 45 s after one MS/MS spectra acquisition, and early expiration was disabled. The isolation window for MS/MS precursor isolation was set to 2 *m/z*.

For data evaluation, MaxQuant software (v1.6.17.0)^82^ with an in-built Andromeda search engine^83^ was used. Search was done against protein databases of *Arabidopsis thaliana* (27,469 protein sequences, version from 2021-11-17, downloaded from: https://ftp.uniprot.org/pub/databases/uniprot/current_release/knowledgebase/reference_proteomes/Eukaryota/UP000006548/UP000006548_3702.fasta.gz) and cRAP contaminants (112 sequences, version from 2018-11-22, downloaded from http://www.thegpm.org/crap). The following variable modifications were set for the database search: oxidation (M), propionamide (C), and acetylation (Protein N-term). Enzyme specificity was tryptic with two permissible miscleavages. Only peptides and proteins with a false discovery rate threshold under 0.01 were considered. Relative protein abundance was assessed using protein intensities calculated by MaxQuant. Match between runs was enabled and set using the fractions scheme specifying individual bands as different consecutive fractions (1–30) using the same fraction number for the corresponding gel area from the two samples of one replicate but different fraction numbers between replicates.

Intensities of reported proteins were further evaluated using software container environment (https://github.com/OmicsWorkflows/KNIME_docker_vnc; version 4.1.3a). Processing workflow is available upon request and it covered, in short, reverse hits and contaminant protein groups (cRAP) removal, protein group intensities log2 transformation and normalization (loessF). Replicate-wise ratios were calculated using the normalized data and evaluated to filter for a putative protein candidate list. A complete list of proteins within each protein group can be viewed in the supporting material (Supplementary Table 1).

### Protein co-immunoprecipitation and western

Leaves and inflorescences from 35-day-old *SMG7-MYC*, *eIF4A1-YFP*, and *SMG7-MYC eIF4A1-YFP* lines were ground in liquid nitrogen. Whole protein extract was obtained after 15 min incubation at 4 °C with protein extraction buffer (50 mM Tris-HCl pH 7.5, 150 mM NaCl, 0.1% Nonidet P-40, 10% Glycerol, 1 mM PMSF) supplemented with protease inhibitor cocktail (Roche). After 15 min of incubation at 4 °C, the sample was centrifuged at 30,000× *g*. The protein extract contained in the supernatant fraction was quantified, and 2.5 mg of protein were incubated with GFP-Trap® Magnetic Agarose (AB_2631358 ChromoTek) for 3 h 30 min at 4 °C with rotation. After four washes with the extraction buffer, proteins were eluted from the affinity beads by adding 4× Laemmli sample buffer. For Western blotting, proteins were detected using anti-GFP monoclonal antibody (3H9) (AB_10773374 ChromoTek) and anti-MYC polyclonal antibody (ab9106, Abcam).

### Protein structure model building

The Alphafold-3 model of the SMG7-eIF4A1 interaction was downloaded from the Alphafold Server^39^ and visualized and prepared for presentation with UCSF ChimeraX^84^.

### Quantification and statistical analysis

Quantification analysis of condensates was performed using Imaris 10.2.0 software. Counting of viable pollen grains was performed using the Count tool of Adobe Photoshop 13.0 CS6 Extended.

Data analysis and graph constructions were performed using GraphPad Prism 7 software. Significance testing was conducted with Student’s *t* tests with a significance threshold of *P* < 0.05, and one-way ANOVA followed by Tukey’s post hoc test. Further details can be found in the figure legends.

### Reporting summary

Further information on research design is available in the Nature Portfolio Reporting Summary linked to this article.

## Supplementary information

Supplementary Information

Description of Additional Supplementary File

Supplementary Movie 1

Reporting Summary

Transparent Peer Review file

## Source data

Source data

## Data Availability

Mass spectrometry proteomics data were deposited to the ProteomeXchange Consortium via PRIDE^85^ partner repository under dataset identifier PXD061406 and 10.6019/PXD061406. The microscopy data generated in this study have been deposited at the EBI bioimage archive (https://www.ebi.ac.uk/bioimage-archive/) under accession number S-BIAD3172. Source data are provided with this paper.
