# Supplementary material for: SMG7 and eIF4A constitute a homeostatic module controlling P-body condensation and function of meiotic bodies
Source: Nat Commun. 2026 Apr 21;17:5477. doi: 10.1038/s41467-026-72218-w (PMC13284301; doi:10.1038/s41467-026-72218-w)
Supplement: Supplementary file 1 — Supplementary Information [file 41467_2026_72218_MOESM1_ESM.pdf]

Supplementary information

SMG7 and eIF4A constitute a homeostatic module controlling P-body condensation and function of Meiotic bodies

Albert Cairo[1*], Neha Shukla[1], Sofia Kanavorova[1], Jan Skalak[1], Pavlina Mikulkova[1], Anna Vargova[1], David Potesil[1], Zbynek Zdrahal[1], Jan Hejatko[1], Karel Riha[1*]

[1]Central European Institute of Technology, Masaryk University, Brno, Czech Republic

*Corresponding authors: albert.calzada@ceitec.muni.cz ; karel.riha@ceitec.muni.cz

Supplementary Figures 1-10

Supplementary Movie 1

Supplementary Tables 1-2

Supplementary Fig. 1: SMG7 interacts with the RNA helicase eIF4A in P-bodies.

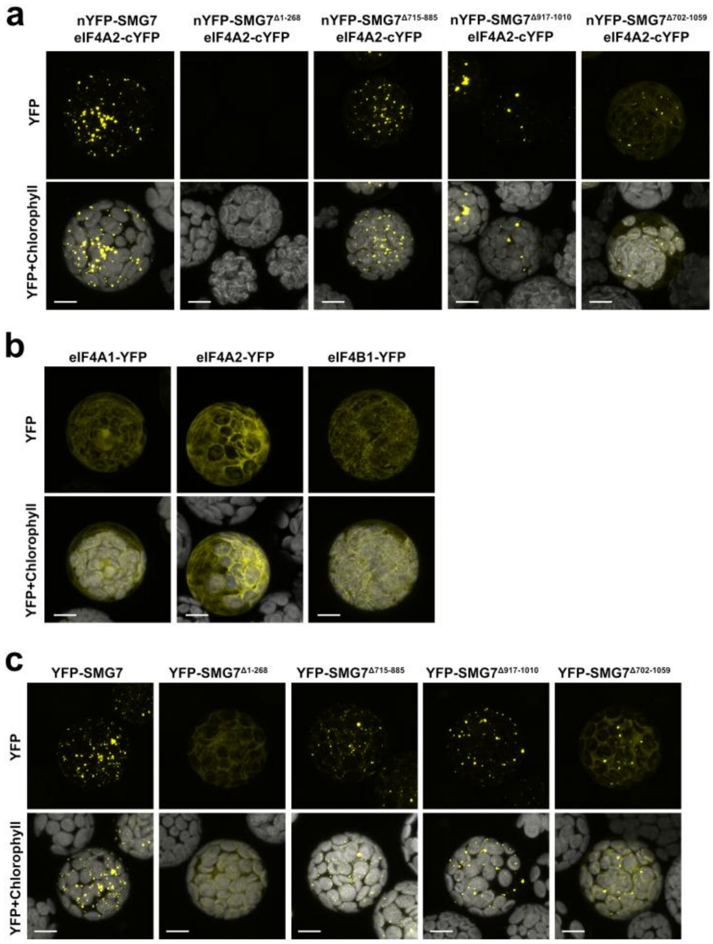

a, BiFC assay in *Arabidopsis* mesophyll protoplasts of eIF4A2 interaction with the specified mutated versions of SMG7. Scale bar, 10 μm. **b**, *Arabidopsis* mesophyll protoplasts transiently transfected with the plasmid pGWB-eIF4A1-YFP, pGWB-eIF4A2-YFP and pGWB-eIF4B1-YFP. Scale bar, 10 μm. **c**, *Arabidopsis* mesophyll protoplasts transiently transfected with the plasmid pGWB-YFP-SMG7 and the specified mutated versions. The micrographs are representative of multiple observations encompassing more than three different experiments. Scale bar, 10 μm.

Supplementary Fig. 2: eIF4A2 colocalize to P-bodies and SGs.

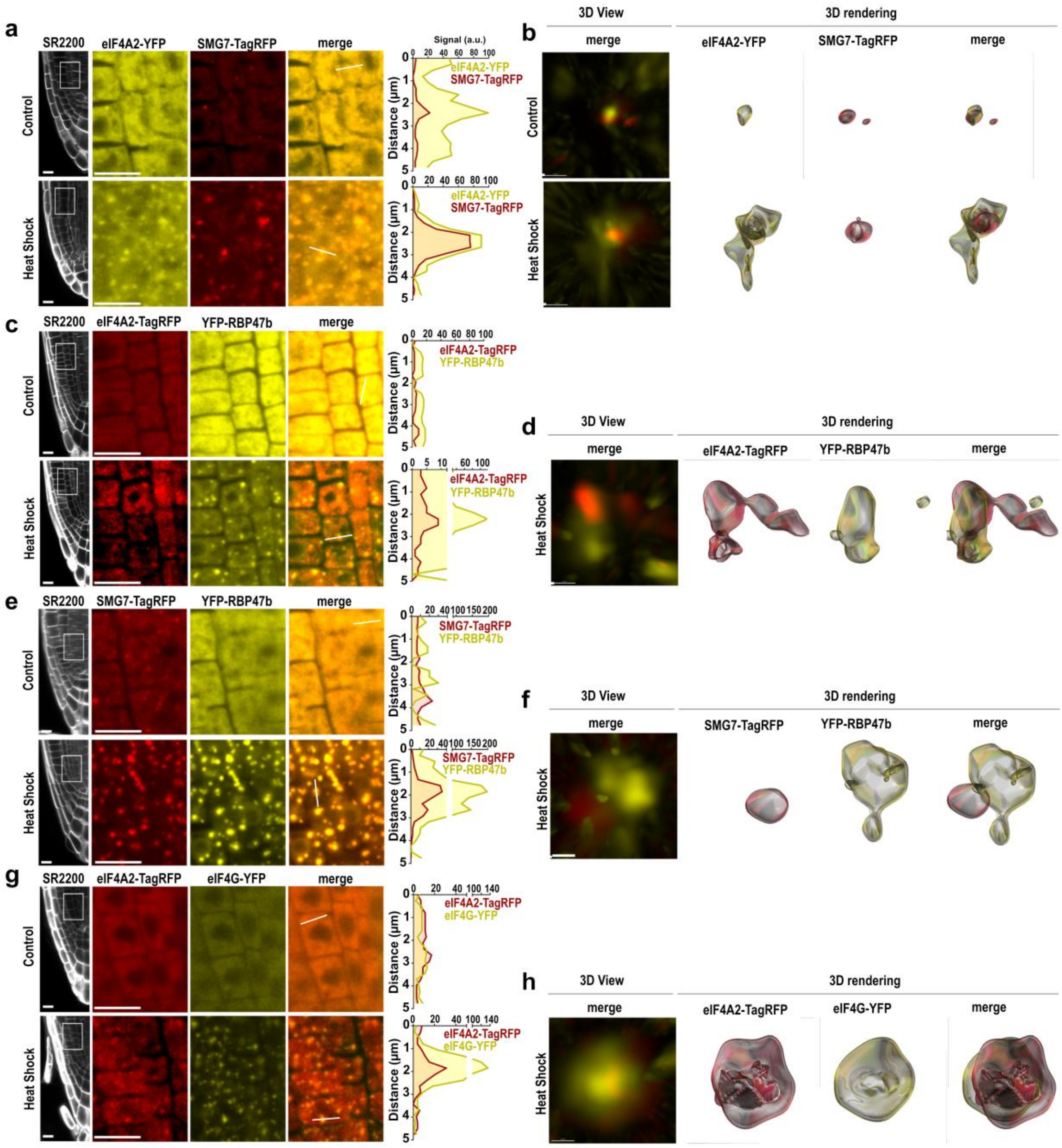

a,**c**,**e**,**g** Confocal micrographs of root cells co-expressing indicated combinations of reporter proteins. Counterstaining with SR2200 dye was used to visualize cell walls. A minimum of three root tips were subjected to analysis, with each micrograph serving as a representative sample. Diagrams on the right show superimposed intensity profiles of YFP and TagRFP signals measured along the lines indicated in the corresponding micrographs. Each diagram serves as a representative of multiple observations. Scale bar = 10 µm. **b**,**d**,**f**,**h** Super-resolution micrographs of indicated protein condensates visualized by 3D view and 3D rendering using Imaris software. Scale bar = 0.5 µm. Source data are provided as a Source Data file.

Supplementary Fig. 3: Quantification of the colocalization of eIF4A1/2 to P-bodies and SGs.

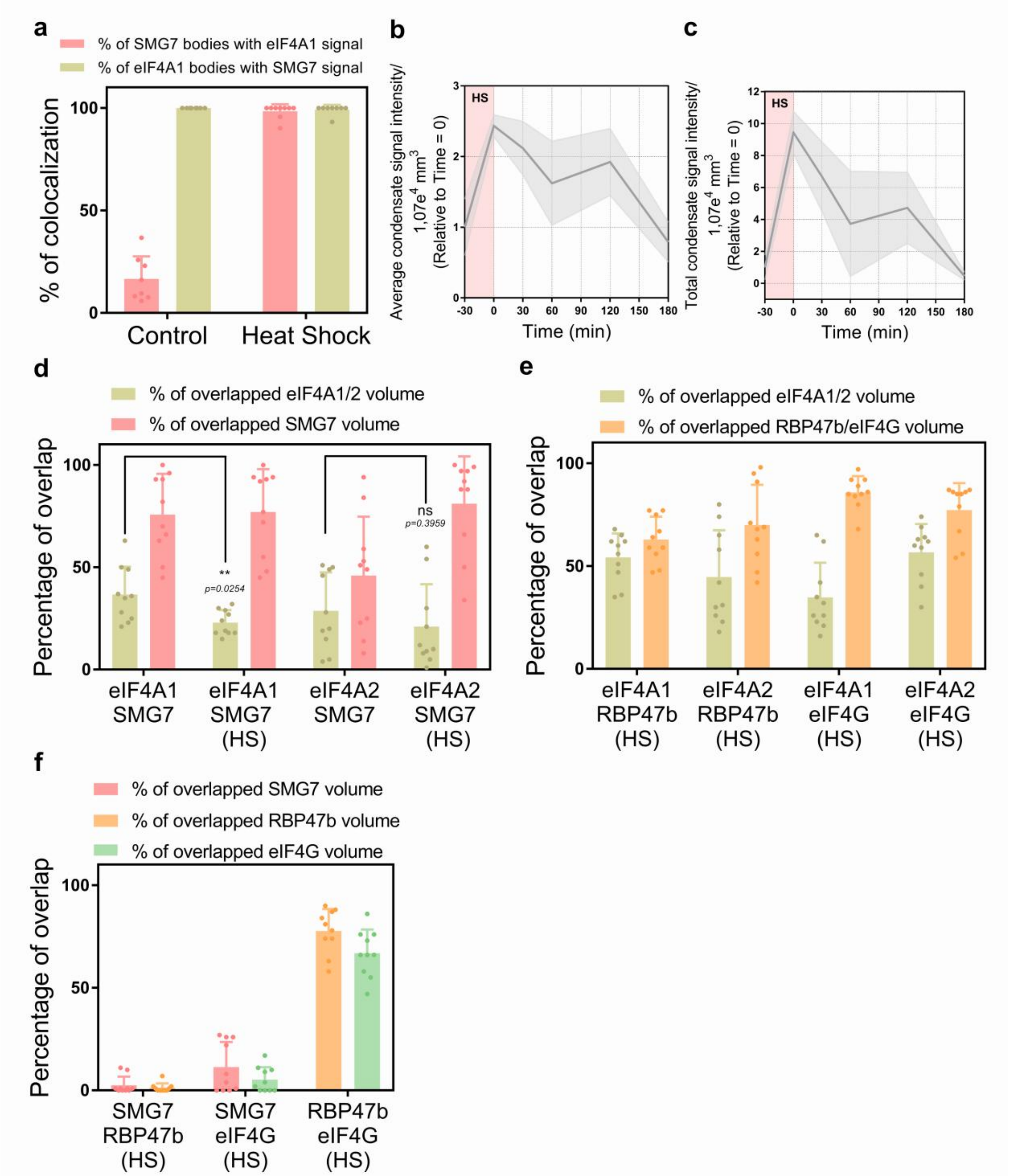

a, Percentage of colocalization between SMG7-TagRFP and eIF4A1-YFP foci observed in confocal micrographs of root tips under control and heat shock conditions. Each data point represents the average of colocalization within a volume of 1.07e4 μm^3 encompassing endodermic and cortical cells (mean, SD, n = 8 root tip volumes). **b**,**c**, Kinetics of P-body condensation during 30 min heat-shock treatment followed by 180 min of recovery time at 21°C. The graphs show the average SMG7-TagRFP signal intensity per condensate (**b**), and the total condensate signal intensity (**c**) in a volume of $1.07e^4$ μm^3 encompassing endodermic and cortical cells (mean, SD, n = 6-10 root tip volumes per time point). **d**,**e**,**f**, Proportions of volume overlaps between condensates of the indicated proteins visualized by super-resolution microscopy (mean, SD, n = 10 associated condensates; two-tailed unpaired Student's t test). Source data are provided as a Source Data file.

Supplementary Fig. 4: eIF4A1 does not affect the dynamics of SMG7 within P-bodies.

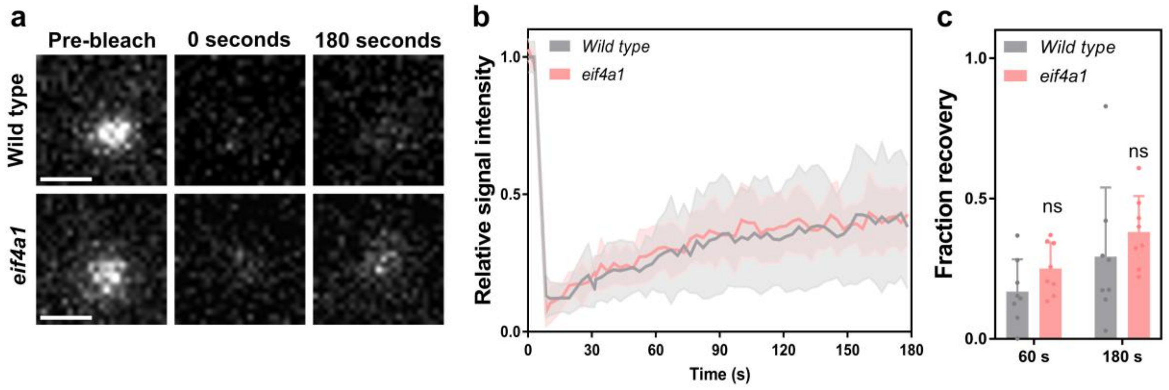

a-**c**, FRAP of SMG7-TagRFP signal in P-bodies of wild type and *eif4a1* root tips. **a**, Confocal micrographs of SMG7-TagRFP condensates before photobleaching and at 0 s or 180 s after photobleaching. Scale bar, 1 μm. **b**, Chart depicting the kinetics of the SMG7-TagRFP signal recovery after photobleaching **c,** Fraction of signal recovered at 60 s and 180s after photobleaching. (mean, SD, n = 8 bleached condensates; ns ≥ 0.05, two-tailed unpaired Student's t test). Source data are provided as a Source Data file.

Supplementary Fig. 5: eIF4A2 localize to meiotic P-bodies.

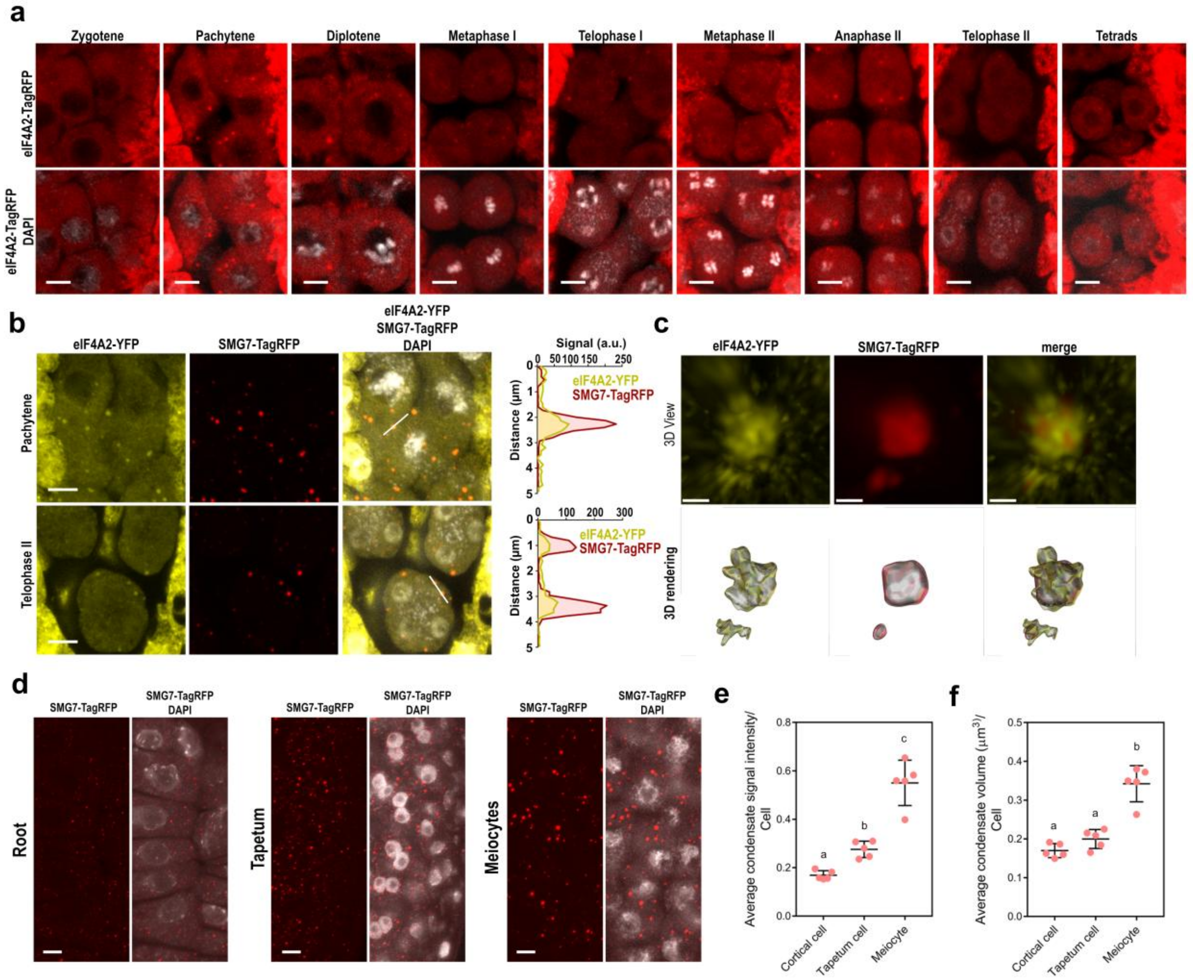

a, Confocal micrographs of *Arabidopsis* anthers showing localization of eIF4A2-TagRFP in the course of meiosis. DNA is counterstained with DAPI. Each micrograph serves as a representative image of multiple observations (>20). Scale bars = 5 µm. **b**, Confocal micrographs of *Arabidopsis* meiocytes co-expressing eIF4A2-TagRFP and SMG7-TagRFP. Micrographs depicting pachytene and telophase II meiocytes are shown, and are representative of multiple observations (>20). Diagrams on the right show superimposed intensity profiles of YFP and TagRFP signals measured along the lines indicated in the corresponding micrographs. Each diagram serves as a representative of multiple observations. Scale bar = 10 µm. **c**, Super-resolution micrographs of indicated protein condensates corresponding to zygotene meiocytes visualized by 3D view and 3D rendering using Imaris software. Scale bar = 0.5 µm. **d**, Confocal micrographs of root cortical cells, tapetum cells and meiocytes of the *SMG7:TagRFP* line. Scale bars, 5 µm. **e**,**f** Dot plots showing average signal intensity per condensate in a cell (**e**) and average of volume per condensate in a cell (**f**) from **d** (mean, SD, n = 5 cells, One-way ANOVA followed by Tukey's post-hoc test $p < 0.05$). Source data are provided as a Source Data file.

Supplementary Fig. 6: Quantification of the eIF4A1, SMG7 and RBP47b foci and their colocalization during meiosis.

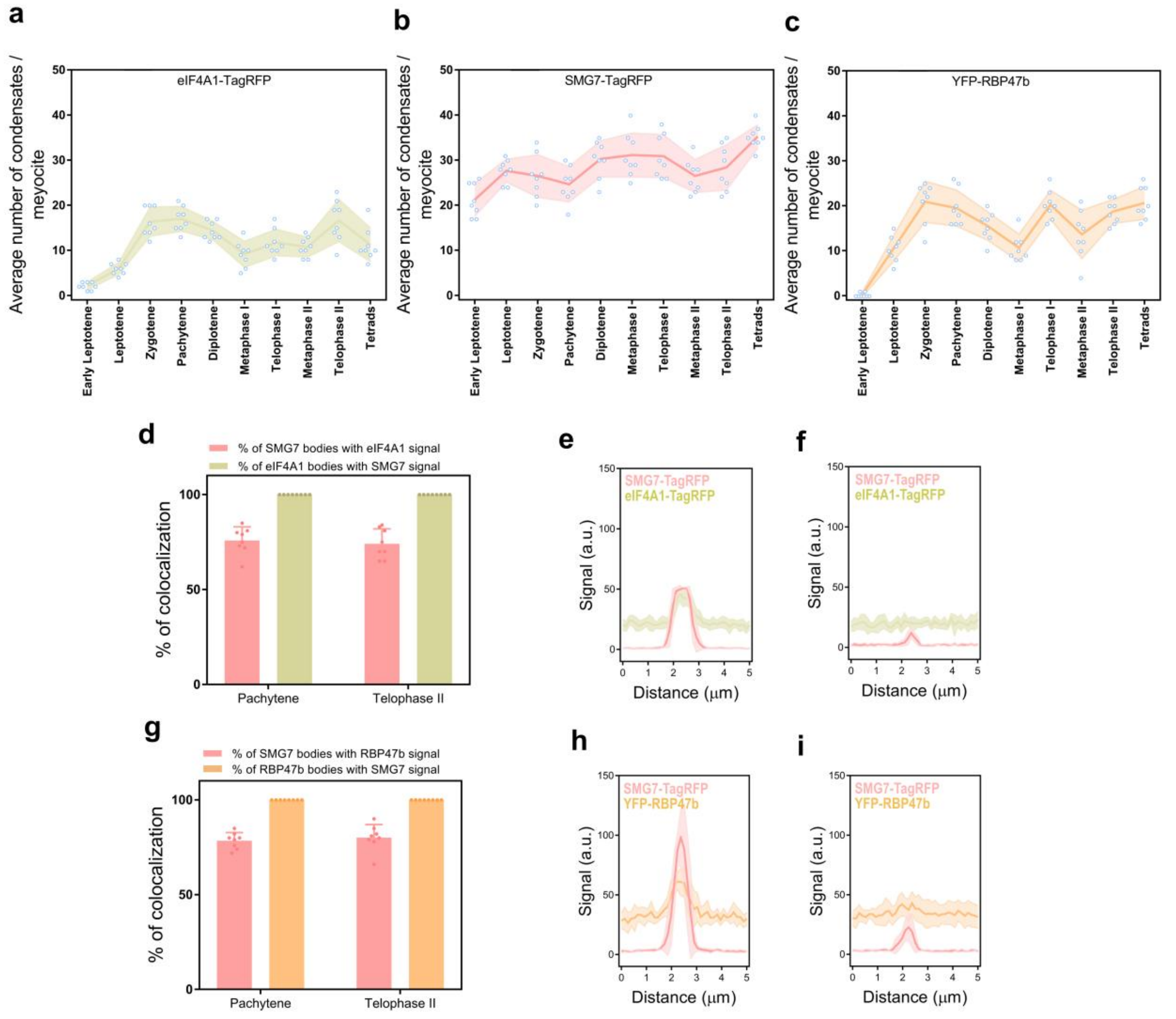

a-**c**, Number of eIF4A1 (**a**), SMG7 (**b**) and YFP-RBP47b (**c**) foci detected by confocal microscopy during meiotic progression (mean, SD, n = 8 meiocytes). **d**, Percentage of colocalization between SMG7 and eIF4A1 foci observed by confocal microscopy in meiocytes at pachytene and telophase II stages (mean, SD, n meiocytes = 8). **e**,**f**, Diagrams showing superimposed intensity profiles of SMG7-TagRFP and eIF4A1-YFP signals measured along representative large (**d**) or small (**e**) SMG7-TagRFP condensates (mean, SD, n = 6 foci). **g**, Percentage of colocalization of SMG7 and RBP47B foci observed in confocal micrographs of meiocytes at pachytene and telophase II stages (mean, SD, n = 8 meiocyes). **h**,**i**, Diagrams showing superimposed intensity profiles of SMG7-TagRFP and YFP-RBP47b signals measured along representative large (**d**) or small (**e**) SMG7-TagRFP condensates. (mean, SD, n = 6 foci). Source data are provided as a Source Data file.

Supplementary Fig. 7: Composite RNP granules consisting of a P-body core and a SG shell form during meiosis.

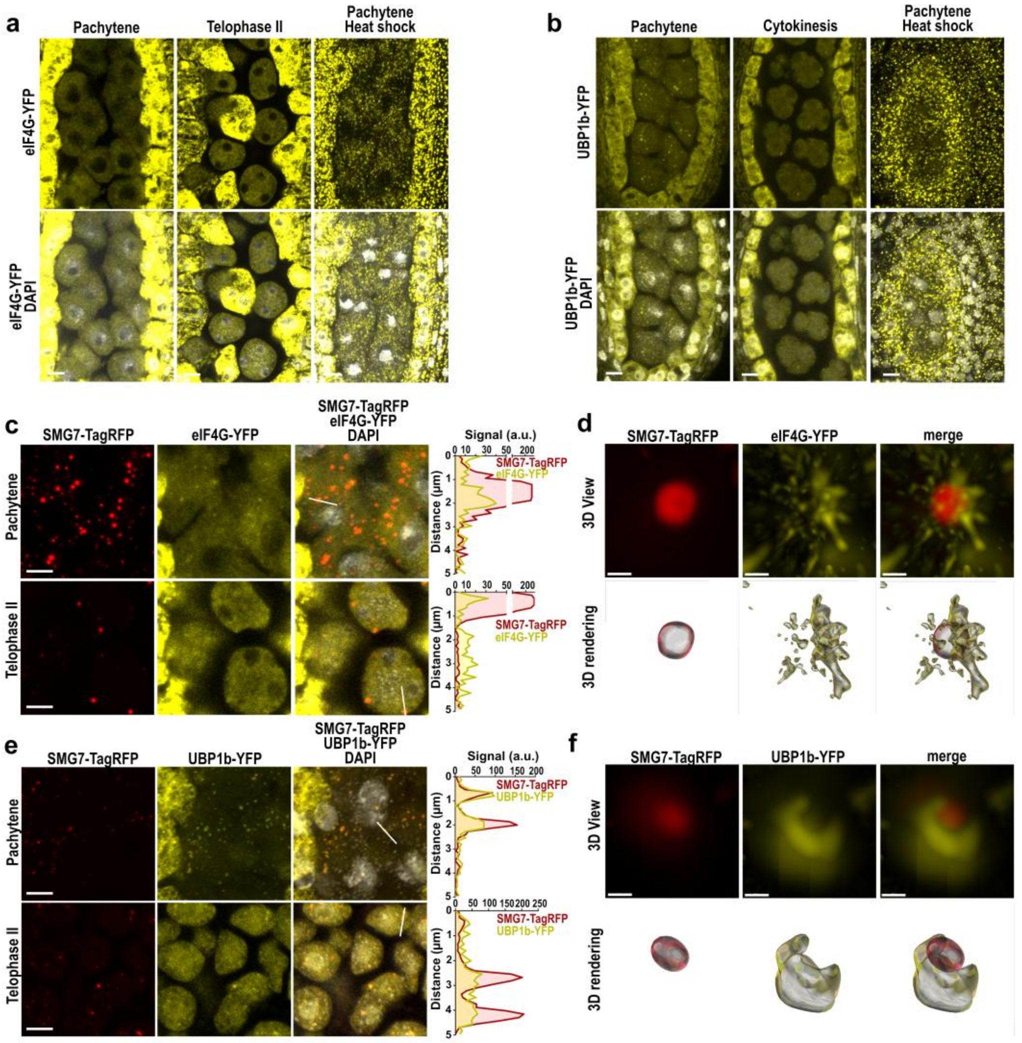

a, Confocal micrographs of anther lobes, showing expression and localization of eIF4G-YFP in meiocytes in pachytene and telophase II and the surrounding tapetum upon heat shock or under control conditions without heat shock application. Shown micrographs are representative of multiple observations (>20). Scale bar = 5 μm. **b**, Confocal micrographs of anther lobes, showing expression and localization of UBP1b-YFP in meiocytes in pachytene and telophase II and the surrounding tapetum upon heat shock or under control conditions without heat shock application. Shown micrographs are representative of multiple observations (>20). Scale bar = 5 μm. **c**, Confocal micrographs of *Arabidopsis* meiocytes co-expressing SMG7-TagRFP and eIF4G-YFP. Micrographs depicting pachytene and telophase II meiocytes are shown, and are representative of multiple observations (>20). Diagrams on the right show superimposed intensity profiles of YFP and TagRFP signals measured along the lines indicated in the corresponding micrographs. Each diagram serves as a representative of multiple observations. Scale bar = 5 μm. **d**, Super-resolution micrographs of SMG7-TagRFP/eIF4G-YFP condensates corresponding to pachytene meiocytes visualized by 3D view and 3D rendering using Imaris software. Scale bar = 0.5 μm. **e**, Confocal micrographs of *Arabidopsis* meiocytes co-expressing SMG7-TagRFP and UBP1b-YFP. Micrographs depicting pachytene and telophase II meiocytes are shown, and are representative of multiple observations (>20). Diagrams on the right show superimposed intensity profiles of YFP and TagRFP signals measured along the lines indicated in the

corresponding micrographs. Each diagram serves as a representative of multiple observations. Scale bar = 5 μm. **f**, Super-resolution micrographs of SMG7-TagRFP/UBP1b-YFP condensates corresponding to pachytene meiocytes visualized by 3D view and 3D rendering using Imaris software. Scale bar = 0.5 μm. Source data are provided as a Source Data file.

Supplementary Fig. 8: eIF4A1/2 are located to the core of M-bodies, surrounded by a shell constituted by SG components.

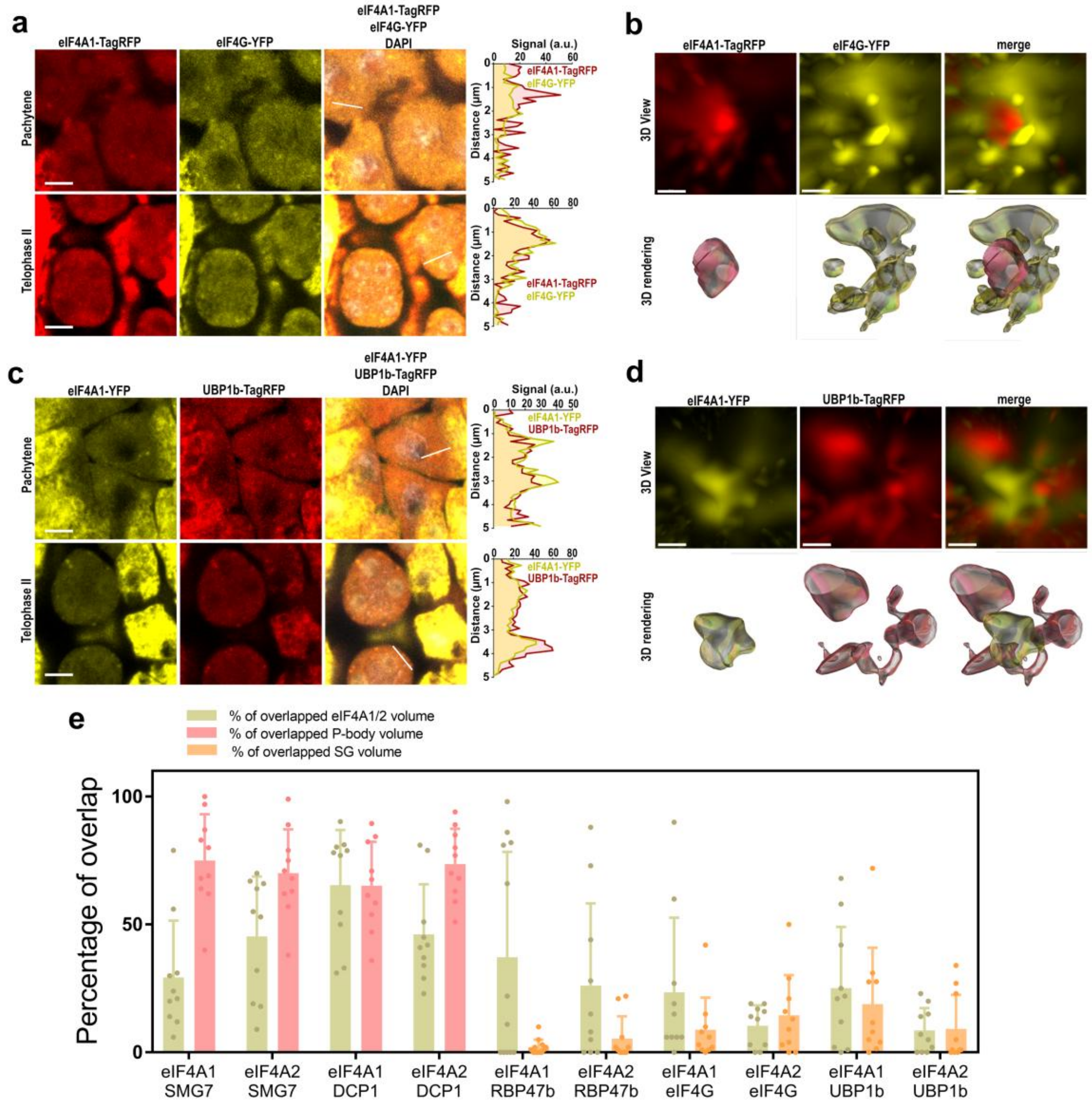

a, Confocal micrographs of *Arabidopsis* meiocytes co-expressing eIF4A1-TagRFP and eIF4G-YFP. Micrographs depicting pachytene and telophase II meiocytes are shown, and are representative of multiple observations (>20). Diagrams on the right show superimposed intensity profiles of GFP and TagRFP signals measured along the lines indicated in the corresponding micrographs. Each diagram serves as a representative of multiple observations. Scale bar = 5 µm. **b**, Super-resolution micrographs of eIF4A1-TagRFP / eIF4G-YFP condensates corresponding to tetrads stage visualized by 3D view and 3D rendering using Imaris software. Scale bar = 0.5 µm. **c**, Confocal micrographs of *Arabidopsis* meiocytes co-expressing eIF4A1-TagRFP and UBP1b-YFP. Micrographs depicting pachytene and telophase II meiocytes are shown, and are representative of multiple observations (>20). Diagrams on the right show superimposed intensity profiles of YFP and TagRFP signals measured along the lines indicated in the corresponding micrographs. Each diagram serves as a representative of multiple observations. Scale bar = 5 µm. **d**, Super-resolution micrographs of eIF4A1-TagRFP / UBP1b-YFP condensates corresponding to telophase II meiocytes visualized by 3D view and 3D rendering using Imaris software. Scale bar = 0.5 µm. **e**, Percentages of volume overlaps between indicated protein condensates in meiocytes from super-resolution microscopy (mean, SD, n = 10 associated condensates). Source data are provided as a Source Data file.

Supplementary Fig. 9: SMG7 recruits eIF4A2 to the core of M-bodies.

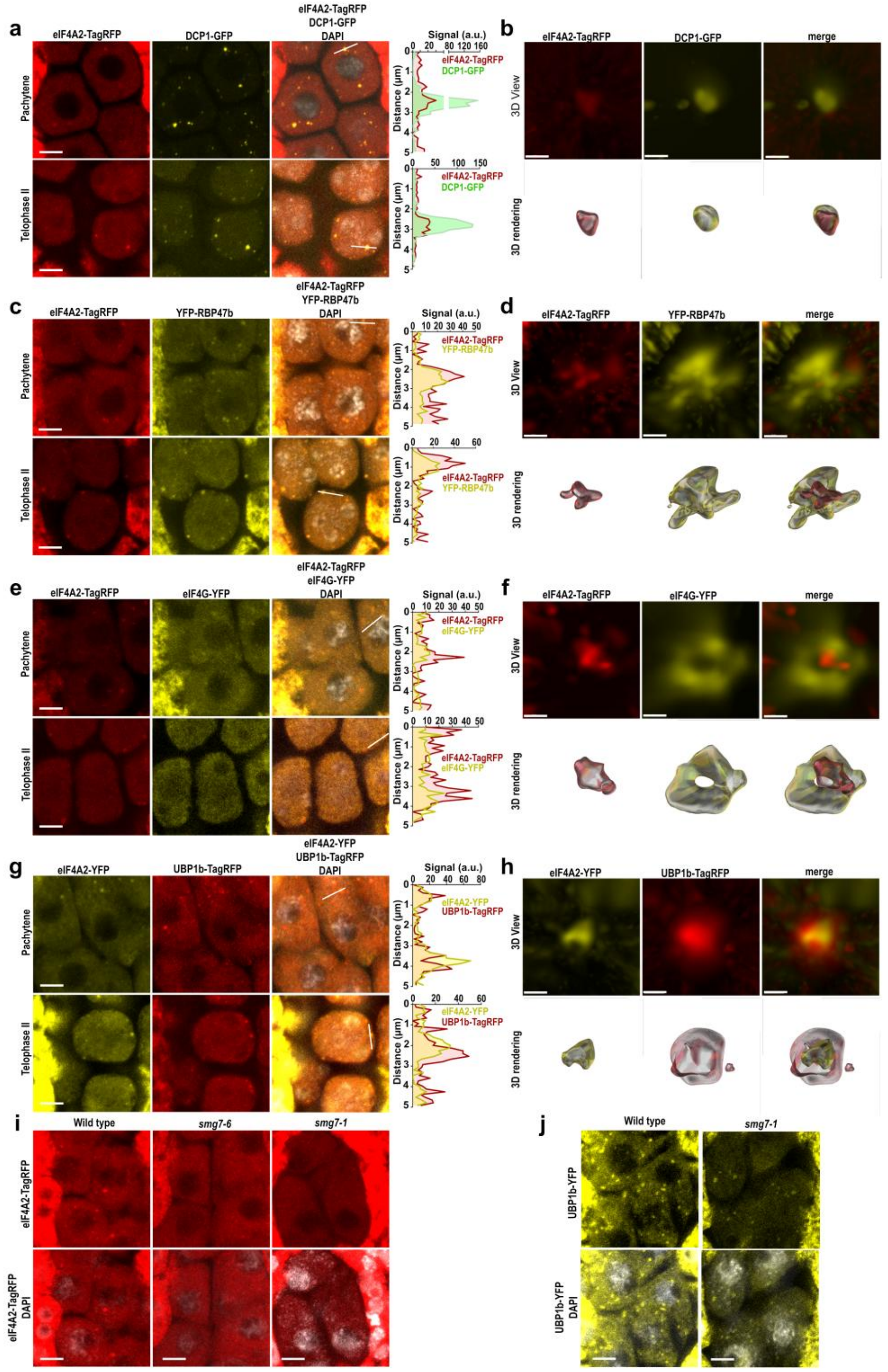

a,**c**,**e**,**g** Confocal micrographs of root cells co-expressing indicated combinations of reporter proteins. Counterstaining with SR2200 dye was used to visualize cell walls. Micrographs depicting pachytene and telophase II meiocytes are shown, and are representative of multiple observations (>20). Diagrams on the right show superimposed intensity profiles of YFP and TagRFP signals measured along the lines indicated in the corresponding micrographs. Each diagram serves as a representative of multiple observations. Scale bar = 10 µm. **b**,**d**,**f**,**h** Super-resolution micrographs of indicated protein condensates corresponding to pachytene (**b**,**f**), zygotene (**d**) or late leptotene (**h**) meiocytes visualized by 3D view and 3D rendering using Imaris software. Scale bar = 0.5 µm. **i**, Confocal micrographs of

Arabidopsis pachytene meiocytes of wild type, *smg7-6* and *smg7-1* plants expressing eIF4A2-TagRFP. Shown micrographs are representative of multiple observations (>20). Scale bars, 5 μm. **j,** Confocal micrographs of *Arabidopsis* pachytene meiocytes of wild type and *smg7-1* plants expressing UBP1b-YFP. Shown micrographs are representative of multiple observations (>20). Scale bars, 5 μm. Source data are provided as a Source Data file.

Supplementary Fig. 10: Downregulation of eIF4A1 enhances TDM1 localization to M-bodies and facilitates meiotic exit.

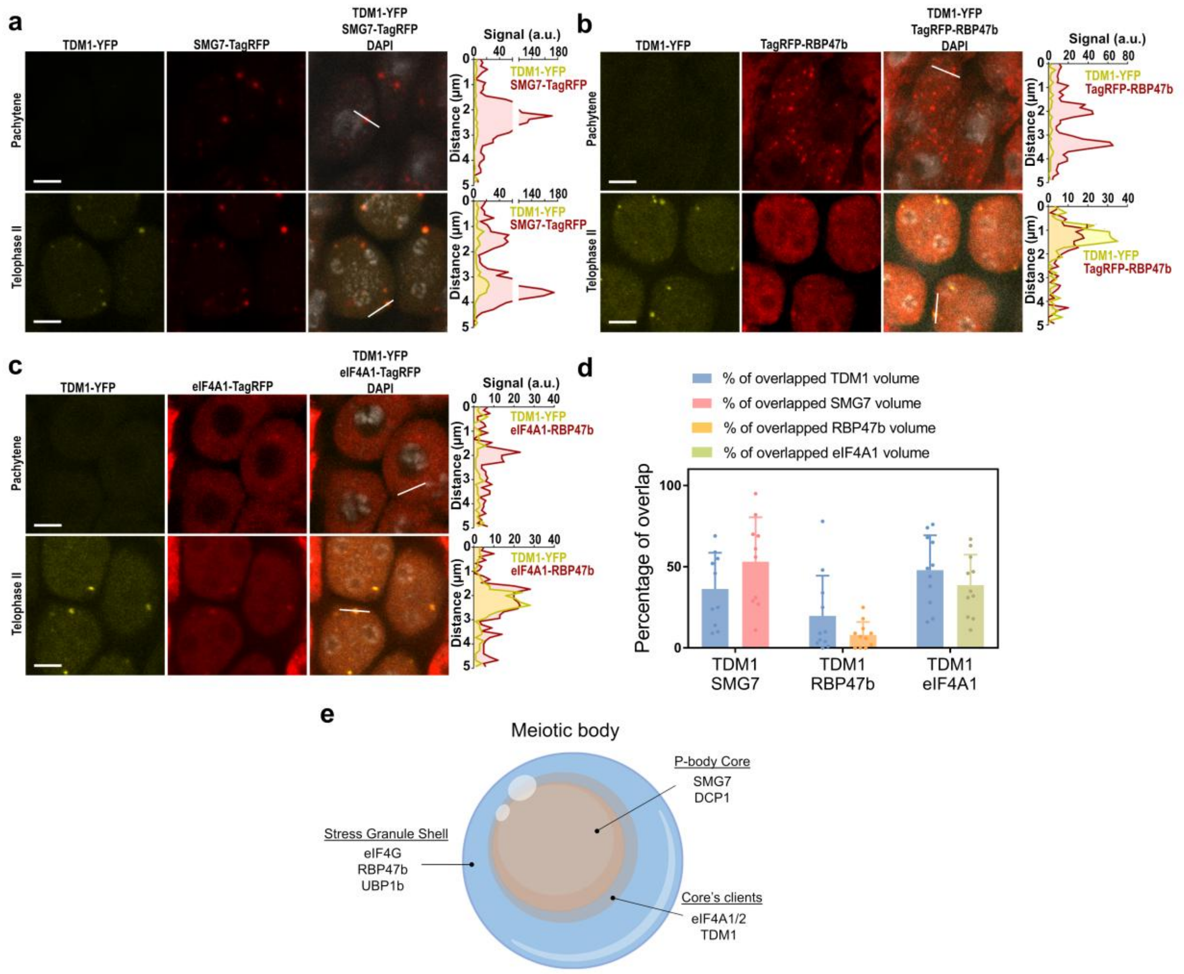

a-**c**, Confocal micrographs of *Arabidopsis* telophase II meiocytes co-expressing TDM1-YFP and SMG7-TagRFP (**a**), TagRFP-RBP47b (**b**) or eIF4A1-TagRFP (**c**). Micrographs depicting pachytene and telophase II meiocytes are shown, and are representative of multiple observations (>20). Diagrams on the right show superimposed intensity profiles of YFP and TagRFP signals measured along the lines indicated in the corresponding micrographs. Each diagram serves as a representative of multiple observations. Scale bar = 5 µm. **d**, Percentages of volume overlaps between indicated protein condensates in meiocytes from super-resolution microscopy (mean, SD, n = 10 associated condensates). **e**, Graphical representation of the M-body substructures, with the identified components. Source data are provided as a Source Data file.

Supplementary Fig. 11: The mutants *eif4a1* and *eif4a2* exhibit minor meiotic defects.

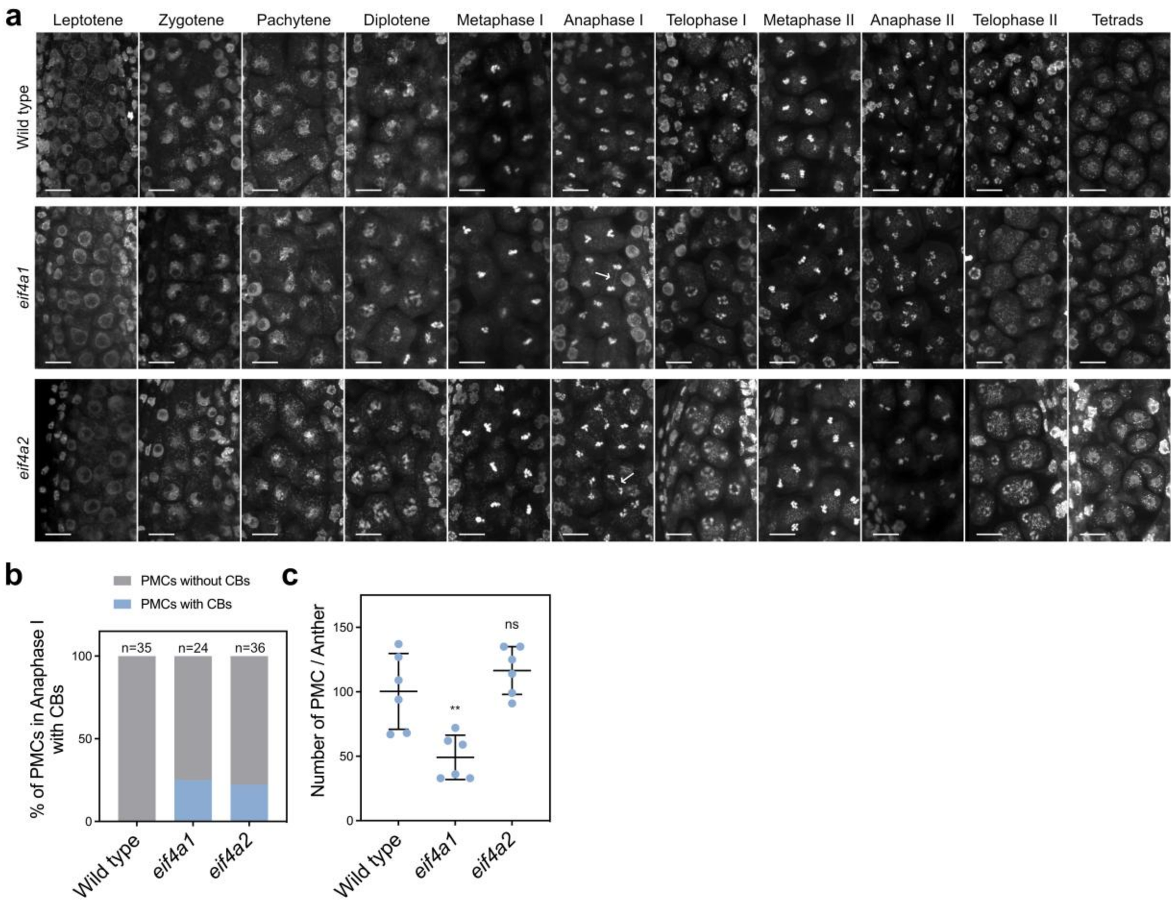

a, Confocal micrographs of *Arabidopsis* anthers of wild type, *eif4a1* and *eif4a2* mutants, showing the indicated meiotic stages. DNA is counterstained with DAPI. Arrows indicate chromatin bridges (CBs) in anaphase I. Each micrograph is representative of a minimum of 3 observed lobes with meiocytes in the depicted stage. **b,** Quantification of chromosome bridges in anaphase I. Scale bars = 10 μm. **c**, number of pollen mother cells per one anther of wild type, *eif4a1* and *eif4a2* mutants (mean, SD, n = 6 anthers; ns $\geq$ 0.05, **p $<$ 0.01, two-tailed unpaired Student's t test). Source data are provided as a Source Data file.

Supplementary Table 1. Mass spectrometry analysis of the SMG7 interactome.

Accession	Locus	Description	Coverage	Number of Peptides (Unique)	Fold Change (Replica 1)	Fold Change (Replica 2)	Fold Change (Replica 3)
A9QM73	At5G19400	SMG7	0,50	46(46)	qual	qual	qual
P41377	At1G54270	eIF4A2 - Eukaryotic translation initiation factor 4A-2	0,63	28(1)	2,93	3,10	2,77
F4JEL5	AT3G13920	eIF4A1 - Eukaryotic translation initiation factor 4A-1	0,77	33(9)	2,93	3,72	2,80
P21218	AT4G27440	PORB - Protochlorophyllide reductase B	0,50	19(15)	2,67	4,80	2,67
Q944S1	AT1G59990	RH22 - DEAD-box ATP-dependent RNA helicase 22	0,32	17(17)	3,94	2,87	4,29
Q948R9	AT3G17170	RFC3 - Regulator of fatty acid composition 3	0,38	10(10)	qual	3,19	2,64
F4I3P9	AT1G63680	MURE	0,35	21(21)	7,19	qual	3,80
Q9LPG6	AT1G53500	RHM2	0,33	20(7)	2,62	7,31	4,16
Q9LH76	AT3G14790	RHM3	0,32	19(7)	2,70	3,57	4,31
P92948	At1G09770	CDC5 - Cell division cycle 5-like protein	0,19	13(13)	2,57	2,75	qual
F4ICK7	AT1G32130	IWS1 - Transcription elongation factor (TFIIS) family protein	0,26	11(11)	qual	qual	qual
F4KFP7	At5G24060	Pentatricopeptide repeat-containing protein-like protein	0,19	8(8)	qual	qual	qual
B3H778	At4G24830	Argininosuccinate synthase	0,09	3(3)	qual	qual	qual
Q9M0A7	AT4G30530	GGP1 - Gamma-glutamyl peptidase 1	0,18	4(4)	qual	qual	qual
Q9C522	At3G06650	ACLB1 - ATP-citrate lyase B-1	0,07	4(4)	qual	qual	qual
B3H757	AT4G29810	MKK2 - Mitogen-activated protein kinase kinase 2	0,09	3(3)	qual	qual	qual
Q39048	At4G24510	ECERIFERUM 2	0,05	2(2)	qual	qual	qual
Q8LEF3	At5G46030	ELF1 - Transcription elongation factor 1 homolog	0,30	1(1)	qual	qual	qual
Q9LXN4	At3G44530	HIRA	0,02	1(1)	4,59	qual	qual

List of proteins identified by LC–MS in pull-downs from wild type and *SMG7:MYC* plants using EZview™ Red Anti-c-Myc Affinity Gel. The list includes the proteins with ≥2.5 enrichment (FC) in all the three independent experiments. qual = qualitative enriched (only present in the sample from *SMG7:MYC* plants).

Supplementary Table 2. List of primers used in this study.

PRIMER	SEQUENCE (5'->3')
UBP1b.BamHI.V2.F	TTATGATATCTATGCAGAGGTTGAAGCAGCAGC
UBP1b.6.R	GGGGGAGATTTCCACTTGGGATTGG
eIF4A1.Prom.F	CACCCTGCGTCAGCCGATCCGAATTCG
eif4A1.nostop.r	CAGCAGATCGGCCACGTTCGAAGGC
eif4A2.Promoter.Topo.F	CACCAAGCCAACACTCACCCTGCGTCC
eiF4A2.nostop.r	CAGCAAATCAGCCACGTTTGAGGG
DCP1_FP	CACCTGGTCTTAACAAGGCAGGCA
DCP1_RP	TTGTTGAAGTGCATTTTGTAAAGTTCGGGTA
UBPb1.EcoRI.F.V1	GGAAACAAAATGCGATGC
UBPb1EcoRV.R.V2	CTGGTAGTACATGAGCTGCTGCGCG
RBP47b_cacc_topo_F_V2	CACCATGCAGACAACCAACGGCTC
RBP47b_stop_R	TCAATTCTCCCCATGATAGTTGTTGG
smg7_del_14-3-3_topoATG	CACCATGTATGAAAAATTGTTTGTGCCCTCC
smg7.TOPO.STOP.R	TCACACAAAGTGACGACTCGACC
EIF4A1.topo.F	CACCATGGCAGGATCTGCACCAGAAGGC
EIF4A2.topo.F	CACCATGGCAGGATCCGCACCGGAAGGAAC
EIF4B1.TOPO.F.real	CACCATGTCGAAAGCTTGGGGTGGAATTGG
EIF4B1.NOSTOP.R	CCATCCTTCCCTAGAGGAAG

The primers that were produced in the present study were procured from the company Merck.
