# Supplementary material for: SMG7 and eIF4A constitute a homeostatic module controlling P-body condensation and function of meiotic bodies
Source: Nat Commun. 2026 Apr 21;17:5477. doi: 10.1038/s41467-026-72218-w (PMC13284301; doi:10.1038/s41467-026-72218-w)
Supplement: Supplementary file 2 — Description of Additional Supplementary File [file 41467_2026_72218_MOESM2_ESM.pdf]

Description of Additional Supplementary File

Supplementary Movie 1. Live imaging of UBP1b-YFP condensation during meiosis Video acquired using light sheet microscopy showing the localization of UBP1bYFP during meiosis. Scale bar = 20 μm
