# Supplementary material for: SMG7 and eIF4A constitute a homeostatic module controlling P-body condensation and function of meiotic bodies
Source: Nat Commun. 2026 Apr 21;17:5477. doi: 10.1038/s41467-026-72218-w (PMC13284301; doi:10.1038/s41467-026-72218-w)
Supplement: Supplementary file 4 — Reporting Summary [file 41467_2026_72218_MOESM4_ESM.pdf]

nature portfolio

Corresponding author(s): Karel Riha

Last updated by author(s): Feb 24, 2026

Reporting Summary

Nature Portfolio wishes to improve the reproducibility of the work that we publish. This form provides structure for consistency and transparency in reporting. For further information on Nature Portfolio policies, see our Editorial Policies and the Editorial Policy Checklist.

Statistics

For all statistical analyses, confirm that the following items are present in the figure legend, table legend, main text, or Methods section.

n/a	Confirmed	
☐	☒	The exact sample size (*n*) for each experimental group/condition, given as a discrete number and unit of measurement
☒	☐	A statement on whether measurements were taken from distinct samples or whether the same sample was measured repeatedly
☐	☒	The statistical test(s) used AND whether they are one- or two-sided<br>*Only common tests should be described solely by name; describe more complex techniques in the Methods section.*
☒	☐	A description of all covariates tested
☒	☐	A description of any assumptions or corrections, such as tests of normality and adjustment for multiple comparisons
☐	☒	A full description of the statistical parameters including central tendency (e.g. means) or other basic estimates (e.g. regression coefficient) AND variation (e.g. standard deviation) or associated estimates of uncertainty (e.g. confidence intervals)
☐	☒	For null hypothesis testing, the test statistic (e.g. *F*, *t*, *r*) with confidence intervals, effect sizes, degrees of freedom and *P* value noted<br>*Give P values as exact values whenever suitable.*
☒	☐	For Bayesian analysis, information on the choice of priors and Markov chain Monte Carlo settings
☒	☐	For hierarchical and complex designs, identification of the appropriate level for tests and full reporting of outcomes
☒	☐	Estimates of effect sizes (e.g. Cohen's *d*, Pearson's *r*), indicating how they were calculated

Our web collection on statistics for biologists contains articles on many of the points above.

Software and code

Policy information about availability of computer code

Data collection	ZEN Black (Zeiss)
Data analysis	Imaris (Oxford Instruments)<br>FIJI<br>MaxQuant software (v1.6.17.0)

For manuscripts utilizing custom algorithms or software that are central to the research but not yet described in published literature, software must be made available to editors and reviewers. We strongly encourage code deposition in a community repository (e.g. GitHub). See the Nature Portfolio guidelines for submitting code & software for further information.

Data

Policy information about availability of data

All manuscripts must include a data availability statement. This statement should provide the following information, where applicable:

- Accession codes, unique identifiers, or web links for publicly available datasets
- A description of any restrictions on data availability
- For clinical datasets or third party data, please ensure that the statement adheres to our policy

Mass spectrometry proteomics data were deposited to the ProteomeXchange Consortium via PRIDE 85 partner repository under dataset identifier PXD061406 and

10.6019/PXD061406.

Research involving human participants, their data, or biological material

Policy information about studies with human participants or human data. See also policy information about sex, gender (identity/presentation), and sexual orientation and race, ethnicity and racism.

Reporting on sex and gender	*Use the terms sex (biological attribute) and gender (shaped by social and cultural circumstances) carefully in order to avoid confusing both terms. Indicate if findings apply to only one sex or gender; describe whether sex and gender were considered in study design; whether sex and/or gender was determined based on self-reporting or assigned and methods used. Provide in the source data disaggregated sex and gender data, where this information has been collected, and if consent has been obtained for sharing of individual-level data; provide overall numbers in this Reporting Summary. Please state if this information has not been collected. Report sex- and gender-based analyses where performed, justify reasons for lack of sex- and gender-based analysis.*
Reporting on race, ethnicity, or other socially relevant groupings	*Please specify the socially constructed or socially relevant categorization variable(s) used in your manuscript and explain why they were used. Please note that such variables should not be used as proxies for other socially constructed/relevant variables (for example, race or ethnicity should not be used as a proxy for socioeconomic status). Provide clear definitions of the relevant terms used, how they were provided (by the participants/respondents, the researchers, or third parties), and the method(s) used to classify people into the different categories (e.g. self-report, census or administrative data, social media data, etc.) Please provide details about how you controlled for confounding variables in your analyses.*
Population characteristics	*Describe the covariate-relevant population characteristics of the human research participants (e.g. age, genotypic information, past and current diagnosis and treatment categories). If you filled out the behavioural & social sciences study design questions and have nothing to add here, write "See above."*
Recruitment	*Describe how participants were recruited. Outline any potential self-selection bias or other biases that may be present and how these are likely to impact results.*
Ethics oversight	*Identify the organization(s) that approved the study protocol.*

Note that full information on the approval of the study protocol must also be provided in the manuscript.

Field-specific reporting

Please select the one below that is the best fit for your research. If you are not sure, read the appropriate sections before making your selection.

☒ Life sciences ☐ Behavioural & social sciences ☐ Ecological, evolutionary & environmental sciences

For a reference copy of the document with all sections, see nature.com/documents/nr-reporting-summary-flat.pdf

Life sciences study design

All studies must disclose on these points even when the disclosure is negative.

Sample size	The minimum sample size was six, in most cases ten, in some cases more. The sample size for an experimental category was limited by accessibility of analyzed cells and time necessary to acquire parallel measurement for comparison between categories. Nevertheless, based on our experience, the sample size chosen in our study is sufficient to detect larger differences, which form the base for the major conclusions in the paper.
Data exclusions	We did not apply data exclusion.
Replication	We usually present comparisons that were measured in one experiment which includes multiple biological replicates. Nevertheless, the experiments were repeated multiple times validating reproducibility.
Randomization	In most cases samples were randomly chosen, assuming acquired images were of a good technical quality.
Blinding	The FRET experiment was performed with blinded design, where experimentator did not know the identify of the measured samples. For all the other experiments, experimentator knew the identify of the sample.

Reporting for specific materials, systems and methods

We require information from authors about some types of materials, experimental systems and methods used in many studies. Here, indicate whether each material, system or method listed is relevant to your study. If you are not sure if a list item applies to your research, read the appropriate section before selecting a response.

Materials & experimental systems

n/a	Involved in the study	
☐	☒	Antibodies
☒	☐	Eukaryotic cell lines
☒	☐	Palaeontology and archaeology
☒	☐	Animals and other organisms
☒	☐	Clinical data
☒	☐	Dual use research of concern
☐	☒	Plants

Methods

n/a	Involved in the study	
☒	☐	ChIP-seq
☒	☐	Flow cytometry
☒	☐	MRI-based neuroimaging

Antibodies

Antibodies used

anti-MYC polyclonal antibody (ab9106 Abcam) (Dilution 1:1000)
EZview™ Red Anti-c-Myc Affinity Gel (50 µl of affinity gel / 10 mg total protein)
anti GFP monoclonal antibody (3H9) (AB_10773374 ChromoTek) (Dilution 1:1000)

Validation

These are well established commercial antibodies

Plants

Seed stocks

Arabidopsis thaliana ecotype Col-0 and mutants derived from this ecotype were used in the study. Mutants strains represent T-DNA insertions and we refer to the papers where these mutants were for the first time described.

Novel plant genotypes

In this study, we generated the following Arabidopsis thaliana reporter lines: eIF4A1:YFP, eIF4A2:YFP, eIF4A1:TagRFP, eIF4A2:TagRFP, DCP1:G3GFP, UBP1b:YFP, UBP1b:TagRFP, RPS5A::YFP:RBP47b, and RPS5A::TagRFP:RBP47b.The reporter lines were generated by transforming either wild-type or the corresponding mutant plants using the floral dip method. Dozens of T1 transgenic seedlings were selected and prescreened for expression. T2 seeds were analyzed for transgene segregation, and at least three lines with a homozygous single-locus insertion were examined for marker protein expression in the T3 generation. A single representative line was used for further genetic crosses and for the analyses shown in this study.

Authentication

Describe any authentication procedures for each seed stock used or novel genotype generated. Describe any experiments used to assess the effect of a mutation and, where applicable, how potential secondary effects (e.g. second site T-DNA insertions, mosiacism, off-target gene editing) were examined.
