# Supplementary material for: SMG7 and eIF4A constitute a homeostatic module controlling P-body condensation and function of meiotic bodies
Source: Nat Commun. 2026 Apr 21;17:5477. doi: 10.1038/s41467-026-72218-w (PMC13284301; doi:10.1038/s41467-026-72218-w)

nature portfolio

Peer Review File

SMG7 and eIF4A constitute a homeostatic module controlling P-body condensation and function of Meiotic bodies

Corresponding Author: Professor Karel Říha

This file contains all reviewer reports in order by version, followed by all author rebuttals in order by version.

Version 0:

Reviewer comments:

Reviewer #1

(Remarks to the Author)
This study elucidates a novel regulatory module involving SMG7 and eIF4A helicases in controlling P-body dynamics and meiotic bodies (M-bodies) formation in Arabidopsis. The authors demonstrate that SMG7 recruits eIF4A to P-bodies, where eIF4A limits condensate size and suppresses stress granule (SG) formation. Crucially, they identify M-bodies as composite structures with a P-body core and SG-like shell, linking their remodeling to meiotic exit via TDM1 recruitment. The work is conceptually innovative, technically rigorous (employing super-resolution imaging and FLIM-FRET), and physiologically significant. And it unveils a sophisticated mechanism for RNP granule homeostasis with profound implications for RNA metabolism and meiosis. The discovery of M-bodies as hybrid condensates is particularly insightful. While the findings are compelling, I do have a number of specific suggestions/questions about the manuscript for clarity.

Major:
1.The study demonstrates that heat shock promotes significant enlargement of SMG7-marked P-bodies and enhanced partitioning of eIF4A1/2 into cytoplasmic speckles in root cells. However, the reversibility of this condensation process remains unaddressed. Do P-bodies revert to their basal size/composition upon recovery from heat stress?
2.The current study proposes a model in which SMG7-eIF4A forms a negative feedback loop to regulate P-body/M-body size (Figure 8). However, this mechanistic model primarily relies on mutant phenotype observations and qualitative colocalization analyses, lacking quantitative experimental support. FRAP or FLIP experiments could be performed to quantitatively assess how eIF4A influences the dynamic equilibrium of P-bodies/M-bodies.
3.It has been demonstrated that irreversible sequestration of eIF4F by TDM1 in P-bodies to enforce meiotic exit (Albert Cairo, 2022), while the study proposes eIF4A as an RNA chaperone that limits condensate size and may resolve translational repression. I am wondering whether eIF4A activity facilitate the release of eIF4F from TDM1-containing P-bodies during meiotic exit or stress recovery?
4.Lines 138-139: The authors showed that the interaction with SMG7 was significantly reduced in the non-phosphorylatable eIF4A1S146A mutant and completely abolished when both Thr145 and Ser146 were mutated. SMG7 recruits eIF4A via its 14-3-3 domain (Fig. 1), however the functional consequences of eIF4A phosphorylation (Ser146) could be further explored. Does phospho-mimetic eIF4A (S146D) alter P-body size or TDM1 recruitment?
5.The study demonstrates that M-bodies, which composite condensates with a P-body core and SG-like shell, could be able to orchestrate meiotic exit by recruiting TDM1 to inhibit translation. It should be more carefully discussed for the influences of DNA damage or meiotic DSBs on M-body assembly, composition, and function, particularly under heat stress conditions.

Minor:
1.Several references are missing volume or page details (Refs 2 and 6), and the format of preprints (such as bioRxiv, e.g. Ref 64) needs to be standardized.
2.Fig. 2c: The eIF4A1–TagRFP signal after heat shock is too sparse to be compelling. In the same panel, the RFP fluorescence is faint while the chromatin stain is overpowering, making the localization information unclear.
3.The conclusions in Fig. 3c may be under-powered due to the limited sample size. Please increase the number of biological replicates to strengthen statistical reliability, and maintain consistent n-values across all experiments for comparative validity.
4.Fig. 4a: The chromosome morphology in zygotene-to-diplotene meiocytes is currently rather diffuse. Could the authors replace these panels with sharper, better-contrasted DAPI images in which each stage is clearly distinguishable?
5.Fig. 6a: why less prominent eIF4A1–TagRFP spots were selected for signal analysis instead of the brighter spots?

6.Lines 208-209: “eIF4A1/2 formed distinct foci that were most prominent during pachytene”. While in Figure 4a, the eIF4A1 foci appear visually indistinguishable between pachytene and diplotene stages. Even the foci of diplotene can be seen with the naked eye to be more obvious than that of pachytene. Please provide representative images that clearly demonstrate this distinction."

Reviewer #2

(Remarks to the Author)
Cairo et al. explore the connection between SMG7 (a P-body factor) and eIF4A, traditionally considered a SG (stress granule) factor, in somatic and meiotic plant cells. They show convincing evidence that SMG7 and eIF4A physically interact via the 14-3-3 domain of SMG and residues on 4A that can be phosphorylated. This is similar to how SMG7 interacts with phosphorylated UPF1, which suggests that 4A and UPF1 binding might be mutually exclusive and SMG7 can recruit different factors to P-bodies. Surprisingly, eIF4A, which has been shown to localize only to SGs in several different eukaryotic model systems, accumulates in both SGs and P-bodies in Arabidopsis root cells. The authors use super resolution microscopy to get a closer look at what appears to be co-localization of factors via confocal microscopy. They see 4A co-localize with P-bodies factors, but extend beyond the P-bodies. This extra 4A co-localizes with SG markers, suggesting that eIF4A localizes to adjacent P-bodies and stress granules, rather than SGs alone. They show strong genetic evidence suggesting that WT 4A antagonizes formation of these P-bodies. They then look at the formation of these granules in meiotic cells and find that eIF4A, SMG7, and DCP1 co-localize together and then stress granule factors (at least RBP47 and 4A) interact around the edge of the P-body. Their previous work shows that SMG7 recruits TDM1 to P-bodies and that this recruitment is important for the normal progression of meiosis. Intriguingly, during meiosis, 4A mutants show increased recruitment of TDM1 to P-bodies, which correlates with decreases viability, presumably from too much translation repression. SMG7 mutants also show decreased viability, presumably from not recruiting enough TDM1 and not repressing translation enough. As expected by this model, the 4A mutant can suppress the fertility defect of the SMG7 mutant.

This is an interesting story in several ways: (1) uncovers a role for 4A in P-body formation and meiosis, (2) uses super resolution microscopy to get a clearer view of sub-compartmentalization of P-bodies and SGs, something that isn’t common yet in granule papers, so more examples of granule topography are informative, and (3) expands the mechanistic understanding of how SMG7 impacts meiosis. I think this paper will interest scientists from a range of RNA fields, as well as those interested in meiosis and stress responses.

The experiments are solid and reinforce the conclusions for the most part. I disagree (or need clarification in the writing of the paper), on a few statements, which are detailed below. I think the paper could benefit from more quantification of some of the data in the paper.

Comment 1: The super resolution microscopy is interesting and gives new insight into the sub-domains within these P-bodies/SGs. However, we are only shown one granule group per condition. It would be nice to have some sort of quantitation to help the reader determine whether the example shown is representative of all granules in the sample, or a portion of granules. For example, in Fig. 2A, in the control vs. heat shock conditions, how many SMG7 granules show eIF4A co-localization? How many of those co-localized signals show a larger area of eIF4A around the SMG7 core (as shown in Fig 2B), indicating association with an adjacent SG? How does that compare to SMG7 co-localization with another SG factor (Fig. 2H)? Such quantitation will show how representative the sample granule is and start to characterize these foci for future studies.

Comment 2: In general, it is not clear to me how robust the sample sizes are in each experiment, so communicating this in figure legends or the methods needs to be improved. Examples:

a. The histograms next to the confocal sections represent the signal transecting one or two granules. But are these representative of evaluations of more granules per sample? More than one biological replicate? This needs to be clarified to the reader can gage experimental rigor. I assume the authors did look at multiple biological replicates and multiple granules across those different replicates (and pictures are representative examples) and simply need to clarify that in the figure legend.
b. Fig. 1h, does the sample size refer to cells, FRET events, etc? The authors should go through ALL the figure legends and specify what exactly “n” equals… individual granules, cells, tissue areas, etc.
Comment 3: In Fig 3., eIF4A mutants cause an increase in RBP47 and SMG7 granule intensity. The authors should show that the 4A mutant doesn’t increase the levels of the RBP47 and SMG7 proteins, as over-expression of granule proteins can drive granule formation.
c. Minor point: the eif4a1/2 mutants are null mutants? Please clarify that in the strain description.

Comment 4: The authors use observations like the eif4a mutants in Fig. 3 to support the idea that eIF4A is an RNA chaperone. This model would be strengthened by testing rescue of the bright granule intensity in the eif4a mutants with WT eIF4A (where intensity should decrease in the mutant) versus a eIF4A point mutant that impairs helicase activity (which should still show an increase in granule brightness, like the original eif4a mutant. Since protein-protein, protein-RNA, and RNA-RNA interactions can drive granule formation, I don’t think they’ve definitely shown that eIF4A works as an RNA chaperone, rather than as a protein interaction partner in granules. Either more specific 4A mutants should be tested, or alternative hypotheses should be stated in the paper.

Comment 5: The authors state: “Consistent with observations in root cells, P-bodies in meiocytes lacking either helicase were significantly larger and more abundant compared to those in the wild type (Fig. 4d-f).” But it isn’t clear that they are

directly measuring size or abundance for this data set.

a. Changes to “Average condensate signal intensity/meiocyte” (Fig. 4e) could be caused by brighter, but similarly sized granules (so, more 4A in the same sized granule) or an increase in granule size. If the authors want to make the case that some conditions have “significantly larger” granules, then granule area would be a better measure.
b. Fig. 4e/f and Fig. 5c-e are based on quantitation from only 6 cells? That is low, unless meiocytes are very difficult to track. The results would be more compelling if representing a larger number of cells and if the authors shared how many biological replicates they’ve examined. In mammalian and yeast studies, papers normally include analysis of >100 cells. Is this unreasonable for meiocytes?
c. Likewise, increases “total condensation signal /meiocyte” could reflect more abundant, larger, and/or brighter foci. To determine whether granules are “more abundant” the authors should quantitate the number of granules per cell. I think this is an important piece of data, as it also allows us to understand how many granules are included in the analysis of 6 meiocytes in this data set.
d. (which sounds like the average brightness of granules in a cell, but only 6 cells are analyzed per sample as indicated in the figure legend? The brightness of the granule could be influenced by size or by increasing the number of 4A proteins in the granule.
e. As for determining whether granules are “more abundant” then they should be quantitating the number of granules per cell? I think this is an important piece of data, as it also allows us to understand how many granules are included in the analysis of 6 meiocytes.
f. In fig 4b, there seem to be more SMG7 foci than 4A foci. Since the authors are characterizing these “M-bodies” for the first time, it would be helpful to quantitate the number of each foci per cell and the percent of foci that have both 4A and SMG7 localization.
Comment 6: The authors coin a new term “Meiotic bodies or M-bodies.” Is an M-body the foci with SMG7 (P-body like) or the foci with RBP47 (SG like) or the combination of the two, where there is a P-body like core and a stress granule shell? That should be clarified
g. I’m not sure a new granule name is warranted here, as only a few protein’s localizations have been tested and the authors didn’t verify that these granules contain RNA. But I acknowledge that is a matter of opinion and time will tell if the term will be adopted by others.
h. There is some based characterization of M-bodies that is lacking that I think are important to understand how common they are. In Pachytene and Diplotene, what percentage of cells have M-bodies? What is the average number of SMG7 (or some other marker of the core) foci/cell and the average number of SG shells per cell? What percentage of SMG7 foci have SG shells associated with them? Quantitating these metrics for at least Zygotene, pachytene, diplotene, and Metaphase I would be a nice description of the emergence of these foci.
i. In Fig 6F, how do you know these are M bodies and not P-bodies? They are present in the absence of stress and way more numerous that eIF4A foci. Do they have SG-like “shells?” Without being able to identify M-bodies specifically, I don’t think this control is clearly showing that M-bodies can form in this smg7 mutant. It’s possible that the smg7 mutant is blocking M-body formation, and in the absence of that nucleating ability, more traditional P-bodies form. Either way is interesting.

Comment 7: Throughout the paper, the overlap between two proteins as shown in the histograms seems to parallel what the authors see with super resolution microscopy… that is, when the peaks differ in height, there seems to be sub-compartmentalization within the foci, even if they look like they overlap in the lower resolution confocal images. Have the authors done enough of this to determine if the histograms could be a predictor of compartmentalization? If so, it might be interesting to look at 4A1/RBP47 co-localization by super resolution microscopy in both pachytene and telophase II (one is shown in Fig. 6d), as the histograms show very different overlap in these two stages. I leave it to the authors to decide if that is interesting or not (or a strong enough correlation to trust changes in histogram patterns). Either way, please specify whether the granule in Fig. 6d comes from a cell in pachytene or telophase II

Comment 8: There is a lot of great data here, but the authors need to spend time relabeling it. The scale of the text is way too small. If someone is viewing this in print, some things are very hard if not impossible (cyan line in Fig 1g as just one example) to discern. Even looking at this electronically, it was a hassle to zoom in and out as excessively as was needed to view the data well and then, for example, read the figure legend.

Comment 9: Minor points:
a. Page 8 line 266 has a typo: eFF4A2 should be eIF4A2
b. Many figures list eIF4A1/2 in the title, but only 4A1 is in the figure. It would be helpful to add a line to the figure legend that comparable data on 4A2 is available in Extended Fig. X
c. Fig. 7: the labels in 7G, which describe what prominent cell state the authors observe in that mutant, were helpful. Can these be added to 7j? While the 4A mutant didn’t suppress the viability defect of tdm1-4, the morphology of the cells seem to change. Is that change interesting and tell you anything about the effect of 4A and TDM1? I leave it to the authors to decide if there is anything here they feel worth commenting on in the paper.

Despite what looks like a long list of changes/suggestions, it does not indicate a poor opinion of the paper. I do think the findings are interesting and novel. But clearer description of sample sizes and replicates will increase the rigor of the results. And, where practical, quantitation will considerably strengthen the conclusions within the paper.

Reviewer #3

(Remarks to the Author)
The manuscript by Cairo et al. reports the association of P-bodies and stress granules—referred to by the authors as

“meiotic-bodies”—in Arabidopsis PMCs. The authors show that the RNA helicases eIF4A1 and eIF4A2 localize to meiotic P-bodies, directly interact with SMG7 via its 14-3-3 pocket. They further demonstrate that eif4a1 or eif4a2 mutants display increased P-body size and number, suggesting a conserved role of eIF4A in modulating SGs/P-bodies similar to that in human cells. Incorporation of eIF4A into meiotic P-bodies is dependent on SMG7. Previous work showed that TDM1 is recruited to meiotic P-bodies by SMG7 and is required for translation inhibition and meiotic exit. Here, the authors find that TDM1 localization to meiotic P-bodies is reduced in the smg7-6 hypomorphic mutant but increased in the eif4a1 mutant, and that eif4a1 suppresses the meiotic phenotype of smg7-6. Based on these results, they propose a model in which SMG7 and eIF4A form a negative feedback loop to regulate P-bodies.

Overall, the manuscript is well written, the figures are clear, and the study addresses a significant role of eIF4A in modulating meiotic P-bodies and, consequently, meiotic exit. However, the proposed SMG7–eIF4A feedback loop is not fully supported by the presented data, and the text is somewhat repetitive in places, warranting condensation.

Major Comments
1. The conclusion that eif4a mutation suppresses the smg7-6 phenotype via a SMG7–eIF4A negative feedback loop is not the only possible explanation. Given that SMG7 binds both TDM1 and eIF4A via its 14-3-3 pocket, the data could also be explained by competition between TDM1 and eIF4A for SMG7 binding. Additional experiments are needed to distinguish between these possibilities.
2. The authors describe an association between P-bodies and SGs in meiotic PMCs, and introduce the term “meiotic-bodies” for these condensates. I do not find it appropriate to introduce a new term in this case. Physical association between P-bodies and SGs is well-documented in both plants and animals, typically facilitating mRNA exchange between granules. Moreover, no unique functional property of these meiotic granules is shown that would distinguish them from other associations. Given the already crowded terminology in the field, I recommend avoiding new names unless a clear functional distinction is established.
3. The manuscript presents extensive 3D constructions illustrating physical overlap between condensates. While the visualizations are convincing, quantitative analysis—such as calculating percentage overlap volumes—would strengthen the conclusions. Since 3D constructions are already available, such measurements should be straightforward.
4. I wonder whether eif4a1 or eif4a2 mutants have any meiotic phenotypes. Additionally, given the authors’ observation that plant eIF4A proteins are less abundant than other eIF4F subunits, it would be informative to examine the effect of eIF4A1/2 overexpression. If the negative feedback loop model is correct, one would expect overexpression to phenocopy smg7 mutants.

Minor Comments
1. The text is generally clear but contains some redundancy, especially in repeated descriptions of root and PMC imaging. Words such as “unexpected” and “surprising” are overused and could be minimized.
2. Lines 232–233: the phrase “in these lines” is confusing. Figure 5 shows six panels of YFP–RBP4b lines—do these all correspond to the overexpression line? If RBP4b is not expressed during meiosis, the rationale for using an overexpression line should be stated, and the figure panels or legends should clearly indicate this. As currently written, readers may interpret YFP–RBP4b as a translational fusion expressed from the native promoter, as in other lines in the manuscript.
3. Figures 4d–f: the meiotic stage (telophase II or pachytene) should be stated in the legend. Are P-bodies reduced at both stages in the eif4a mutants?

Version 1:

Reviewer comments:

Reviewer #2

(Remarks to the Author)
The authors have made a great effort to increase the clarity and rigor of the paper by clarifying sample size and even increasing it in some cases. They have added a substantial amount of quantitation which reinforces their interpretations. This type of quantitation is time intensive, but worth the effort and I think their work has substantially strengthened the paper. As stated in more depth in the original review, I find the results interesting and novel, especially now that they are strengthen by the added experiments and quantitation.

There are a few places where the authors completed preliminary experiments to address reviewers concerns and decided not to include the data because of caveats of the experimental system. I appreciate the experimental attempts and am satisfied with their explanations of why they are not pursuing these experiments further for this paper or including this data in the paper. While I agree with reviewer 1 that further exploration of phosphorylation of 4A and its effect on SMG7 is interesting, I support the authors’ decision to pursue this area in future work. They already have a substantial body of work here.

I did not identify any new concerns.

Two small questions/requests on Supp. Fig. 3 and its relationship to Fig. 2:
First, Supp Fig. 3d should have inferential statistics to produce a p-value. The authors observe that heat shock causes

accumulation of eIF4A in a SG-like shell round the SMG7 P-bodies core, while in the control condition, eIF4A largely coincides with the SMG7/P-body signal. If this observation in the sample images is consistent across granules and tissue samples, then the percentage of 4A overlap with SMG7 (in Supp Fig. 3d) should decrease in heat shock conditions compared to the control. That decrease looks slight for 4A1 (and even slighter in 4A2 comparisons) in Supp. Fig 3d. Is this difference (in % of overlapped 4A volume in control vs. HS) statistically significant? If yes, then the next paragraph isn't relevant.

If the difference is not statistically significant, then the results for Figure 2a need to be modified to be more conservative (e.g., that there appears to a be a trend in decreased overlap, but it is not statistically significant). And the authors should address why the sample images in Fig. 2b are much more compelling than the quantitation in Supp. Fig. 3d. Is that because of variability among foci or some variability or sensitivity issue in the quantitation that is masking the differences observed in the 3D view and rendering seen in Fig. 2b? For example, is the signal threshold for 4A and SMG7 presence lower/more sensitive when quantitating overlap compared to the signal levels required to appear in the 3D rendering? That could inflate the apparent overlap between 4A and SMG7 in the stress condition.

Second, I'm surprised that the percentage of overlapped 4A volume (with SMG7) is less than 50% in the control condition (Supp. Fig 3d), as they show strong co-localization in the image and 3D rendering in Fig. 2b (and Supp Fig. 3a shows nearly all 4A foci have some SMG7 signal). Is there some variability in the foci or noise in the data? Or is there some sub-compartmentalization of 4A and SMG7 within P-bodies in the control condition? Can the authors give some context to this in the text?

Reviewer #3

(Remarks to the Author)
In my view, both my comments and those of Reviewer 1 have been satisfactorily addressed. The authors have provided sufficient new data and revision to resolve the main concerns raised during the previous round of review.

Open Access This Peer Review File is licensed under a Creative Commons Attribution 4.0 International License, which permits use, sharing, adaptation, distribution and reproduction in any medium or format, as long as you give appropriate credit to the original author(s) and the source, provide a link to the Creative Commons license, and indicate if changes were made.
In cases where reviewers are anonymous, credit should be given to 'Anonymous Referee' and the source.
The images or other third party material in this Peer Review File are included in the article's Creative Commons license, unless indicated otherwise in a credit line to the material. If material is not included in the article's Creative Commons license and your intended use is not permitted by statutory regulation or exceeds the permitted use, you will need to obtain permission directly from the copyright holder.
To view a copy of this license, visit https://creativecommons.org/licenses/by/4.0/

Response to reviewer´s comments

We would like to thank all reviewers for their supportive evaluations and constructive suggestions. To address the reviewers' comments, we added new experimental data and revised the manuscript. We believe that the additional work prompted by the reviewers has significantly improved the quality of the manuscript. The changes made to the manuscript are highlighted in yellow in the marked copy.

Reviewer #1 (Remarks to the Author):

1.The study demonstrates that heat shock promotes significant enlargement of SMG7-marked P-bodies and enhanced partitioning of eIF4A1/2 into cytoplasmic speckles in root cells. However, the reversibility of this condensation process remains unaddressed. Do P-bodies revert to their basal size/composition upon recovery from heat stress?

Indeed, the recovery of P-body condensation to pre-stress conditions has not yet been examined in plants. To address this, we added new experimental data showing the dynamics of P-body condensation in root cells during recovery from heat shock, using the SMG7–TagRFP marker (new Supplementary Fig. 3b–c).

2.The current study proposes a model in which SMG7-eIF4A forms a negative feedback loop to regulate P-body/M-body size (Figure 8). However, this mechanistic model primarily relies on mutant phenotype observations and qualitative colocalization analyses, lacking quantitative experimental support. FRAP or FLIP experiments could be performed to quantitatively assess how eIF4A influences the dynamic equilibrium of P-bodies/M-bodies.

Thank you for this suggestion. We examined how eIF4A1 affects the mobility of SMG7–TagRFP using FRAP and did not observe major changes in fluorescence recovery within P-bodies (new Supplementary Fig. 4). This result is consistent with a model in which eIF4A primarily modulates RNA condensation, whereas SMG7 mobility within P-bodies may be largely determined by protein–protein interactions. Ideally, we would look at the mobility of RNAs between the cytoplasm and condensates, but such experiments are currently beyond technical feasibility in our experimental system.

3.It has been demonstrated that irreversible sequestration of eIF4F by TDM1 in P-bodies to enforce meiotic exit (Albert Cairo, 2022), while the study proposes eIF4A as an RNA chaperone that limits condensate size and may resolve translational repression. I am wondering whether eIF4A activity facilitate the release of eIF4F from TDM1-containing P-bodies during meiotic exit or stress recovery?

In fact, in our previous study, we showed that TDM1-mediated sequestration of eIFiso4G2 in P-bodies is only transient and that the signal quickly recovers. In contrast, TDM1 appears to form a more stable component of P-bodies. Based on these observations, we proposed that TDM1 and eIFiso4G2 represent a scaffold–client relationship and suggested a model in which TDM1 transiently recruits eIF4F RNPs into P-bodies and remodels them to inhibit translation.

We performed FRAP experiments in protoplasts to test whether eIF4A1 affects the retention of these factors in P-bodies. Our results indicate that eIF4A1 has no effect on the dynamics of either eIFiso4G2 or TDM1, at least in protoplasts. This is consistent with the observation that eIFiso4G2 recruitment is primarily based on its direct interaction with TDM1, which may not be influenced by eIF4A1.

However, we have decided not to include these data in the manuscript. Performing FRAP experiments on eIFiso4G2 in meiocytes is technically unfeasible, and the protoplast system mimics only certain aspects of M-bodies and has important limitations (such as overexpression of the proteins involved and the absence of RNAs normally regulated by M-bodies). Therefore, we do not wish to overinterpret these data and prefer not to include them in the manuscript.

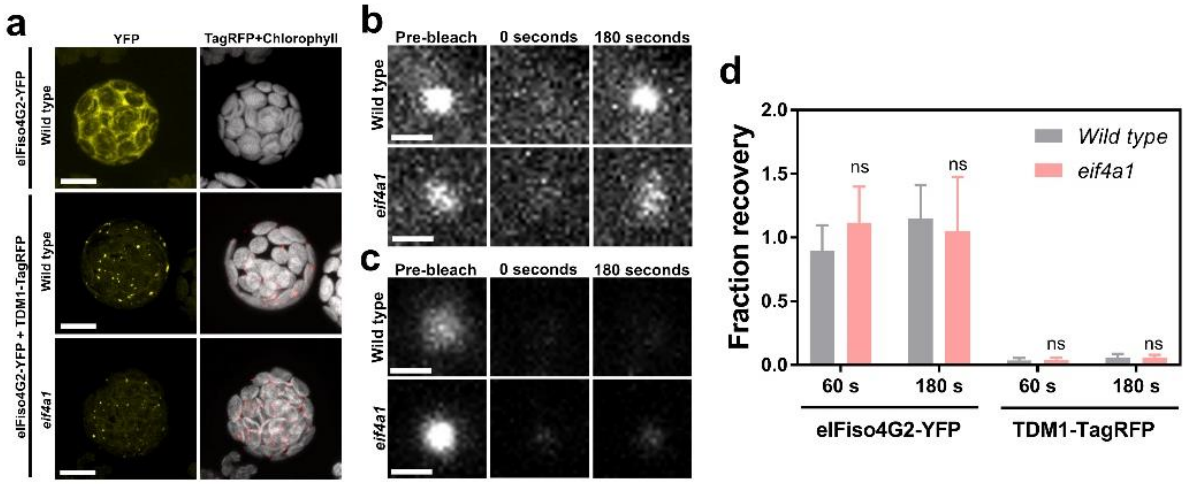

a, *Arabidopsis* mesophyll protoplasts from wild-type and *eif4a1* plants transiently transfected with pGWB-eIFiso4G2-YFP and pGWB-TDM1-TagRFP. Scale bar, 10 μm. **b–d,** FRAP analysis of eIFiso4G2-YFP (**b**) and TDM1-TagRFP (**c**) signals in P-bodies of wild-type and *eif4a1* protoplasts. **b, c,** Confocal micrographs of eIFiso4G2-YFP (**b**) and TDM1-TagRFP (**c**) condensates before photobleaching and at 0 s or 180 s after photobleaching. Scale bar, 1 μm. **d,** Fraction of fluorescence recovered at 60 s and 180 s for eIFiso4G2-YFP and TDM1-TagRFP (mean ± SD, n = 8 bleached condensates; ns ≤ 0.05, Student's *t*-test).

4.Lines 138-139: The authors showed that the interaction with SMG7 was significantly reduced in the non-phosphorylatable eIF4A1S146A mutant and completely abolished when both Thr145 and Ser146 were mutated. SMG7 recruits eIF4A via its 14-3-3 domain (Fig. 1), however the functional consequences of eIF4A phosphorylation (Ser146) could be further explored. Does phospho-mimetic eIF4A (S146D) alter P-body size or TDM1 recruitment?

We agree with the reviewer on the importance to explore further the regulation of eIF4A1/2 by phosphorylation. It is potentially very exciting research direction not only with regards to regulation of meiosis, but also during stress responses. Nevertheless, we plan to address this regulation in a follow up work, as full exploration of this direction will require substantial experimental effort and resources.

5.The study demonstrates that M-bodies, which composite condensates with a P-body core and SG-like shell, could be able to orchestrate meiotic exit by recruiting TDM1 to inhibit translation. It

should be more carefully discussed for the influences of DNA damage or meiotic DSBs on M-body assembly, composition, and function, particularly under heat stress conditions.

Thank you for this suggestion. We agree that M-bodies likely act as important coordinators of meiotic processes, including recombination, integrating both developmental and environmental cues to ensure the formation of viable spores under varying conditions. While the present study does not experimentally address these aspects, it provides an important foundation for follow-up investigations.

We have recently published a preprint describing an RNA-binding protein, CDM1, which associates with M-bodies and represses the translation of several meiotic transcripts, presumably through their sequestration in M-bodies (https://doi.org/10.1101/2025.08.17.670746). We show that *cdm1* mutants exhibit severe defects in chromosome behavior, including condensation, pairing and cytokinesis, suggesting a key role for M-body–mediated post-transcriptional regulation in these processes.

Furthermore, one recent paper and our preprint in collaboration with Dr. Geelen lab (published after the submission of this manuscript and referring to this study for discovery and description of M-bodies) showed that the meiotic cyclin TAM is sequestered into M-bodies in response to elevated temperature, and that this sequestration is crucial for proper segregation of meiotic chromosomes (https://doi.org/10.1101/2022.08.11.503651 and doi:10.1126/sciadv.adr5694). Together, these studies further highlight the importance of M-bodies in multiple aspects of meiosis. We now briefly refer to these studies in the Discussion, but we refrain from a more extensive elaboration on these topics, as they were not experimentally addressed here and the manuscript is already quite comprehensive.

Minor:

1.Several references are missing volume or page details (Refs 2 and 6), and the format of preprints (such as bioRxiv, e.g. Ref 64) needs to be standardized.

We updated the details and formatted all bioRxiv preprint citations in Nature style using EndNote.

2.Fig. 2c: The eIF4A1–TagRFP signal after heat shock is too sparse to be compelling. In the same panel, the RFP fluorescence is faint while the chromatin stain is overpowering, making the localization information unclear.

We have exchanged the representative picture in Fig 2c.

3.The conclusions in Fig. 3c may be under-powered due to the limited sample size. Please increase the number of biological replicates to strengthen statistical reliability, and maintain consistent n-values across all experiments for comparative validity.

We have increased the sample size from n =6 to n = 10.

4.Fig. 4a: The chromosome morphology in zygotene-to-diplotene meiocytes is currently rather diffuse. Could the authors replace these panels with sharper, better-contrasted DAPI images in which each stage is clearly distinguishable?

We have updated the confocal micrographs of zygotene, pachytene, and diplotene to improve the identification of the meiotic stages. We would like to note that, for the analysis of M-bodies, we use formaldehyde fixation to preserve the 3D structure of meiocytes. This technique is not well suited for detailed chromosome morphology. Our DAPI imaging is therefore intended solely to distinguish the individual meiotic stages, and we are very confident in these assignments.

5.Fig. 6a: why less prominent eIF4A1–TagRFP spots were selected for signal analysis instead of the brighter spots?

We have used line profiles from bodies of different sizes to illustrate the diversity of signals. In this particular case, the line happened to cross two bodies. We have now updated the profile to feature more prominent bodies (now Fig. 7a).

6.Lines 208-209: "eIF4A1/2 formed distinct foci that were most prominent during pachytene". While in Figure 4a, the eIF4A1 foci appear visually indistinguishable between pachytene and diplotene stages. Even the foci of diplotene can be seen with the naked eye to be more obvious than that of pachytene. Please provide representative images that clearly demonstrate this distinction."

We corrected the sentence to clearer reflect presented data. In addition, we provide a new figure with the quantification of eIF4A1 foci during meiosis (new Supplementary Fig. 6a).

Reviewer #2 (Remarks to the Author):

Comment 1: The super resolution microscopy is interesting and gives new insight into the sub-domains within these P-bodies/SGs. However, we are only shown one granule group per condition. It would be nice to have some sort of quantitation to help the reader determine whether the example shown is representative of all granules in the sample, or a portion of granules. For example, in Fig. 2A, in the control vs. heat shock conditions, how many SMG7 granules show eIF4A co-localization? How many of those co-localized signals show a larger area of eIF4A around the SMG7 core (as shown in Fig 2B), indicating association with an adjacent SG? How does that compare to SMG7 co-localization with another SG factor (Fig. 2H)? Such quantitation will show how representative the sample granule is and start to characterize these foci for future studies.

We now support the super-resolution and co-localization analyses with quantitative data. Specifically, we have added a new Supplementary Fig. 3 that includes quantification of SMG7 and eIF4A1 association under normal and heat-stress conditions in root cells (Supplementary Fig. 3a). In addition, we quantified the overlap of associated bodies formed by the indicated proteins in root cells using super-resolution microscopy (Supplementary Figs. 3d–f).

To quantitatively describe the behavior of M-bodies, we added new figures that include the association of SMG7 and RBP47b or eIF4A1 in pachytene and telophase II as observed by confocal microscopy (new Supplementary Fig. 6d-i), as well as quantification of the volume overlap of different M-body protein constituents from super-resolution microscopy (Fig. 6a). We

also added new panel “e” to Supplementary Fig. 8e to quantify the overlap of eIF4A1/2 helicases with P-body and stress-granule markers within M-bodies.

Comment 2: In general, it is not clear to me how robust the sample sizes are in each experiment, so communicating this in figure legends or the methods needs to be improved. Examples:

a. The histograms next to the confocal sections represent the signal transecting one or two granules. But are these representative of evaluations of more granules per sample? More than one biological replicate? This needs to be clarified to the reader can gage experimental rigor. I assume the authors did look at multiple biological replicates and multiple granules across those different replicates (and pictures are representative examples) and simply need to clarify that in the figure legend.

We now provide proper quantification data on signal associations from confocal microscopy micrographs, as well as on the signal overlaps between the condensates from super-resolution microscopy. The profiles help to visualize the signal association in representative images.

b. Fig. 1h, does the sample size refer to cells, FRET events, etc? The authors should go through ALL the figure legends and specify what exactly “n” equals… individual granules, cells, tissue areas, etc.

We have specified in all the figures what “n” represents.

Comment 3: In Fig 3., eIF4A mutants cause an increase in RBP47 and SMG7 granule intensity. The authors should show that the 4A mutant doesn’t increase the levels of the RBP47 and SMG7 proteins, as over-expression of granule proteins can drive granule formation.

Thank you for this comment. We now include quantification of the partition fraction, which measures the proportion of total protein localized in granules (new Figs. 3g, i, q). This analysis, which normalizes for total protein levels, shows that mutation of the helicase substantially increases the fraction of the protein present in granules.

c. Minor point: the eif4a1/2 mutants are null mutants? Please clarify that in the strain description.

The *eif4a1/2* mutants carry T-DNA insertions within the respective genes, which results in the expression of severely truncated non-functional proteins. We now state this in the Results section when these mutants are introduced, and we also refer to the paper that extensively characterized these alleles.

Comment 4: The authors use observations like the eif4a mutants in Fig. 3 to support the idea that eIF4A is an RNA chaperone. This model would be strengthened by testing rescue of the bright granule intensity in the eif4a mutants with WT eIF4A (where intensity should decrease in the mutant) versus a eIF4A point mutant that impairs helicase activity (which should still show an increase in granule brightness, like the original eif4a mutant. Since protein-protein, protein-RNA,

and RNA-RNA interactions can drive granule formation, I don't think they've definitely shown that eIF4A works as an RNA chaperone, rather than as a protein interaction partner in granules. Either more specific 4A mutants should be tested, or alternative hypotheses should be stated in the paper.

We attempted to perform a complementation experiment using a transient expression system in mesophyll protoplasts. A specific helicase-deficient mutant of eIF4A has not been described in any system. However, based on studies in human cells (Tauber et al., Cell 2020), we generated analogous mutations in *Arabidopsis* eIF4A1 to produce ATPase-deficient (eIF4A1^{E183Q}) and RNA-binding–deficient (eIF4A1^{R368Q}) variants and tested their ability to limit P-body condensation in protoplasts.

As shown in the figure below, co-transfection of wild-type eIF4A1-YFP together with SMG7-RFP, used as a P-body marker, tended to reduce the intensity of the SMG7-RFP signal in P-bodies. In contrast, this effect was not apparent for the constructs carrying the above-mentioned mutations. These observations are consistent with the idea that P-body size limitation requires the ability of eIF4A to bind and dissociate from RNA, supporting its proposed role as an RNA chaperone.

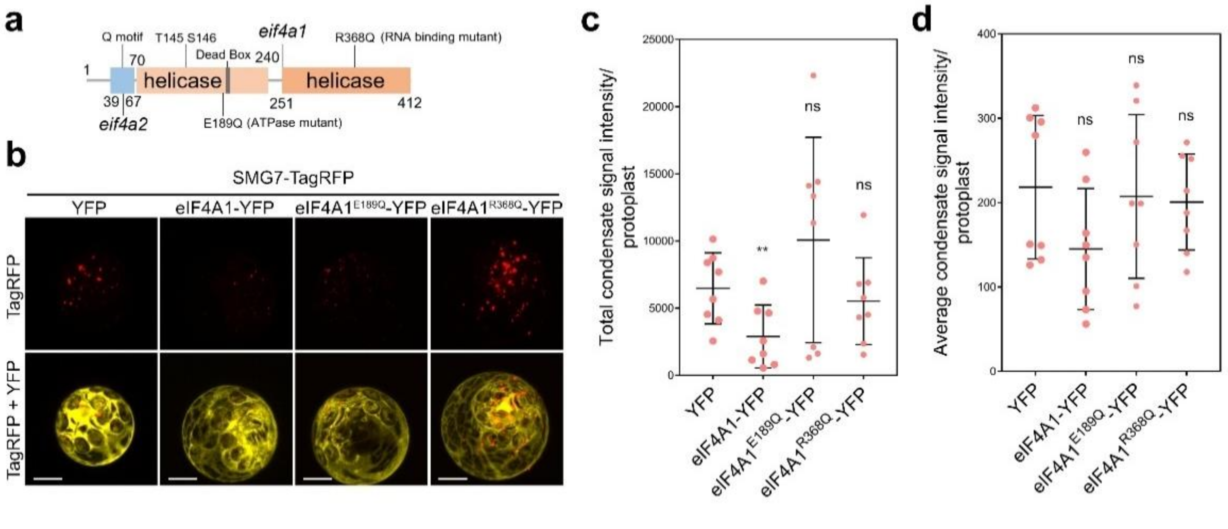

a, Domain structure of eIF4A1 indicating the T-DNA insertion site of the *eif4a1* mutant (and *eif4a2*, which has identical domains and mutation positions), as well as the mutations in the ATPase-deficient mutant (E189Q) and the RNA-binding mutant (R368Q). **b**, *Arabidopsis* mesophyll protoplasts from wild-type and *eif4a1* plants transiently transfected with vectors expressing the indicated proteins. Scale bar, 10 µm. **c, d**, Dot plots showing total condensate signal intensity per protoplast (**c**) and average condensate signal intensity per protoplast (**d**). Data are presented as mean ± SD (n = 8 protoplasts; ns, $p > 0.05$; $^{**}p \leq 0.01$, Student's *t*-test).

However, the protoplast system proved to be poorly suited for rigorous quantitative analysis. We observed very high variability in SMG7-RFP signal intensity between protoplasts (likely reflecting transfection efficiency), which precluded reliable quantification and the detection of statistically significant differences. Furthermore, to observe any trends, we excluded protoplasts with very high SMG7-RFP expression from the analysis and included only those with lower signal intensity. For these reasons, the results are difficult to interpret conclusively, and we have decided not to include these data in the manuscript.

Accordingly, in the Discussion we include an alternative explanation for eIF4A function beyond its proposed role as an RNA chaperone. Nevertheless, in light of published data from human cells (Tauber et al., 2020), the RNA chaperone model remains the most parsimonious explanation.

Comment 5: The authors state: "Consistent with observations in root cells, P-bodies in meiocytes lacking either helicase were significantly larger and more abundant compared to those in the wild type (Fig. 4d-f)." But it isn't clear that they are directly measuring size or abundance for this data set.

a. Changes to "Average condensate signal intensity/meiocyte" (Fig. 4e) could be caused by brighter, but similarly sized granules (so, more 4A in the same sized granule) or an increase in granule size. If the authors want to make the case that some conditions have "significantly larger" granules, then granule area would be a better measure.

This is a valid point. To address this issue and provide more comprehensive quantification, we added charts showing the average condensate volume and the average number of condensates per meiocyte (Fig. 4g, h). This analysis showed that SMG7 foci are more numerous in *eif4a* mutants. Many of these foci are small in size and intensity, which collectively reduces the relative contribution of larger condensates in the dataset, where size differences are more apparent. Therefore, we included two additional charts showing the volume and average signal intensity of the largest foci in the top quartile (Q4; Fig. 4i, j). In these condensates, the differences in size and signal intensity are statistically more significant than in the full condensate population.

We have also included statistics on P-body and stress-granule number and volume in roots (new Fig. 3d, f, i, k, n, p) and on the volume of RBP47b foci in Fig. 5 (new Fig. 5d).

b. Fig. 4e/f and Fig. 5c-e are based on quantitation from only 6 cells? That is low, unless meiocytes are very difficult to track. The results would be more compelling if representing a larger number of cells and if the authors shared how many biological replicates they've examined. In mammalian and yeast studies, papers normally include analysis of >100 cells. Is this unreasonable for meiocytes?

Plant meiocytes are much less accessible than cultivated mammalian cells, and in addition, we compare meiocytes at the same meiotic stage. Only one or two floral buds in an entire inflorescence contain meiotic cells, and only a few cells within each bud are at the appropriate stage. Nevertheless, we have increased the number of cells analyzed: in Fig. 4e–j, the sample size has been increased to 14 meiocytes, and in Fig. 5c–f to 10 meiocytes.

c. Likewise, increases "total condensation signal /meiocyte" could reflect more abundant, larger, and/or brighter foci. To determine whether granules are "more abundant" the authors should quantitate the number of granules per cell. I think this is an important piece of data, as it also allows us to understand how many granules are included in the analysis of 6 meiocytes in this data set.

As indicated above, in addition to Fig. 4, we have added new charts showing the number of granules per cell or per cell volume in Fig. 3 and Fig. 5.

d. (which sounds like the average brightness of granules in a cell, but only 6 cells are analyzed per sample as indicated in the figure legend? The brightness of the granule could be influenced by size or by increasing the number of 4A proteins in the granule.

e. As for determining whether granules are "more abundant" then they should be quantitating the number of granules per cell? I think this is an important piece of data, as it also allows us to understand how many granules are included in the analysis of 6 meiocytes.

We believe these correspond to the same questions addressed in points a–c.

f. In fig 4b, there seem to be more SMG7 foci than 4A foci. Since the authors are characterizing these "M-bodies" for the first time, it would be helpful to quantitate the number of each foci per cell and the percent of foci that have both 4A and SMG7 localization.

Thank you for this question. We now provide a new Supplementary Fig. 6 showing the number of SMG7 foci per meiocyte across all meiotic stages (Supplementary Fig. 6b). We performed a similar analysis for eIF4A1 and RBP47b (Supplementary Fig. 6a,c). In addition, we quantified the percentage of colocalization between SMG7 and eIF4A1 based on confocal micrographs at pachytene and telophase II (Supplementary Fig. 6d–f), and we quantified the ratio of overlap between eIF4A1/2 volumes and the volumes of P-body core and SG-shell components (Supplementary Fig. 8e) from the super-resolution microscopy.

Comment 6: The authors coin a new term "Meiotic bodies or M-bodies." Is an M-body the foci with SMG7 (P-body like) or the foci with RBP47 (SG like) or the combination of the two, where there is a P-body like core and a stress granule shell? That should be clarified

g. I'm not sure a new granule name is warranted here, as only a few protein's localizations have been tested and the authors didn't verify that these granules contain RNA. But I acknowledge that is a matter of opinion and time will tell if the term will be adopted by others.

This is an excellent point. We assume that the definition of M-bodies will likely evolve as additional data become available. In the present manuscript, we introduce the term M-bodies for the first time and define them operationally as condensates consisting of a P-body core surrounded by a SG shell.

Our most recent data, as well as results from collaborators, indicate that the organization of these structures is more intricate and dynamically regulated during meiosis. For example, in a recent preprint deposited on bioRxiv (https://doi.org/10.1101/2025.08.17.670746), we describe an additional component of M-bodies, the RNA-binding protein CDM1, which forms a distinct phase at the surface of the P-body core. These findings suggest that M-bodies can form triphasic assemblies consisting of a P-body core surrounded by two separate phases formed by CDM1 and SG components, and further demonstrate the importance of CDM1 for meiotic progression.

In another preprint prepared in collaboration with Dr. Geelen (University of Ghent), we show that under heat stress the meiotic cyclin TAM associates with M-bodies in the interfacial region

between the P-body core and the SG shell (https://doi.org/10.1101/2022.08.11.503651). Both of these studies refer to the present manuscript as the work in which M-bodies were first defined. In addition, a recent study from the Schnittger laboratory showed that the association of TAM with these structures (referred to in that work as stress granules, based on the marker used for colocalization) is important for proper meiotic progression under heat stress.

Taken together, these studies, including the new data presented here (see also Fig. 6 and our response to Reviewer 3), support the conclusion that these assemblies are not simply transient associations of stress granules and P-bodies, but rather represent complex and highly dynamic RNP condensates whose composition and function change over the course of meiosis. For this reason, we do not anticipate that the term M-body will denote a single invariant condensate architecture. Instead, it will likely refer more broadly to a class of meiotic condensates characterized by a P-body core associated with one or more surrounding phases, such as SG and/or CDM1-like layers. Coining the term M-bodies therefore has a pragmatic advantage, as it avoids confusion with canonical stress granules or P-bodies while providing a flexible framework that can accommodate emerging structural and functional diversity.

Ultimately, as the reviewer correctly notes, time will determine how broadly this terminology is adopted by the field.

h. There is some based characterization of M-bodies that is lacking that I think are important to understand how common they are. In Pachytene and Diplotene, what percentage of cells have M-bodies? What is the average number of SMG7 (or some other marker of the core) foci/cell and the average number of SG shells per cell? What percentage of SMG7 foci have SG shells associated with them? Quantitating these metrics for at least Zygotene, pachytene, diplotene, and Metaphase I would be a nice description of the emergence of these foci.

We have improved the characterization of M-bodies by providing new experimental data. As mentioned in (f), we quantified the number of foci at all meiotic stages based on the SMG7 and RBP47b signals (Supplementary Fig. 6a-c). We also quantified the percentage of colocalization of SMG7 and RBP47b from confocal micrographs at pachytene and telophase II (Supplementary Fig. 6g–i). In addition, using super-resolution micrographs, we quantified the degree of overlap between the core and shell components (Fig. 6a). Further characterization is provided in our response to Reviewer 3, question 2.

i. In Fig 6F, how do you know these are M bodies and not P-bodies? They are present in the absence of stress and way more numerous that eIF4A foci. Do they have SG-like “shells?” Without being able to identify M-bodies specifically, I don’t think this control is clearly showing that M-bodies can form in this smg7 mutant. It’s possible that the smg7 mutant is blocking M-body formation, and in the absence of that nucleating ability, more traditional P-bodies form. Either way is interesting.

Indeed, more DCP1 foci are detected than eIF4A1 bodies, which is also reflected in the higher number of detected SMG7 foci (see new Supplementary Fig. 6a-b). This difference may be partly explained by the higher partitioning of SMG7 and DCP1 into condensates, resulting in bright, easily detectable foci. In contrast, eIF4A1 shows lower partitioning and retains a substantial cytoplasmic signal, producing less prominent foci. Consequently, smaller SMG7/DCP1 foci can be detected more readily, whereas small eIF4A1 foci may be less apparent (see new

Supplementary Fig 6e-f). Thus, while we have shown that DCP1 resides in the core of M-bodies surrounded by an SG shell (Fig. 5j; new Fig. 6; Fig. 7b; and Supplementary Fig. 9b,d,f), we cannot determine whether small DCP1 foci represent M-bodies (with a shell) or canonical P-bodies.

To address whether M-bodies can be nucleated in the absence of SMG7, we analyzed the UBP1b stress-granule marker in the *smg7-1* background. We observed the formation of UBP1b foci under normal conditions (new Supplementary Fig. 9j), indicating that M-bodies can form even in the absence of SMG7.

Comment 7: Throughout the paper, the overlap between two proteins as shown in the histograms seems to parallel what the authors see with super resolution microscopy… that is, when the peaks differ in height, there seems to be sub-compartmentalization within the foci, even if they look like they overlap in the lower resolution confocal images. Have the authors done enough of this to determine if the histograms could be a predictor of compartmentalization? If so, it might be interesting to look at 4A1/RBP47 co-localization by super resolution microscopy in both pachytene and telophase II (one is shown in Fig. 6d), as the histograms show very different overlap in these two stages. I leave it to the authors to decide if that is interesting or not (or a strong enough correlation to trust changes in histogram patterns). Either way, please specify whether the granule in Fig. 6d comes from a cell in pachytene or telophase II

Indeed, shifted peaks in histogram analyses can sometimes hint at sub-compartmentalization, particularly when the effect is pronounced. However, in many cases this approach does not provide sufficient spatial resolution, which is why we employed super-resolution microscopy. Comparing different meiotic stages with respect to granule sub-compartmentalization is an excellent suggestion. In our dataset, we did not observe substantial differences in the localization of the M-body core and shell markers analyzed in this study. Nevertheless, we did detect interesting shifts in other markers, such as the previously mentioned CDM1 protein. A more systematic and precise characterization of these changes in terms of condensate sub-compartmentalization will be an important future research direction.

We now specify the meiotic stages for all micrographs of M-bodies presented in the manuscript.

Comment 8: There is a lot of great data here, but the authors need to spend time relabeling it. The scale of the text is way too small. If someone is viewing this in print, some things are very hard if not impossible (cyan line in Fig 1g as just one example) to discern. Even looking at this electronically, it was a hassle to zoom in and out as excessively as was needed to view the data well and then, for example, read the figure legend.

Thank you for this remark. We are now doing our best; however, the amount of data presented and the number of image panels are already high, and fitting them into figures of reasonable size is challenging.

Comment 9: Minor points:

a. Page 8 line 266 has a typo: eFF4A2 should be eIF4A2

Corrected.

b. Many figures list eIF4A1/2 in the title, but only 4A1 is in the figure. It would be helpful to add a line to the figure legend that comparable data on 4A2 is available in Extended Fig. X

We adjusted the titles of individual Figures to better reflect their content.

c. Fig. 7: the labels in 7G, which describe what prominent cell state the authors observe in that mutant, were helpful. Can these be added to 7j?

We now provide the information (current Fig. 8g).

While the 4A mutant didn't suppress the viability defect of tdm1-4, the morphology of the cells seem to change. Is that change interesting and tell you anything about the effect of 4A and TDM1? I leave it to the authors to decide if there is anything here they feel worth commenting on in the paper.

The reviewer likely refers to the round-shaped cell observed in the double *tdm1 eif4a1* mutant (current Fig. 8j). This difference arises because the two images represent slightly different stages of meiosis II: the tdm1-4 mutant is already undergoing aberrant cytokinesis, whereas the double mutant is in aberrant anaphase/telophase II. Nevertheless, both meiocytes display phenotypes typical of the *tdm1-4* mutant at the respective stages. We now provide labels of these stages the figure.

Despite what looks like a long list of changes/suggestions, it does not indicate a poor opinion of the paper. I do think the findings are interesting and novel. But clearer description of sample sizes and replicates will increase the rigor of the results. And, where practical, quantitation will considerably strengthen the conclusions within the paper.

Reviewer #3 (Remarks to the Author):

Major Comments

1*. The conclusion that eif4a mutation suppresses the smg7-6 phenotype via a SMG7–eIF4A negative feedback loop is not the only possible explanation. Given that SMG7 binds both TDM1 and eIF4A via its 14-3-3 pocket, the data could also be explained by competition between TDM1 and eIF4A for SMG7 binding. Additional experiments are needed to distinguish between these possibilities.*

We agree with the reviewer. At present, we cannot determine whether the enhanced recruitment of TDM1 into M-bodies is due to their increased size or to reduced competition for SMG7 binding

in the absence of one of the eIF4A homologues. We discuss both possibilities in the Discussion section.

2. The authors describe an association between P-bodies and SGs in meiotic PMCs, and introduce the term "meiotic-bodies" for these condensates. I do not find it appropriate to introduce a new term in this case. Physical association between P-bodies and SGs is well-documented in both plants and animals, typically facilitating mRNA exchange between granules. Moreover, no unique functional property of these meiotic granules is shown that would distinguish them from other associations. Given the already crowded terminology in the field, I recommend avoiding new names unless a clear functional distinction is established.

The reviewer raises a valid point that the association of stress granules (SGs) and P-bodies under stress conditions is well documented, and therefore questions the need to introduce a new term. Nevertheless, we believe there are several reasons to do so. The data presented here, as well as those from our follow-up studies mentioned above, indicate that M-bodies represent complex and highly dynamic structures with important functional properties (please see our replies to Reviewer 1, comment 5, and Reviewer 2, comment 6g). These assemblies do not appear to correspond to canonical P-bodies.

Thus, there is a pragmatic reason to use the term M-bodies, as it helps avoid confusion with canonical stress granules or P-bodies. To further demonstrate that M-bodies are distinct from stress-induced assemblies of P-bodies and SGs, we provide new data in the new Fig. 6 analyzing the three-dimensional organization of these structures. While M-bodies clearly exhibit a P-body core surrounded by an SG-like shell, assemblies of SGs and P-bodies that form in response to heat shock in root cells appear instead as juxtaposed condensates without a clear core–shell organization.

3. The manuscript presents extensive 3D constructions illustrating physical overlap between condensates. While the visualizations are convincing, quantitative analysis—such as calculating percentage overlap volumes—would strengthen the conclusions. Since 3D constructions are already available, such measurements should be straightforward.

Thank you for this suggestion. We have now quantified the overlap of protein domains analyzed by super-resolution microscopy and 3D rendering, both in P-bodies and SGs in roots (new Supplementary Fig. 3d-f), as well as in M-bodies (Fig. 6a and Supplementary Figs. 8e and 10d).

4. I wonder whether eif4a1 or eif4a2 mutants have any meiotic phenotypes. Additionally, given the authors' observation that plant eIF4A proteins are less abundant than other eIF4F subunits, it would be informative to examine the effect of eIF4A1/2 overexpression. If the negative feedback loop model is correct, one would expect overexpression to phenocopy smg7 mutants.

We examined meiosis in the *eif4a1* and *eif4a2* mutants in more detail and observed two phenotypes. First, we detected an increased presence of chromatin bridges during anaphase I in both mutants compared to the wild type. Second, we noted a reduced number of meiocytes per anther loculus in *eif4a1* plants. These data are presented in the new Supplementary Fig. 11. We have not generated lines that strongly overexpress the helicases.

Minor Comments

1. The text is generally clear but contains some redundancy, especially in repeated descriptions of root and PMC imaging. Words such as “unexpected” and “surprising” are overused and could be minimized.

We reduced usage of these terms.

2. Lines 232–233: the phrase “in these lines” is confusing. Figure 5 shows six panels of YFP–RBP4b lines—do these all correspond to the overexpression line? If RBP4b is not expressed during meiosis, the rationale for using an overexpression line should be stated, and the figure panels or legends should clearly indicate this. As currently written, readers may interpret YFP–RBP4b as a translational fusion expressed from the native promoter, as in other lines in the manuscript.

The YFP-RBP47b line was the first SG reporter generated and used in our laboratory. At the time, RBP47b was one of the best-established SG markers in *Arabidopsis*, and the commonly used reporter line expressed YFP-RBP47b from a strong 35S promoter. However, because the 35S promoter does not work well in meiosis, we generated a reporter line expressing YFP-RBP47b form a ribosomal protein promoter known to provide consistent expression in meiotic cells. We performed our initial experiments (which usually involved time consuming genetic crosses) using this line. In the meantime, we established additional reporter lines expressing SG markets from their native promoters (UBP1b, eIF4G), which allowed us to validate data obtained with the RBP47B marker. We now explicitly state the use of this promoter in the Results section at the point where the marker was first introduced. Importantly, to date we have not observed any non-standard behavior of the YFP-RBP47b reporter compared with the other SG markers.

3. Figures 4d–f: the meiotic stage (telophase II or pachytene) should be stated in the legend. Are P-bodies reduced at both stages in the eif4a mutants?

Thanks for pointing this out. We focused our quantification on Telophase II because it is a well-defined and relatively short stage, which increases the reliability of the measurements. We have included this information in the figure legend.

Response to reviewer´s comments

Reviewer #1 (Remarks to the Author):

The authors have made a great effort to increase the clarity and rigor of the paper by clarifying sample size and even increasing it in some cases. They have added a substantial amount of quantitation which reinforces their interpretations. This type of quantitation is time intensive, but worth the effort and I think their work has substantially strengthened the paper. As stated in more depth in the original review, I find the results interesting and novel, especially now that they are strengthen by the added experiments and quantitation.

There are a few places where the authors completed preliminary experiments to address reviewers concerns and decided not to include the data because of caveats of the experimental system. I appreciate the experimental attempts and am satisfied with their explanations of why they are not pursuing these experiments further for this paper or including this data in the paper. While I agree with reviewer 1 that further exploration of phosphorylation of 4A and its effect on SMG7 is interesting, I support the authors' decision to pursue this area in future work. They already have a substantial body of work here.

I did not identify any new concerns.

We thank the reviewer for very detailed and thoughtful review—their comments and suggestions have indeed helped us substantially improve the manuscript.

Two small questions/requests on Supp. Fig. 3 and its relationship to Fig. 2:

First, Supp Fig. 3d should have inferential statistics to produce a p-value. The authors observe that heat shock causes accumulation of eIF4A in a SG-like shell round the SMG7 P-bodies core, while in the control condition, eIF4A largely coincides with the SMG7/P-body signal. If this observation in the sample images is consistent across granules and tissue samples, then the percentage of 4A overlap with SMG7 (in Supp Fig. 3d) should decrease in heat shock conditions compared to the control. That decrease looks slight for 4A1 (and even slighter in 4A2 comparisons) in Supp. Fig 3d. Is this difference (in % of overlapped 4A volume in control vs. HS) statistically significant? If yes, then the next paragraph isn't relevant.

If the difference is not statistically significant, then the results for Figure 2a need to be modified to be more conservative (e.g., that there appears to a be a trend in decreased overlap, but it is not statistically significant). And the authors should address why the sample images in Fig. 2b are much more compelling than the quantitation in Supp. Fig. 3d. Is that because of variability among foci or some variability or sensitivity issue in the quantitation that is masking the differences observed in the 3D view and rendering seen in Fig. 2b? For example, is the signal threshold for 4A and SMG7 presence lower/more sensitive when quantitating overlap compared to the signal levels required to appear in the 3D rendering? That could inflate the apparent overlap between 4A and SMG7 in the stress condition.

Second, I'm surprised that the percentage of overlapped 4A volume (with SMG7) is less than 50% in the control condition (Supp. Fig 3d), as they show strong co-localization in the image and 3D rendering in Fig. 2b (and Supp Fig. 3a shows nearly all 4A foci have some SMG7 signal). Is there some variability in the foci or noise in the data? Or is there some sub-compartmentalization of 4A and SMG7 within P-bodies in the control condition? Can the authors give some context to this in the text?

The difference in the percentage of eIF4A–SMG7 overlap between the control and heat stress–treated samples (Supp. Fig. 3D) is statistically significant for eIF4A1 (P = 0.025). Although eIF4A2 shows a similar trend, the difference does not reach statistical significance, primarily due to two outlier samples. We now indicate the P values in Supp. Fig. 3D and include the values for each individual sample as a scatter plot. We believe that these data support the interpretation presented in the main text, which states that "…this expansion became more apparent…".

In addition, two aspects should be considered.

(1) Following heat shock, the overlap between eIF4A and SMG7 would indeed be expected to decrease due to the expansion of eIF4A into stress granules; however, the size of P-bodies also increases, which partially mitigates this effect.

(2) It is important to take into account that quantification of SIM-generated images involves an inherent error caused by the SIM reconstruction process. This is particularly apparent for markers that show lower partitioning between granules and the cytoplasm, such as eIF4A.

We illustrate this in the figure included below. While SIM produces sharp and clearly defined objects in the case of SMG7-RFP, owing to the high partitioning of this protein into P-bodies, the eIF4A signal also shows relatively high background signal in the cytoplasm (it has a lower partitioning into P-bodies). As a result, the corresponding objects are sometimes more difficult to segment and separate into individual entities. In other words, in some samples this may complicate the clear delineation of object boundaries. Since the same image-processing pipeline was applied to all images, the delineation of granule boundaries may not be equally precise in every sample. This source of error may be greater for eIF4A than for SMG7, which may also help explain the approximately 50% overlap in their volumetric co-localization observed in the control sample.

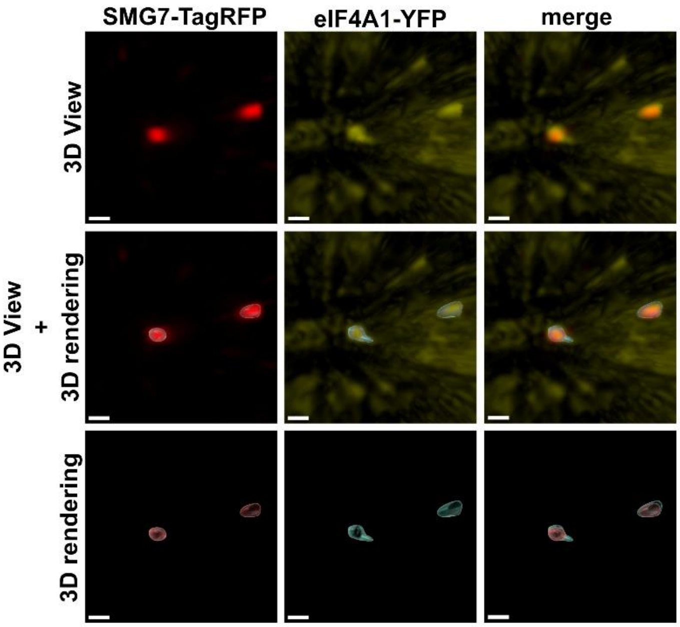

Supplement: Supplementary file 5 — Transparent Peer Review file [file 41467_2026_72218_MOESM5_ESM.pdf]
